# Picosecond quantum transients in halide perovskite nanodomain superlattices

**DOI:** 10.1038/s41565-025-02036-6

**Published:** 2025-10-29

**Authors:** Dengyang Guo, Thomas A. Selby, Simon Kahmann, Sebastian Gorgon, Linjie Dai, Milos Dubajic, Terry Chien-Jen Yang, Simon M. Fairclough, Thomas Marsh, Ian E. Jacobs, Baohu Wu, Renjun Guo, Satyawan Nagane, Tiarnan A. S. Doherty, Kangyu Ji, Cheng Liu, Yang Lu, Taeheon Kang, Capucine Mamak, Jian Mao, Peter Müller-Buschbaum, Henning Sirringhaus, Paul A. Midgley, Samuel D. Stranks

**Affiliations:** 1 Department of Chemical Engineering and Biotechnology, University of Cambridge, Cambridge, UK; 2 Department of Physics, Cavendish Laboratory, University of Cambridge, Cambridge, UK; 3 Department of Materials Science and Metallurgy, University of Cambridge, Cambridge, UK; 4 Forschungszentrum Jülich, JCNS at MLZ, Garching, Germany; 5 Chair for Functional Materials, Department of Physics, TUM School of Natural Sciences, Technical University of Munich, Garching, Germany; 6 Present Address: Institute of Physics, Chemnitz University of Technology, Chemnitz, Germany

**Keywords:** Single photons and quantum effects, Structural properties

## Abstract

The high optoelectronic quality of halide perovskites makes them suitable for use in optoelectronic devices and, recently, in emerging quantum emission applications. Advancements in perovskite nanomaterials have led to the discovery of processes in which luminescence decay times are below 100 picoseconds, stimulating the exploration of even faster radiative rates for advanced quantum applications, which have only been realized in III–V materials grown using costly epitaxial growth methods. Here we discovered ultrafast quantum transients with timescales of around two picoseconds at low temperature in bulk formamidinium lead iodide films grown via scalable solution or vapour approaches. Using a multimodal strategy, combining ultrafast spectroscopy, optical and electron microscopy, we show that these transients originate from quantum tunnelling in nanodomain superlattices. The outcome of the transient decays, that is, photoluminescence, mirrors the photoabsorption of the states, with an ultranarrow linewidth at low temperature that can reach <2 nm (~4 meV). Localized correlation of the emission and structure reveals that the nanodomain superlattices are formed by alternating ordered layers of corner-sharing and face-sharing octahedra. This discovery opens new applications leveraging intrinsic quantum properties and demonstrates powerful multimodal approaches for quantum investigations.

## Abstract (web summary)

Formamidinium lead iodide perovskite films exhibit picosecond-scale quantum transients (~2 ps), with their nanoscale superlattices defining energy levels that yield narrow emission lines and reveal the structure–emission relationship.

## Main

Halide perovskites have emerged as a promising class of materials for optoelectronic devices, including as solar cells, light-emitting diodes and X-ray detectors^1,2^. When considering tuning at the nanoscale, quantum confinement, combined with their optoelectronic properties, extends their application to a diverse array of fields including quantum emitters, which are critical components in the development of ultrafast communication, computing and sensing devices^3–5^. Ideal quantum emitters should possess both coherent photon production and ultrafast dynamics, both of which require control over the quantum confinement in the material. Single-photon emission has been reported in colloidal perovskite quantum dots, with sub-100 ps superradiance in superlattice structures^3,5^, propelling perovskites to the forefront of ultrafast quantum applications.

Beyond nanostructured perovskites, surprising peaked features above the bandgap have recently been reported in the absorption and emission spectra of bulk formamidinium lead iodide (FAPbI_3_) films at cryogenic temperatures^6,7^. These exotic features are proposed to originate from discrete optical transitions of electrons from quantum confined states, and the emission from these states is confirmed not to arise from lasing, as shown by the absence of threshold behaviour and spectral changes under varied excitation power^7^. Specifically in the work of Wright et al.^6^ the confinement length scale was estimated to be within 10–20 nm from a combination of ab initio calculations and measurements on films of differing thicknesses. However, owing to the steady-state nature of absorption spectroscopy^6^, and the temporal resolution limit of photoluminescence (PL) spectroscopy^7^, the precise photophysical evolution is yet to be explained. It has been speculated that the origin of the quantum confinement is from the formation of nanodomains that are either ferroelastic and/or ferroelectric, or δ-phase inclusions, although a direct structural understanding of these features remains lacking^6^.

In this Article we combine an ultrafast spectroscopic strategy with correlative optical and electron microscopy to report ultrafast quantum processes in FAPbI_3_. We directly reveal that the origins of the quantum effects are layered, nanotwinned α- and δ-phases, which, when periodic, can be regarded as a high-order hexagonal polytype.

## Picosecond quantum transients

**Fig. 1:**
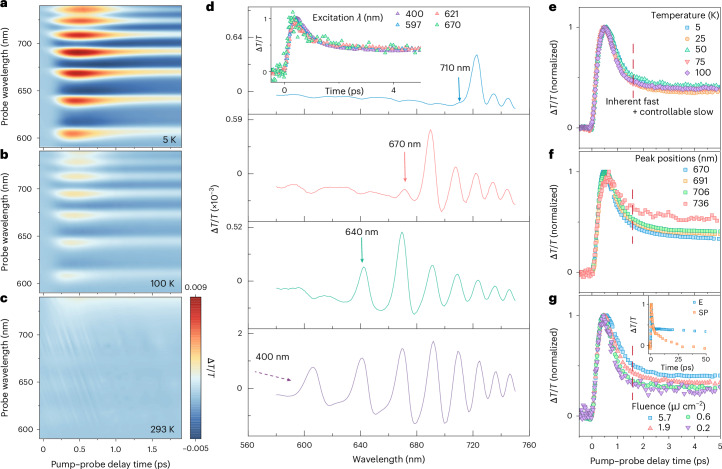
Bulk FAPbI_3_ thin films exhibit ultrafast quantum transients. **a**–**c**, Temperature-dependent TA maps of vapour-processed FAPbI_3_ films at 5 K (**a**), 100 K (**b**) and 293 K (**c**), showing that the discrete absorption peaks are attenuated as the temperature is increased. The colour scale is shared for comparison, ranging from 5 × 10^−3^ to 9 × 10^−3^, which is far above the Δ*T*/*T* sensitivity of ~10^−6^ of the home-built TA set-up. **d**, Excitation-wavelength-dependent absorption spectra within a consistent time window. Only peaks at lower energy are clearly observed. Temperature: 5 K. Inset: excitation-wavelength-dependent transients at 5 K and peak position 723 nm. **e**, Temperature-dependent transients at peak position 723 nm. **f**, Transients at different quantum peaks, excited using a fixed 400 nm laser beam with a pulse fluence of 5.7 μJ cm^−2^. **g**, Excitation-fluence-dependent transients following the peak at 723 nm, excited using 400 nm laser excitation. Inset: comparison of the transients between the solution-processed (SP) and evaporated (E) samples over a time window of 50 ps. In **e**–**g**, the dashed line marks the turning point at around 2 ps.

We monitored the ultrafast evolution of the quantized features (quantum transients) in bulk FAPbI_3_ on a picosecond timescale using temperature-dependent transient absorption (TA) spectroscopy, thus providing the time-dependent difference in photoabsorption of the material, before and after pulsed laser-pump photoexcitation. FAPbI_3_ bulk films were deposited on quartz substrates using thermal co-evaporation (Methods)^8^. We first used a pump energy of 3.1 eV (400 nm), well above the absorption onset of the FAPbI_3_ films (1.52 eV (817 nm); Supplementary Fig. 1). With the sample at 5 K, we observed particularly distinct wavelength- and time-dependent oscillatory features (red stripes in Fig. 1a). These stripes demonstrate that, above the bandgap, there exist quantized energy levels, and the signal from these excited states is attenuated over time on the order of picoseconds.

The quantum features emerged gradually as we decreased the temperature from ambient conditions to 5 K (Fig. 1a–c). Consequently, all spectra at low temperatures, extracted vertically from time zero to a point near the centre of the stripe, displayed an oscillatory pattern (Supplementary Fig. 2). The same emergence of the oscillatory pattern was observed in stabilized solution-processed FAPbI_3_ films (Supplementary Fig. 2).

Using the linked minima of the stripes as a baseline, we extracted the time evolution of the quantum peaks (Supplementary Fig. 4). After excitation with the same excitation density at different wavelengths but at the same temperature (Fig. 1d, inset), or at the same wavelength but at different temperatures (Fig. 1e), we find overlapping transients in the same quantum peak (723 nm). Such an overlap is further observed in the transients from other peaks (Supplementary Fig. 5). Therefore, the temperature- and excitation-independent transients of the excited quantum states are inherently determined, indicating intrinsic transients via either transition to a corresponding ground state or transfer to another energy level^9^.

To distinguish between these two possibilities, we set the excitation wavelength to the individual peak or valley positions along the oscillatory profile (Fig. 1d and Supplementary Fig. 6). Only signatures with a longer wavelength (lower energy) than the excitation wavelength are present in each spectrum, indicating the absence of shared ground states among the individual levels. Physically, this result implies that these peaks do not stem from different integer quantum numbers within the same confinement but from distinct confinement regions within the material^9^. Structurally, this implies that the quantum confinement is not a bulk property of the material but rather emerges from separate domains with dimensions sufficiently small to induce confinement, in line with the proposal of a confinement length scale of ~10–20 nm (ref. ^6^).

**Fig. 2:**
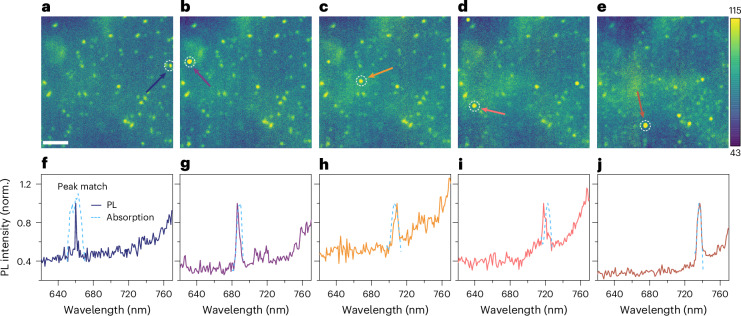
Bulk FAPbI_3_ thin films exhibit isolated sites of quantum emission with extremely narrow linewidths. **a**–**e**, Hyperspectral wavelength-selective PL maps of solution-processed FAPbI_3_ films, obtained at 5K using an excitation wavelength of 405 nm and an excitation power of ~1.8 mW cm^−2^, which exhibit individual emission spots at wavelengths of 660 nm (**a**), 686 nm (**b**), 706 nm (**c**), 718 nm (**d**) and 736 nm (**e**). Scale bar, 10 µm. The map is recorded from a fixed region of the film, and the wavelength of the individual map is denoted on each panel. The individual spots appear at different positions when the map is displayed at changing wavelengths (see also Supplementary Video 1). The colour scale denotes the PL intensity. The dashed circles indicated by arrows mark the locations where the PL spectra are acquired. **f**–**j**, PL spectra showing the emission peaks with extremely narrow FWHM values at the individual spots denoted by the white dashed circles in PL maps **a** (**f**), **b** (**g**), **c** (**h**), **d** (**i**) and **e** (**j**). The dashed cyan lines denote the region-selected TA peaks, of which the original data are normalized to unity. The TA and PL peaks show a precise match.

To further explore the independence of the peaks, we extracted the decay characteristics for each under identical excitation wavelength and temperature (Fig. 1f and Supplementary Fig. 7). At the peaks of longer wavelengths (lower energy), we observed a correspondingly longer decay time, which may mean that excited states transfer from higher to lower energy levels. However, such an assumption implies a close attachment between these regions, which could enable the transfer. Such a proximity is not guaranteed, as demonstrated by the distinct emission peaks observed in Fig. 2. Therefore, the distinct decays at different peaks indicate that the time evolution of the excited states can be an intrinsic character of the quantum confinement, by analogy to the size-dependent photocarrier lifetime of short-period GaSb/ErSb nanoparticle superlattices^10^.

To substantiate that the quantum levels arise from confinement within individual domains, we examined the decay behaviour at the same quantum peak (Fig. 1g). Notably, the decays for transients occurred within the initial 2 ps. Similar 2-ps transients were also observed in solution-processed films (Supplementary Fig. 8). Moreover, in both evaporated (Fig. 1g) and solution-processed (Supplementary Fig. 6) films, the excitation density does not influence the timescale of these fast transients, with the turnover point from a fast to a slow component remaining approximately at 2 ps. The constancy of the initial ultrafast process agrees with the quantum transients being intrinsic. By contrast, the slow part of the decay depends on the sample-processing method (Fig. 1g inset), showing a timescale of ~1 ns in evaporated films and ~50 ps in solution-processed films (Supplementary Fig. 7). Such variability of the long tail suggests that this second decay component is controllable and may relate to different trap densities. Such a combination of fast and slow components on picosecond timescales is consistent with the photocarrier lifetime in GaAs superlattices grown via molecular beam epitaxy, where the slow component is controlled by extrinsic effects (in that case, ErAs doping)^10,11^.

## Isolated emissions of the quantum transients

To understand the isolated transient processes originating from individual domains, we recorded cryogenic hyperspectral PL microscopy images. We see individual PL spots at distinct spatial locations, with the distributions being particularly stark in the solution-processed samples in which the emission points are spread randomly several micrometres away from each other within the PL map (Fig. 2a–e and Supplementary Video 1; see Supplementary Fig. 9 for similar qualitative behaviour on evaporated samples). The PL signals exhibit distinct and sharp emission peaks with ultranarrow full-width at half-maximum (FWHM) values of 1–5 nm (~4–10 meV), as depicted in Fig. 2f–j. These PL peaks align precisely with the corresponding peak segments of the photoabsorption spectrum (compare Fig. 1). The precise alignment indicates the absence of a Stokes shift, which is typically observed in perovskite films due to carrier relaxation in a continuous band. Hence, this finding further highlights the quantum nature of the observed peaks, underscoring their individual and isolated characteristics. With an increase in temperature from 5 K to 100 K, a slight wavelength shift occurs, consistent with the absorption peak shift. Concurrently, the intensity of the above-bandgap isolated emitters decreases. By contrast, the spatial positions of the emitters, along with the emission spread and FWHM, show no observable changes with temperature (Supplementary Fig. 10). This unchanged behaviour, in contrast to the broadening observed for the main bandgap peak, further suggests the independence of these quantum features from the bulk properties^12^.

## Ubiquitous spread of nanotwinning domains

To gain a structural understanding of the isolated emission spots, spatially correlated hyperspectral PL and scanning electron diffraction (SED), a variant of four-dimensional scanning transmission electron microscopy, measurements were performed at both ambient and cryogenic temperatures. We studied FAPbI_3_ thin films thermally evaporated onto silicon nitride (SiN_*x*_) membrane grids, which are compatible with both measurement modalities^13^.

**Fig. 3:**
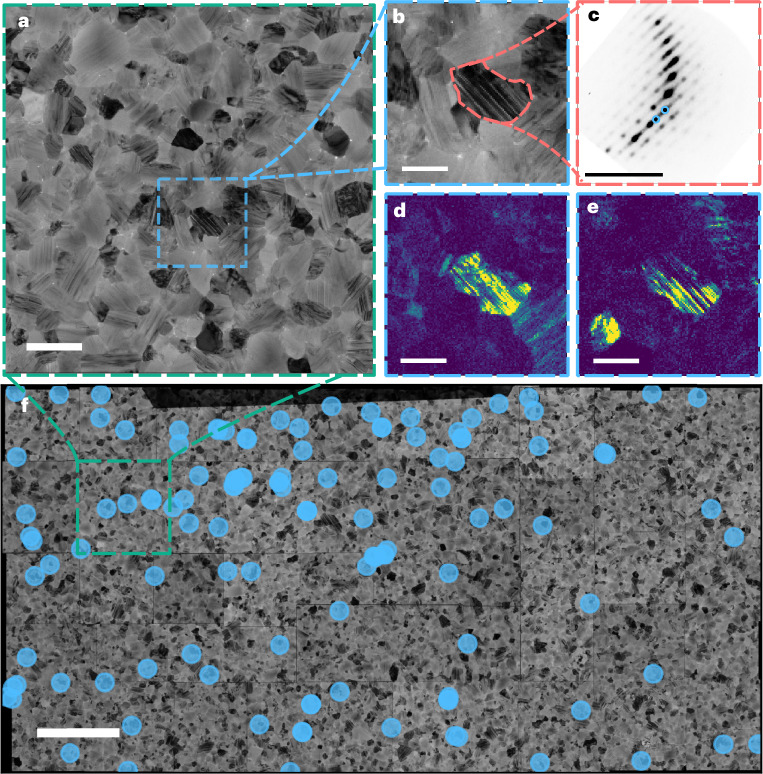
{111}_c_-type nanotwinning is ubiquitous in bulk FAPbI_3_ thin films as observed via spatial nanoscale structural maps. **a**, VBF image formed from a typical area of a vapour-deposited FAPbI_3_ film (as displayed in **f**), obtained at ambient temperature. Scale bar, 500 nm. **b**, Expanded image of the region marked in **a**. Scale bar, 200 nm. **c**, The average diffraction pattern from the grain highlighted in **b**. Scale bar, 1 Å^−1^. **d**,**e**, False-colour VDF images formed when ‘apertures’ are placed over the spots corresponding to opposing twins, marked by the blue circles in **c**. Scale bars, 200 nm. **f**, VBF images from the SED dataset obtained at ambient temperature and stitched together to form a large-area map with the blue points exhaustively showing where grains are oriented close to a <110>_c_ zone axis such that the observation of nanotwinning is most pronounced. Scale bar, 3 μm.

At both cryogenic and ambient temperatures, when virtual bright-field (VBF) images are formed by taking intensity only from the direct beam in the diffraction pattern, intragrain planar features are observed throughout the entirety of the grains (an expanded example is shown in Fig. 3a,b and Supplementary Figs. 14 and 15). These features are explained by the presence of stacking faults along a common crystallographic direction. Indeed, diffuse scattering along a <111>_c_ direction is also present in the corresponding diffraction patterns, explained by the occurrence of nanoscale planar features, leading to the elongation of reciprocal space lattice points into rods (Fig. 3c). In addition to this, archetypal diffraction patterns consisting of two overlaid <110>_c_ zone-axis patterns sharing spots along a <111>_c_ direction are observed, suggesting the presence of twinning along the said <111>_c_ direction (Supplementary Fig. 16). It is important to note that a perfect {111}_c_-type twin boundary is equivalent to a planar layer of face-sharing octahedra (δ-phase) separating regions of corner-sharing octahedra (α-phase). Furthermore, depending on the number of face-sharing layers present, the planar defect can be regarded as a twin or stacking fault^14^, and henceforth we shall use the all-encompassing term ‘nanotwinning’ to broadly describe the different types of these planar defects.

Furthermore, the diffuse scattering along a <111>_c_ direction is perpendicular to the planes observed in the VBF images (Fig. 3b,c). In addition, if virtual dark-field (VDF) images are created by placing two apertures over complementary reflections, planar stripes that approximately anticorrelate with respect to each other are observed (Fig. 3d,e and Supplementary Fig. 17, shown with an inverting colour map). The choice of which reflections to place apertures over is somewhat arbitrary, and similar anticorrelating contrast can be obtained from many sets of complementary spots (Supplementary Fig. 18). To understand the contrast in these VDF images, masks were applied to the data to extract the averaged diffraction pattern from each complementary reflection (Supplementary Fig. 17). As these patterns still display diffuse intensity and features indicative of nanoscale twins, we conclude that the SED probe is probably encompassing multiple twin boundaries at each pixel position and the contrast observed in the VDF images is from {111}_c_-type intragrain nanotwinning, which is largely aperiodic throughout the grain (Fig. 3d,e and Supplementary Fig. 18). Furthermore, this provides an upper limit on the length scales on which nanoscale twins form to be the size of our SED probe, which we estimate to be ~5 nm. Linking the structural and photophysical observations presented thus far, we propose that many of these coplanar {111}_c_-type nanotwins form an array of wells and barriers analogous to a Kronig–Penney (KP)-type quantum superlattice within grains of alternating corner- and face-sharing octahedra (Fig. 3f), as previously theorized^6^. This is further consistent with previous literature reports, with the occurrence of {111}_c_ twinning being linked to poor solar cell performance^14–18^. Reports have congruently shown that, when quantum confinement effects are observed, this is detrimental to solar cell performance^19^. Intuitively, this is consistent with our observation as when the nanotwins are oriented in the plane of the film they will act as barriers to the vertical movement of charge carriers, leading to worse device efficiency^20^. Furthermore, it has been shown that A-site alloying with Cs, which is known to reduce the prevalence of nanotwin domains^21^, attenuates the above-bandgap oscillations in the absorption and emission spectra^7^.

**Fig. 4:**
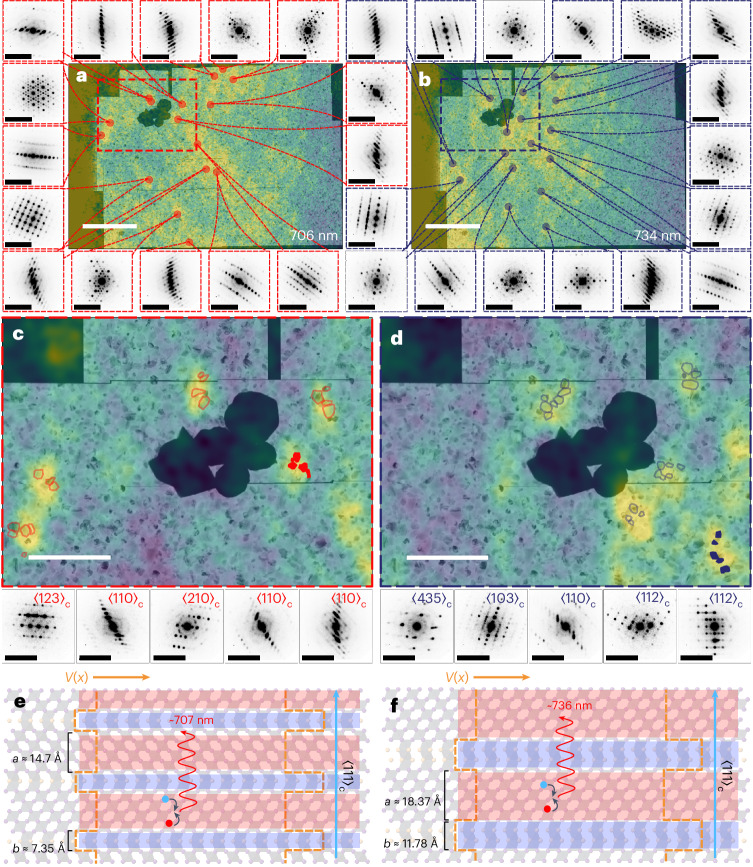
Spatial correlations between local photophysics (PL) and structure (SED) reveal bright isolated emission arising from grains which show nanoscale twinning. **a**,**b**, Stitched SED (90 K; as small insets around the main image) correlated with the hyperspectral PL (4 K) of a vapour-processed FAPbI_3_ film. Maps are shown from the same scan area of the PL at 706 nm (**a**) and 734 nm (**b**). For each wavelength the PL intensity shown is at a 65% threshold of the maximum value. Scale bars, 5 μm (central image) and 1 Å^−1^ (surrounding insets). **c**,**d**, The overlaid hyperspectral and SED data expanded from **a** (**c**) and **b** (**d**) with clusters of grains from each emissive region shown. Diffraction patterns from the shaded grains in each main image are shown as insets below, indexed to a pseudocubic unit cell while including the presence of nanoscale twins, with complete correspondences shown in Supplementary Figs. 27 and 28. A Gaussian blur was applied to the each hyperspectral PL frame to remove high-frequency noise from the camera. Scale bars, 2 μm and 1 Å^−1^. **e**,**f**, The proposed structural model where isolated emitters of ~707 nm (**e**) and ~736 nm (**f**) are observed if ordered twin domains are present, with a representation of the change in potential *V*(*x*) shown with dashed orange lines, modulated along a <111>_c_ direction (blue arrow) corresponding to the corner and face sharing structure (shown with pink and blue shading, respectively). We propose this ultimately dictates the emission (shown with wavy arrows) due to the recombination of charge carriers (shown by red and blue circles) within the potential.

Using the capability of the local mapping, we now explore the spatial distribution of the nanotwinning across the film. Given the enormity and richness of the SED datasets acquired (totalling 428 Gb after compression from 122 individual SED scans), manually indexing and interpreting each diffraction pattern is intractable. We therefore cluster each SED scan using the simple linear iterative clustering (SLIC) methodology as described in Supplementary Text 2^22,23^. This takes the 262,144 (512 × 512) individual patterns recorded in a single SED measurement to approximately 500 averaged patterns. Once the diffraction patterns are clustered, we observe the extensive presence of {111}_c_-type nanotwinning through the observation of many diffraction patterns with diffuse scattering consistent with the proposed structural model (Supplementary Figs. 14c and 15c). Similarly, planar features in reconstructed VBF images are also observed across the entirety of the area imaged (27 × 14 μm; Fig. 3f and Supplementary Figs. 14a and 15a). Furthermore, these observations are consistent across multiple samples prepared in different batches (Figs. 3f and 4a,b), enabling us to conclude that {111}_c_ intragrain nanoscale twins are a highly ubiquitous structural feature in FAPbI_3_ films. We also note the presence of octahedral tilt upon cooling, which is discussed in Supplementary Text 3.

## Correlation of the localized emission and nanotwinning superlattice

To connect the structural observations with the localized photophysical properties, SED measurements were performed on the same area as that imaged via hyperspectral PL at cryogenic temperatures using gold (Au) fiducial markers to spatially correlate between datasets from the two techniques (Fig. 4c,d and Supplementary Text 4)^24^. We correlate the cryo-SED recorded between 80 and 90 K with hyperspectral PL recorded at 4 K and 80 K (Fig. 4 and Supplementary Fig. 20) but note that the spatial location of pronounced emission is largely constant between temperatures (Supplementary Fig. 10). We show the hyperspectral PL maps of the same region in Fig. 4a,b, which represent the emission at 706 and 734 nm, respectively, and observe local above-bandgap oscillations in the PL spectra throughout the films (Supplementary Fig. 9), in addition to particularly bright isolated emitters at each wavelength. Owing to the spatial resolution difference between the two imaging modalities, many SED scans were acquired before being stitched together so that a large area of the hyperspectral data could be covered (Fig. 4a,b and Supplementary Figs. 14 and 15). To do this accurately, as shown in Figs. 3f and 4a,b, a method that combines computer vision techniques was used, as outlined in Supplementary Text 4.

Crucially, when the cryo-hyperspectral PL and cryo-SED datasets are overlaid (illustrated in Fig. 4a,b and Supplementary Fig. 20), the structural properties of the emission can be understood. First, we note that grains oriented close to a <111>_c_ (equivalent to a <001>_h_) zone axis of FAPbI_3_ are prevalent in areas of pronounced isolated emission, and that this is true for multiple separate sample batches (Fig. 4a,b and Supplementary Fig. 24). We propose that the isolated emission is predominately dictated by the ordering and periodicity of the superlattice. Considering the diffraction patterns correlated to emitters at ~706 nm, the fact that superstructure reflections consistent with octahedral tilting appear in <111>_c_ patterns at 80 K (Fig. 4a and the inset of Supplementary Fig. 20e), and that no more additional reflections are observed, we assign these grains to be either a 3C, 6H, 9R, 12H, 12R or 18H polytype (or a combination thereof; Supplementary Fig. 22)^20,25^. If grains around isolated emitters are examined, we confirm the presence of {111}_c_-type nanoscale twinning, consistent with our structural model (Fig. 4 and Supplementary Figs. 27 and 28). Intuitively, we expect the ordering of a KP superlattice to influence the emission intensity. Thus, we examine grains oriented close to a <110>_c_ zone axis and form VDF images as done previously. When VDF images are formed we observe that, in some grains, instead of anticorrelating striations, the contrast is more homogeneous, indicative of enhanced ordering between corner- and face-sharing layers in areas of bright emission when compared with the remaining film (Supplementary Figs. 25 and 26). This is consistent with the proposed model of periodic ordering of twin domains leading to bright emission. Nevertheless, we note that if grains are oriented away from the <110>_c_ direction we are unable to form relevant VDF images, rendering them unavailing. We further note that definitive assignment of the emission to a structural feature is challenging due to the resolution differences between the two techniques, and that the emission may originate from an ensemble of grains in the region of interest. Finally, the assignment of a higher-order polytype is in agreement with previous literature reports, where density functional theory calculations for CsPbI_3_ found that the phases have a direct bandgap (from 12H onwards) whereas the lower-order equivalents have indirect bandgaps, and thus would not be expected to be emissive^20^. We also note that the face-sharing polytypes of CsPbI_3_ are not thermodynamically favourable compared with the edge-sharing equivalents, as observed in FAPbI_3_ (ref. ^26^), so quantum confinement phenomena are not expected to be observed in the purely Cs-based systems.

Considering the proposal that the isolated emission originates from a superlattice, the wavelength of emission (706 or 734 nm, as presented) should be modulated by the spacing between corner- and face-sharing layers. Thus, we attempt to understand the size, ordering and periodicity of the nanotwinned array collectively from the ensemble of diffraction patterns collected and the reconstructed VBF and VDF images for isolated emission at both 706 and 734 nm. If the diffraction pattern of a perfectly regular superlattice viewed along a [100]_h_ zone axis is examined, the Bragg peaks along the twinning direction converge into an unresolvable streak as the number of corner-sharing layers between face-sharing nanotwins increases (Supplementary Fig. 21). It is therefore difficult to discern between a high-order hexagonal polytype and the purely diffuse scattering which can result from a disordered arrangement of nanotwins. Furthermore, as each SED pixel (6.5 nm) is likely to encompass multiple twin boundaries, we can only ascertain an estimate of the upper limit (~5–10 nm) for the thickness of corner-sharing octahedral layers upon which the superlattice forms, which is then used to inform subsequent calculations. In Fig. 4e, we show an illustrative structure of the {111}_c_-type nanotwins viewed in cross-section into the film; if this is considered to be perfectly ordered, it can be viewed as a high-order hexagonal polytype (12H as presented, consistent with the assignment of the observed <111>_c_ patterns). We propose that the alternating α- and δ-phases serve as the quantum well and barrier, respectively, in the KP model. By assuming that the width values of the well and barrier occur at integer numbers of the octahedral layer spacing (*u*) along a <111>_c_ direction (intervals of *u* = 0.37 nm at room temperature), we find that the simulated quantum well (*a*) and barrier (*b*) parameters for modelling a 12H polytype are *a* = 1.48 nm (4*u*) and *b* = 0.74 nm (2*u*). By implementing this in the KP model, along with an effective mass of 0.21*m*_0_ (where *m*_0_ is the mass of an electron)^27^, we calculate a first discrete energy level from confinement to be 707 nm, which is consistent with the experimentally observed peak at 706 nm. Similarly, the 734 nm emission could originate from a combination of *a* = 1.85 nm (5*u*) and *b* = 1.11 nm (3*u*). Here the quantum peak progression arises from the layer progression in the KP configuration, rather than from *n*-index progression, as discussed in Supplementary Text 5. Critically, this layer progression agrees with the fact that PL emission will in general only occur at the first quantum state (*n* = 1), as demonstrated in III–V superlattices^28^. The exclusion of n-index progression as a viable mechanism is also based on the observation of multiple polytypes. If quantum number progression were the dominant mechanism, the structural diversity of polytypes would produce disordered spectral features—contradicting the reproducible alignment of TA and PL peaks shown in Fig. 2. We note that an 18H polytype is another example that would yield consistent results, and that the calculation depends on the effective mass value used, making it difficult to unambiguously assign a specific polytype structure to a specific quantum peak (Supplementary Text 5 and Supplementary Table 1).

Finally, we seek insight into the transients with these structural and energy parameters. The ultrafast picosecond decays cannot be explained by Auger recombination (which would be on timescales of >100 ps and be fluence-dependent)^29^, defect-mediated trapping (which would be suppressed at 4 K and dependent on the sample) or phonon scattering (temperature-dependent), pointing to an intrinsic origin. Given that the observed ultrafast transients are ~2 ps, regardless of the material conditions, and barrier thicknesses are below 1 nm (ref. ^30^), a reasonable mechanism to facilitate the ultrafast transients of the excited states from the quantum levels involves quantum tunnelling. We therefore apply a semiclassical approach using the Wentzel–Kramers–Brillouin (WKB) approximation. This approach takes the states as classical particles for calculating the frequency of bouncing at the barriers but treats the states as wavefunctions for estimating the chance of quantum tunnelling through the barriers. The lifetimes of the states are inherently determined by quantum tunnelling and are modulated by the width of the well and barrier. By applying the WKB approximation, we estimate the mean lifetime of the excited carriers in the quantum confined levels to be approximately ~2.0 ps for a peak at 706 nm and ~2.8 ps for a peak at 756 nm. This estimation aligns well with the experimental values presented in Fig. 1 (Supplementary Text 6). Such consistency of the experimental data and calculated values based on the KP model, along with the timescales (accounting for the picosecond transients and the WKB approximation), supports the concept that the observed quantum transients relate directly to the nanotwinned structures.

## Conclusions

Both the KP model and WKB approximation are fundamental concepts in quantum physics. Our work demonstrates that these concepts, that is, ultrafast dynamics of photoexcited carriers within a KP superlattice, can be intrinsically obtained in an inexpensive and easily fabricated metal halide perovskite bulk film. As the inherent lifetime is defined by structural parameters, the quest for achieving ultrafast lifetimes has in the past relied on constructing ideal KP superlattices, such as via expensively grown molecular beam epitaxy or the introduction of extra doping as a modification step^11^. The discovery of ~2 ps quantum transients that originate from the intrinsic nanotwinning highlights opportunities for facile strategies to achieve nanoscale control of ultrafast quantum emitters. Future work will focus on developing strategies for the controllable growth of twinned nanostructures into uniform, vertical arrays, maintaining a well-ordered and common alternating number so that ultrafast dynamics can be achieved at a fixed emission wavelength. Such precisely engineered periodic architectures could amplify collective quantum effects through enhanced interdomain coupling, potentially stabilizing coherent states against thermal disruption; we note, however, that such coupling is not required to observe the quantum emission effects, as shown from the isolated quantum emission presented in this Article. Such a uniform arrangement could be further applied for circuit integration to realize ultrafast device applications. With the direction of growth under control, the transition dipole moment of emission could also be further analysed, for example, by applying a polarized excitation^31,32^.

Furthermore, our multimodal strategy, which combines ultrafast spectroscopy and correlative optical and electron microscopy, serves as a generalized platform for other material systems. It offers a top-down routine, capable of resolving ultrafast processes and the correlated nanoscale structure. By leveraging these computer vision-based analysis pipelines and clustering methodologies, and by extending the approach to techniques that overcome the optical diffraction limitation, we foresee investigating the correlated structure–photophysics of a wide array of advanced materials with extremely rich datasets. Furthermore, the correlation of structural attributes and optoelectronic properties offers a tool for precision engineering at the nanoscale. This could lead to the discovery of materials with tailored properties for specific applications in quantum computing, solar energy harvesting and light-emitting devices, among others.

## Methods

### (Cryo-) transient absorption spectroscopy

TA spectroscopy measurements were performed using a home-built platform. The output from a titanium:sapphire amplifier system (Solstice Ace, Spectra-Physics) was divided into pump and probe beam paths. This system operates at 1 kHz and generates pulses of approximately 100 fs. The 400 nm pump pulses were produced by directing the 800 nm fundamental beam of the Solstice Ace system through a 1-mm-thick second harmonic generation β-barium borate crystal (Eksma Optics). Pump pulses with tunable wavelengths (those used in Fig. 1) were generated by directing the 800 nm fundamental beam through a TOPAS optical parametric amplifier (Spectra-Physics) and with subsequent suitable spectral filtering. A chopper wheel blocked every other pump pulse to provide pump-on and pump-off referencing, while a computer-controlled mechanical delay stage (DDS300-E/M, Thorlabs) adjusted the temporal delay up to 2 ns between the pump and the probe. A visible broadband beam (525–775 nm) was generated in a custom-built noncollinear optical parametric amplifier (or NOPA), and the white light was divided into two probe and reference beams using a 50/50 beamsplitter. The reference beam, which did not interact with the pump, passed through the sample. With this set-up, it was possible to measure small signals with a normalized change in the transmitted probe intensity (Δ*T*/*T*) of approximately 10^−5^. The transmitted probe and reference pulses were collected with an InGaAs dual-line array detector (G11608-512DA, Hamamatsu; combined with a Shamrock SR-303i-B spectrograph (Andor)), which was driven and read out using a custom-built board (Entwicklungsbüro Stresing).

The temporal resolution is limited by the pump pulse duration only. For the 400 nm pump, the pulse duration is ~100 fs, and for visible NOPA pumps (all others that are not 400 nm), the pulse duration is ~120 fs—overall, they are definitely all less than 150 fs. We stretched the probe beam in time using fused silica (24 mm) and quartz with an antireflective coating (24 mm) to obtain broadband amplification from 530 to 780 nm. The probe beam does not affect the temporal resolution, because we collect wavelength-dependent broadband probe data and we have chirp correction for each probe wavelength, such that the temporal stretch due to group velocity dispersion of the probe is corrected for each wavelength in the software. As per the measurements, the camera is time-independent and collects all of the light from the probe, at each position that the probe has a certain delay with respect to the pump due to different optical paths in real space. In addition, we can clearly see that the rise of the TA signal is sharper (steeper) than the decay, and this also indicates that our decay timescale is not limited by the instrument response function/temporal resolution.

Our home-built TA spectrometer is specifically designed to suppress stimulated emission through two key design features: (1) the absence of time-resolved detection systems (for example, streak cameras) and (2) intentional software-based filtering that excludes all emission-related signals, including pump scattering and sample PL. Before time-resolved measurements, baseline counts were systematically recorded with the pump beam active to account for residual emission and scattering. This baseline-subtraction protocol ensures the isolation of pure absorption signals during data processing, effectively decoupling absorption dynamics from emissive processes.

Temperature-dependent TA measurements were performed by mounting the sample in a closed-circuit pressurized helium cryostat (Optistat Dry BL4, Oxford Instruments) placed at the focal point of the probe and reference beams. The cryostat was driven using a compressor (HC-4E2, Sumitomo) and a temperature controller (MercuryiTC, Oxford Instruments). The vacuum level inside the cryostat was below 10^−5^ mbar.

### (Cryo-) scanning electron diffraction

SED was performed using a ThermoFisher Spectra 300 scanning transmission electron microscope operated at an accelerating voltage of 200 kV and a convergence angle of 300 μrad, giving a probe size of ~5 nm when a scan step size of 6.49 nm is used. A Quantum Detectors Merlin/Medipix3 single-chip direct electron detector with a 256 × 256 pixel array was used to record diffraction patterns with a dwell time of 1 ms and current of ~3 pA, equating to a fluence of ~11 e Å^−2^ which was maintained throughout the measurement. ThermoFisher Maps software (v3.20.1) was used to record annular dark-field images concurrently with SED data, with an inner and outer detector collection semiangle of 62 and 200 mrad, respectively. Reciprocal space and rotation calibrations were performed using a MAG*I*CAL® calibration sample. For measurements at cryogenic temperatures, a Gatan 613 cryogenic holder was used with liquid N_2_ (to achieve low temperatures); once cooled, the temperature stabilized at ~90 K. To conduct a damage study, a JEOL ARM300CF, E02 instrument at ePSIC, Diamond Light Source, with a 515 × 515 Merlin/Medipix quad-chip detector was used to record diffraction patterns and operated with similar acquisition parameters to those of the correlative study. Post-acquisition analysis of SED data was performed using pyXem v.0.16, py4DSTEM v.0.14 and Single Crystal 5 (v5.0.0) (Supplementary Text 1)^33,34^.

### Au marker synthesis

Au markers were synthesized^24,35^ by dissolving HAuCl_4_·3H_2_O (0.024 g) in ethylene glycol (25 ml) before leaving the solution to be stirred at 350 revolutions per min (r.p.m.) at 70 °C for 30 min. Aniline (1.12 ml, 0.1 M) in ethylene glycol was then added, and a brown suspension formed instantaneously. Stirring was then stopped but heating was continued for 3 h. After this time the resulting suspension was cooled to ambient temperature and the supernatant removed. Ethanol (20 ml) was then added, and the resulting suspension bath sonicated for 1 min before being centrifuged at 6,280 × *g* for 3 min (equivalent to 7,500 r.p.m. with a 10 cm rotor radius) and the supernatant discarded. A further two washing steps were carried out whereby ethanol (10 ml) was added, and the previous centrifuging step repeated. Finally, the resultant fiducial markers were dispersed in cholobenzene (1 ml), and 10 µl was spin-coated at 1,000 r.p.m. for 30 s onto a glass substrate to assess the size and coverage. These conditions also proved optimal for obtaining an adequate coverage of markers on single-window SiN_*x*_ transmission electron microscopy (TEM) grids (NT025X, Norcada).

### (Cryo-) X-ray diffraction

A PheniX cryostat was used for X-ray diffraction (XRD) analysis. This closed-cycle helium system, designed for low-temperature powder diffraction, enables the measurement of a flat, static sample between 12 and 310 K with automated temperature control (±1 K) through the XRD control software. The cryostat houses a two-stage Gifford McMahon cooler, which operates with a sealed helium gas circuit, ensuring no helium consumption. Sample cooling is achieved through heat conduction, with temperature measured at the sample stage. The system minimizes heat leakage with a radiation shield and a sturdy lid with X-ray-transparent windows. The PheniX Front Loader variant enables the cold-loading of samples, offering quick turnaround. XRD was carried out using a Bruker D8 Advance powder X-ray diffractometer. The XRD facility is equipped with Bruker EVA software, which is used for phase identification and qualitative analysis. In addition, it utilizes Bruker Topas software for conducting quantitative analyses.

### (Cryo-) hyperspectral microscopy

Wide-field, hyperspectral microscopy measurements were performed using an IMA system (Photon etc). Measurements were carried out with an Olympus LMPlanFL N 100× (NA = 0.8) or a Nikon TU Plan Fluor 20× (NA = 0.45) objective lens. The sample was kept under vacuum for the temperature-dependent experiments. Excitation was via a 405 nm continuous-wave laser (unpolarized), which was filtered out using a dichroic longpass filter for the detection. The emitted light was directed towards a volume Bragg grating, which dispersed the light spectrally onto a CCD (charge-coupled device) camera. The detector was a 1,040 × 1,392 resolution silicon CCD camera, kept at 0 °C using a thermoelectric cooler, and has an operational wavelength range of 400–1,000 nm. By scanning the angle of the grating relative to the incident light, the spectrum of light coming from each point on the sample could be obtained.

For the temperature-dependent experiments, the sample was fixed with silver paste to the cold finger of a cryostat (HiRes, Oxford Instruments) cooled with liquid helium. The cryostat was attached to the microscope with a self-made holder that enabled focus correction. The sample was held at the set temperature for at least 15 min before every measurement.

### (Cryo-) ultraviolet-visible spectroscopy

Variable-temperature UV-visible measurements were collected using a Varian Cary 6000i dual-beam spectrometer and an optical cryostat (OptistatCF-V, Oxford Instruments) equipped with quartz windows. Measurements were collected in transmission geometry under high vacuum (~10^−6^ mbar).

### (Cryo-) WAXS and SAXS

Small-angle X-ray scattering (SAXS) and wide-angle X-ray scattering (WAXS) experiments were performed at the SAXS-WAXS laboratory beamline KWS-X (XENOCS XUESS 3.0 XL system) of JCNS at MLZ. The MetalJet X-ray source (Excillum D^2+^) with a liquid-metal anode was operated at 70 kV and 3.57 mA with Ga Kα radiation (wavelength *λ* = 1.314 Å). Thin-film samples were measured using a temperature-controlled stage (HFS350, Linkam) with a liquid-nitrogen pump which achieves an ultralow temperature of −150 °C. Thesample-to-detector distances are from 0.1 to 1.70 m, which cover the scattering vector *q* range from 0.003 to 4.5 Å^−1^ (*q* = (4π/*λ*)sin(*θ*), where 2*θ* is the scattering angle). The SAXS patterns were normalized to an absolute scale and azimuthally averaged to obtain the intensity profiles, and the glass background was subtracted.

### Solution deposition of FAPbI_3_ thin films

*N*,*N*-Dimethylformamide (anhydrous, 99.8%), dimethyl sulfoxide (anhydrous, 99.9%) and ethylenediaminetetraacetic acid (anhydrous, 99.99%) were purchased from Sigma-Aldrich and used without further purification. Formamidinium iodide (FAI, 99.9%) was purchased from Greatcell Solar Materials and was used without further purification. Lead iodide (PbI_2_; 98%) was purchased from TCI and used without further purification. A mixture of PbI_2_ (0.346 g, 1.5 mmol), FAI (0.155 g, 1.8 mmol) and ethylenediaminetetraacetic acid (5 mol% relative to PbI_2_) was dissolved in dimethyl sulfoxide (0.5 ml) in a nitrogen-filled glovebox. This mixture was continuously stirred and heated at 75 °C until a clear dark-yellow solution was obtained. Quartz substrates were cleaned with detergent (Decon 90), deionized water, acetone and isopropanol in an ultrasonication bath for 15 min at each. The clean substrates were treated with UV-ozone for 15 min. The solution was then applied to these substrates in a nitrogen-filled glovebox using a spin-coating process (4,000 r.p.m. for 40 s). To ensure the uniformity of the film, nitrogen gas was blown onto the surface of the spinning films for 20 s, starting 5 s after the beginning of the spinning process. The nitrogen gun was initially positioned ~7 cm away from the film for the first 10 s, and then the distance was reduced to ~5 cm for the remaining 10 s. The spin-coated films were then annealed at 150 °C for 1 h, still in a nitrogen-filled glovebox

### Vapour deposition of FAPbI_3_ thin films

The quartz substrates were cleaned in the same way described above. The SiN_*x*_ TEM grids were placed on the quartz substrates using carbon tape. Vapour deposition was carried out using a CreaPhys PEROvap evaporator inside an MBraun N_2_ glovebox (O_2_ and H_2_O levels <0.5 ppm) to avoid exposure of the precursors and deposited films to oxygen and water during sample fabrication and handling. The evaporator chamber was pumped down to a pressure below 2 × 10^−6^ mbar for all depositions. The evaporator system was specifically designed with a cooling system that maintains the evaporator walls, source shutters and shields at −20 °C throughout the entire process. This functionality minimizes re-evaporation of the precursors and cross-contamination between sources, ensuring fine control over the evaporation rates and high reproducibility.

For FAPbI_3_ deposition, PbI_2_ (>98% trace metal basis) and FAI, without further modification, were added to two separate crucibles. For both the PbI_2_ and FAI, fresh powders were used for every deposition. The tooling factor of each chemical was calibrated by checking the film thicknesses via profilometry inside an N_2_-filled glovebox (DEKTAK XT profilometer, Bruker). Two quartz crystal microbalances mounted on the top of the vapour sources enabled us to monitor the deposition rate of each source so that the composition could be controlled.

The rate of evaporation was between 0.34 and 0.60 Å s^−1^ for PbI_2_ and between 0.68 and 1.50 Å s^−1^ for FAI. The substrate temperature was maintained at around 18 °C. The distance between evaporator sources and substrate holder was approximately 0.35 m. We observed minimal change in the substrate temperature (<1 °C), and the chamber was typically at a pressure of less than 2.0 × 10^−6^ mbar for the entire duration of each deposition step. Once the films were removed from the evaporator, they were immediately annealed on a hotplate within the same N_2_-filled glovebox at 150 °C for 20 min.

## Online content

Any methods, additional references, Nature Portfolio reporting summaries, source data, extended data, supplementary information, acknowledgements, peer review information; details of author contributions and competing interests; and statements of data and code availability are available at 10.1038/s41565-025-02036-6.

## Supplementary information

Supplementary Information Supplementary Figs. 1–44, Table 1 and Texts 1–6.

Supplementary Video 1 Wavelength dependent emergence of bright spots in the hyperspectral photoluminescence map.

## Data Availability

The data that support the findings of this study are available to download at the University of Cambridge Apollo Repository at 10.17863/CAM.122101.

## Code availability

SLIC clustering code can be found at https://github.com/TomSelby/hyperSLIC. Stitching code can be found at https://github.com/TomSelby/hyper_stitch.
