# Supplementary material for: Picosecond quantum transients in halide perovskite nanodomain superlattices
Source: Nat Nanotechnol. 2025 Oct 29;20(12):1771–8. doi: 10.1038/s41565-025-02036-6 (PMC12727500; doi:10.1038/s41565-025-02036-6)
Supplement: Supplementary file 1 — Supplementary Figs. 1–44, Table 1 and Texts 1–6. [file 41565_2025_2036_MOESM1_ESM.pdf]

nature nanotechnology

Supplementary information https://doi.org/10.1038/s41565-025-02036-6

Picosecond quantum transients in halide perovskite nanodomain superlattices

In the format provided by the authors and unedited

Supplementary information for

Picosecond Quantum Transients in Halide Perovskite Nanodomain Superlattices Dengyang Guo[1,2]*, Thomas A. Selby[1]*, Simon Kahmann[1†], Sebastian Gorgon[2], Linjie Dai[1,2], Milos Dubajic[1], Terry Chien-Jen Yang[1], Simon M. Fairclough[3], Thomas Marsh[2], Ian E. Jacobs[2], Baohu Wu[4], Renjun Guo[5], Satyawan Nagane[1], Tiarnan A. S. Doherty[1,3], Kangyu Ji[2], Cheng Liu[2], Yang Lu [1,2], Taeheon Kang[1], Capucine Mamak[1], Jian Mao[1], Peter Müller-Buschbaum[4,5], Henning Sirringhaus[2], Paul A. Midgley[3], Samuel D. Stranks[1,2]✉

Corresponding author: sds65@cam.ac.uk

The PDF file includes:

Supplementary Fig. 1 | Evaporated $FAPbI_3$ films exhibit good quality.
Supplementary Fig. 2 | Temperature-dependent TA maps of solution-processed $FAPbI_3$
Supplementary Fig. 3 | Temperature dependence of the quantum peaks and bandgap.
Supplementary Fig. 4 | Extraction method for the transient peaks from TA data.
Supplementary Fig. 5 | Temperature-dependent TA transients of evaporated $FAPbI_3$ thin film, complementary to Fig.1.
Supplementary Fig. 6 | TA Recordings of the stabilized solution-processed $FAPbI_3$ thin film.
Supplementary Fig. 7 | Full TA decays at the timescale of 2 ns for the evaporated and solution-processed samples
Supplementary Fig. 8 | The TA transient comparison between the solution-processed and evaporated $FAPbI_3$ samples over the timescale of 5 ps.
Supplementary Fig. 9 | PL maps and local emission spectra of evaporated and solution-processed $FAPbI_3$ samples under different post-treatment conditions.
Supplementary Fig. 10 | PL maps above the bandgap energy taken at different temperatures of a $FAPbI_3$ film deposited on a SiN_x TEM grid.
Supplementary Fig. 11 | Temperature dependent X-ray diffraction of the solution-processed $FAPbI_3$ thin film.
Supplementary Fig. 12 | Temperature-dependent wide-angle X-ray scattering (cryo-WAXS) of a solution-processed $FAPbI_3$ thin films.
Supplementary Fig. 13 | Temperature dependent small-angle X-ray scattering (SAXS) results reveal structure information on the evolution of the structure from 1 to 50 nanometers.
Supplementary Fig. 14 | Stitched SED data recorded at ambient temperature.
Supplementary Fig. 15 | Stitched SED data recorded at ~90K.
Supplementary Fig. 16 | A kinematical simulation of two twinned $[110]_c$ zone axis patterns rotated such that the $(\bar{1}11)_c$ and $(1\bar{1}1)_c$ reflections are shared and overlaid onto an experimental pattern taken at ambient temperature.
Supplementary Fig. 17 | SED data of thermally evaporated $FAPbI_3$ on SiNx grids recorded at ambient temperature.
Supplementary Fig.18 | Demonstration that similar anticorrelating contrast can be formed from many sets of complementary spots.

Supplementary Fig. 19 | Comparison of twinned $<110>_c$ zone axis patterns found at ambient and cryogenic temperature
Supplementary Fig. 20 | Additional spatial correlations between local photophysics (PL) and structure (SED) reveal bright isolated emission arising from a grain oriented close to the $<111>_c/<001>_h$ zone axis where nanotwins are oriented in the plane of the film.
Supplementary Fig. 21 | Kinematical simulations of diffraction patterns with increasing polytype order.
Supplementary Fig. 22 | Kinematically simulated patterns of the hexagonal polytypes of $FAPbI_3$ with increasing corner sharing layers viewed along $[001]_h$ direction (red) overlaid with the experimental $<111>_c$ $/<001>_h$ zone axis pattern (black) recorded at ambient temperature.
Supplementary Fig. 23 | An example workflow to quickly identify grains oriented near the $<111>_c/<001>_h$ zone axis shown for thermally evaporated $FAPbI_3$ on SiN_x grids.
Supplementary Fig. 24 | Overlay of '$<111>_c$' map and hyperspectral PL data (80K)
Supplementary Fig. 25 | Analysis of grains of $FAPbI_3$ oriented close to a $<110>$c zone axis, which are also in the vicinity of an above bandgap emitter show uniformity in the intensity of VDF images
Supplementary Fig. 26 | Grains of $FAPbI_3$ oriented close to a $<110>_c$ zone axis, which are not in the vicinity of an above bandgap emitter show a greater presence of striations when compared to the grains shown in Supplementary Fig. 25
Supplementary Fig. 27 | The overlaid SED and hyperspectral PL data taken at 706 nm emission for the region shown in main text Fig. 4
Supplementary Fig. 28 | The overlaid SED and hyperspectral PL data taken at 734 nm emission for the region shown in main text Fig. 4
Supplementary Fig. 29 | The electron beam damage of evaporated $FAPbI_3$ deposited on a SiN_x TEM grid. (a-d)
Supplementary Fig. 30 | To improve signal to noise ratio averaged diffraction patterns over single grains are presented instead of those from individual grains unless stated otherwise.
Supplementary Fig. 31 | The SLIC methodology applied to an SED scan.
Supplementary Fig. 32 | PCA clustering results
Supplementary Fig. 33 | Kinematically simulated zone axis patterns for $a^0a^0a^0$, $a^0a^0c^+$ and $a^+a^+a^+$
Supplementary Fig. 34 | Showing the appearance of superstructure peaks if oriented along $[123]_c$ zone axis
Supplementary Fig. 35 | Kinematically, simulated diffraction patterns of a 12H polytype with various tiling patterns imposed on the corner sharing layers
Supplementary Fig. 36 | Experimental patterns having undergone peak picking showing a lack of distortion upon cooling
Supplementary Fig. 37 | The appearance of superstructure reflection in hexagonal polytypes upon cooling
Supplementary Fig. 38 | Keypoints detected between two VBF images before being stitched together
Supplementary Fig. 39 | A cartoon schematic of how to spatially correlate between hyperspectral PL microscopy and the electron microscope.
Supplementary Fig. 40 | The optimum rotation between the two datasets found to be 62.72° where the NCC is maximal
Supplementary Fig. 41 | Finding the optimal translation between the hyperspectral PL and SED

Supplementary Fig. 42 | Overlay of hyperspectral PL and SED having used the AntsPy Python package
Supplementary Fig. 43 | The bands calculated from the KP model
Supplementary Fig. 44 | Comparison between the layer (L) progression across various polytypes and the *n*-index progression within a fixed polytype.
Supplementary Table 1 Simulated well and barrier lengths corresponding to each quantum peaks.

Supplementary Text

Supplementary Text 1: Acquisition and preprocessing of the SED data
Supplementary Text 2: Simple linear iterative clustering (SLIC)
Supplementary Text 3: Determination of the low temperature phase of $FAPbI_3$
Supplementary Text 4: Stitching of the SED data and spatial correlation with hyperspectral PL
Supplementary Text 5: Krönig-Penney superlattice
Supplementary Text 6: The mean lifetime of the excited carriers in the quantum levels

Other Supplementary Materials for this manuscript include the following:

Supplementary Movie 1: Wavelength dependent emergence of bright spots in the hyperspectral photoluminescence map

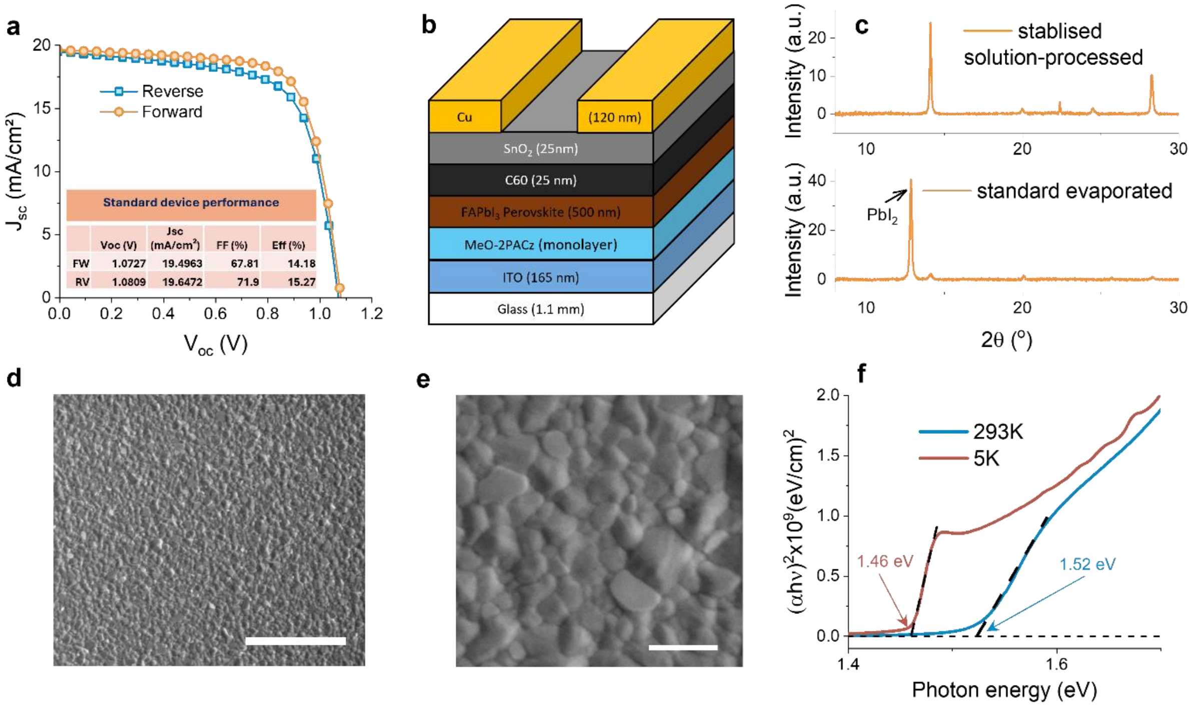

Supplementary Fig. 1 | Evaporated $FAPbI_3$ films exhibit good quality. (a) A representative J-V curve of the evaporated $FAPbI_3$ as a solar cell candidate. The inset provides details of the device performance, including forward and reverse scans. **(b)** Solar cell device structure. Commonly available transport and contact layers are utilized for the standard demonstration. **(c)** XRD patterns of both standard evaporated and stabilized solution-processed $FAPbI_3$ films. The signal of PbI_2 is from the slight extra proportion (20%) applied to stabilize the evaporated $FAPbI_3$ films.[1] In Fig. 1 and Supplementary Fig. 2, we find the quantum feature of $FAPbI_3$ films is not affected by such a slight PbI_2 addition. **(d)** and **(e)** display scanning electron microscopy micrographs of the evaporated $FAPbI_3$ films with scale bars of 4 µm and 500 nm, respectively. **(f)** The Tauc plot absorbance of $FAPbI_3$ films recorded at 293 K and 5 K, which show the position of the bandgap at 1.52 eV (817 nm) and 1.46 eV (849 nm), respectively. The continuous film of perovskite grains on the order of hundreds of nanometers in size can be also directly observed from the SED measurement, which can be seen in Fig. 3.

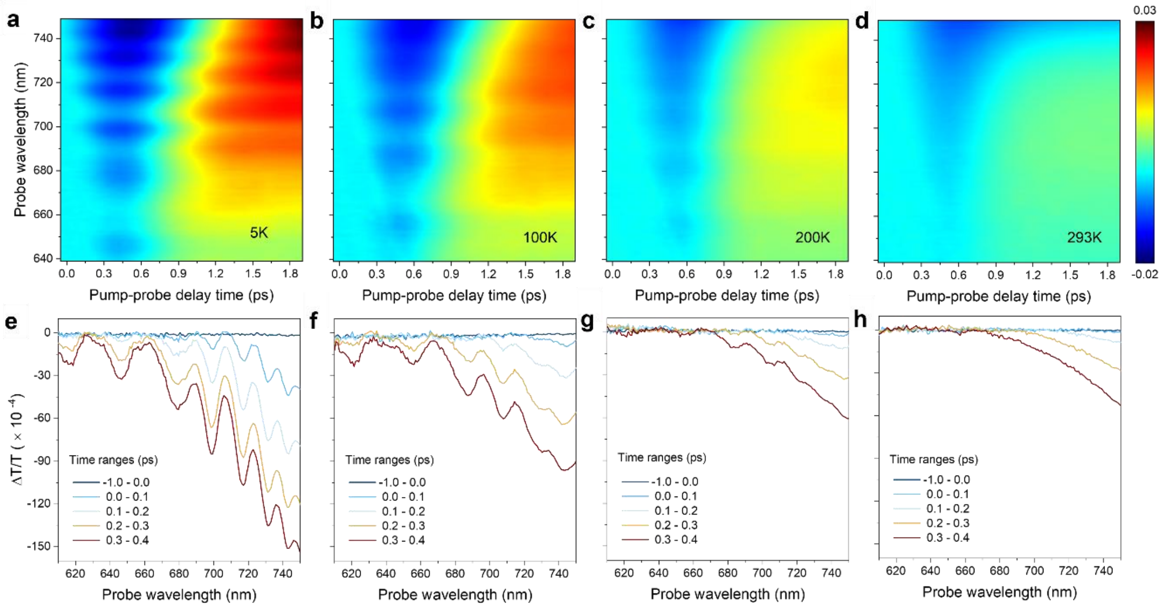

Supplementary Fig.2 | Temperature-dependent TA maps of solution-processed $FAPbI_3$. (a-d) Temperature-dependent TA maps of the stabilized solution-processed $FAPbI_3$ show that discrete absorption peaks fade out as the temperature increases, suggesting the quantum confinement is intrinsic and present regardless of the material deposition method. A different colour set is applied to distinguish the films from the two deposition methods. **(e-h)** Absorption spectra at initial time points, vertically extracted from the maps. While the signals in Figure 1 appear to oscillate around zero transmission, we observe that they do not strictly follow a symmetrical oscillation around this baseline. Instead, solution-processed films exhibit a sloping baseline, which we attribute to a bulk property characterized by the primary PL peak at 830 nm (measured at 80 K).

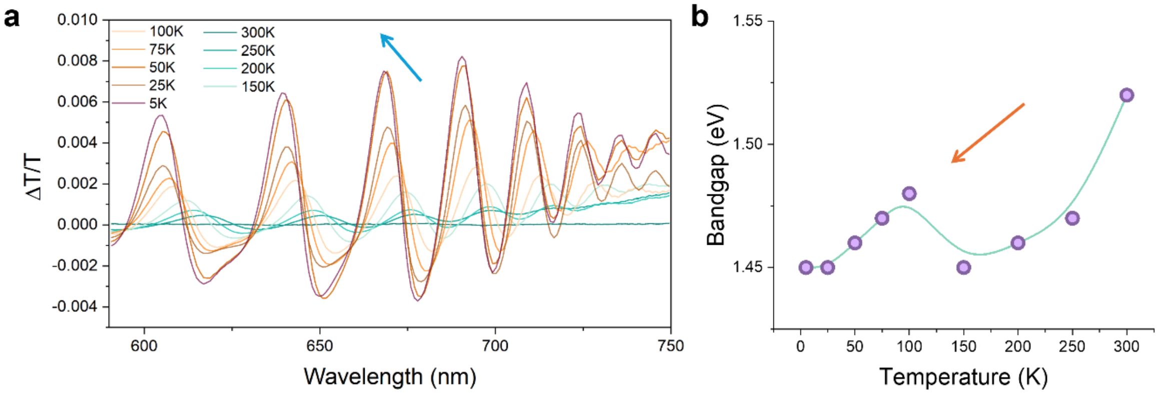

Supplementary Fig.3 | Temperature dependence of the quantum peaks and bandgap. (a) The blue shift in the quantum energy levels with temperature, which is independent of the shift in the bandgap (a two-step red shift breaks at phase transition temperature around 140 K) **(b)**. The bandgap values are obtained from the Tauc plot absorbance of $FAPbI_3$ films at each temperature, as exampled in Supplementary Fig. 1f.

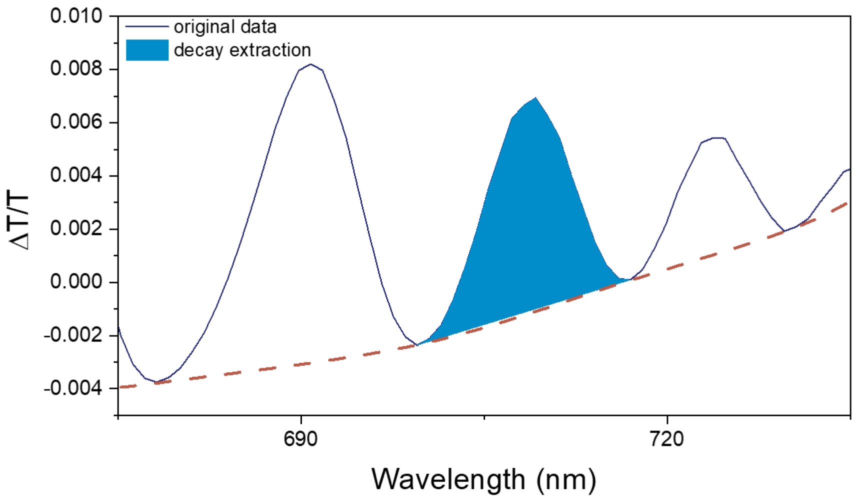

Supplementary Fig.4 | Extraction method for the transient peaks from TA data. By utilizing the smooth curve as the baseline, we extract the transient dynamics of the excited states at each quantum energy level, i.e. connecting the coloured area by time. The background arises from the difference in dielectric constant between the positions of the sample where the identical probe and reference beams are located, with only the former being overlapped by the pump.[2]

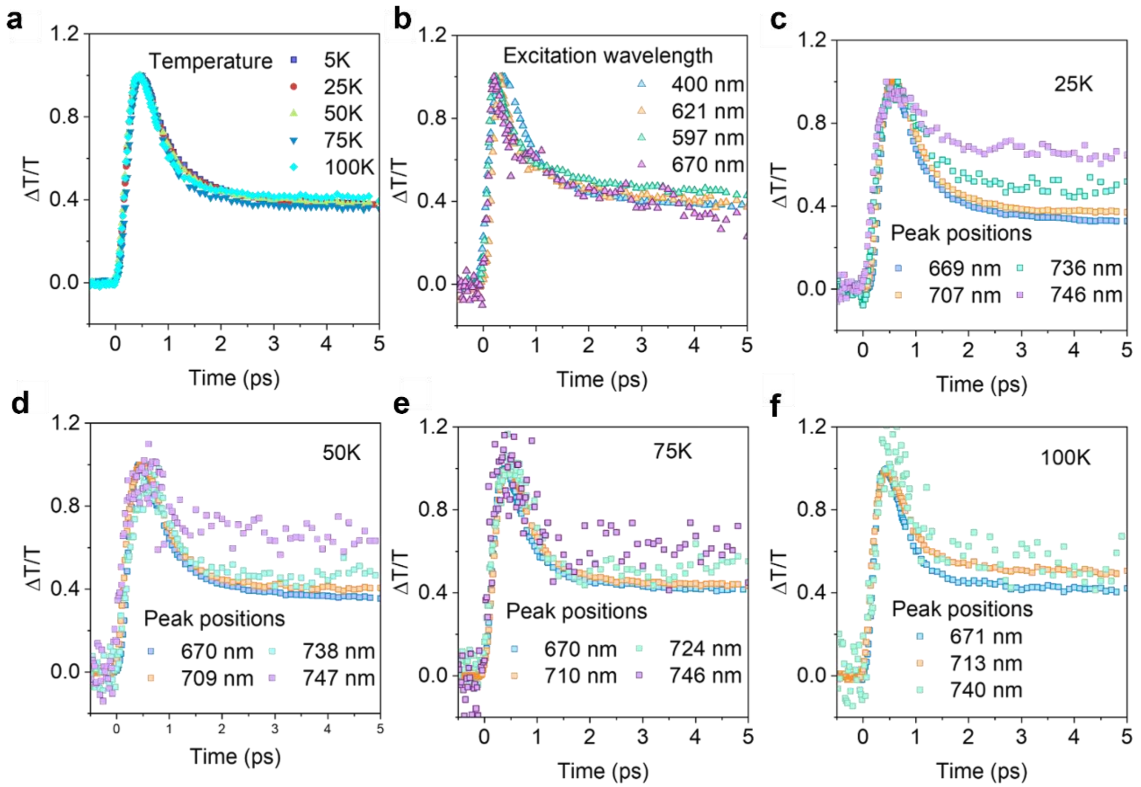

Supplementary Fig.5 | Temperature-dependent TA transients of evaporated $FAPbI_3$ thin film, complementary to Fig.1. (a) transients at 691 nm at different temperatures, complementary to Fig.1e with transients at 723 nm. **(b)** transients at 706 nm under different excitation wavelengths, complementary to Fig.1d with transients at 723 nm. **(c-f)** decay characteristics within each level under identical excitation wavelengths from 25 K to 100 K.

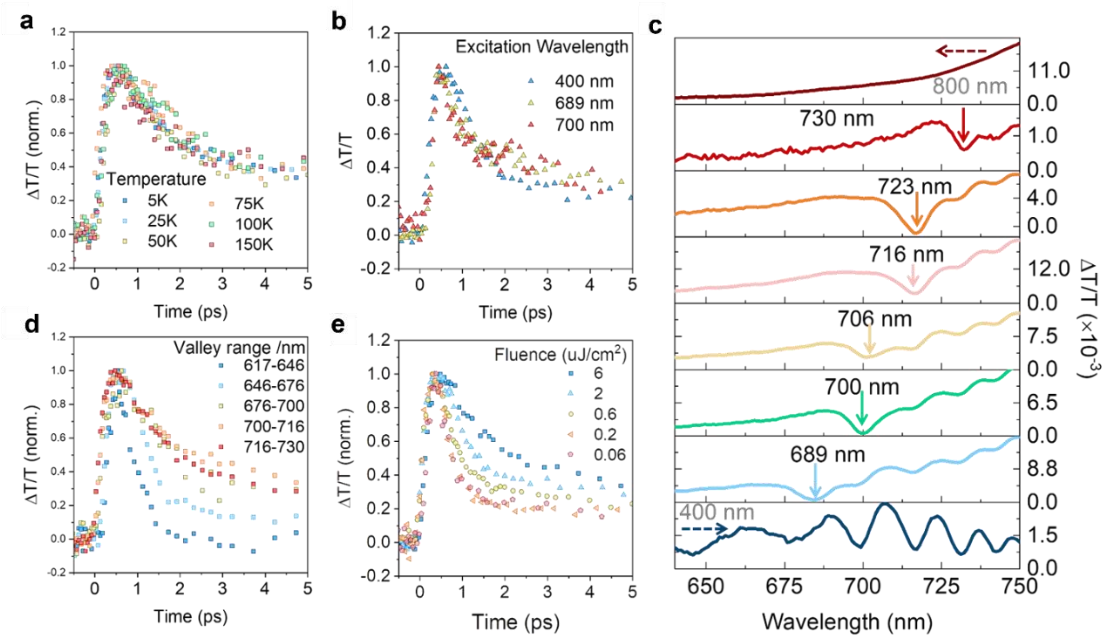

Supplementary Fig.6 | TA Recordings of the stabilized solution-processed $FAPbI_3$ thin film. Temperature-dependent **(a)** and excitation wavelength dependent **(b)** transients at peak position 723 nm. **(c)** Excitation wavelength dependent absorption spectra within a consistent time window. **(d)** Transients at different quantum peaks, excited by a fixed 400 nm laser beam with fluence 5.7 uJ/cm^2. **(e)** Power dependent transients at peak 723 nm, excited by laser at 400 nm.

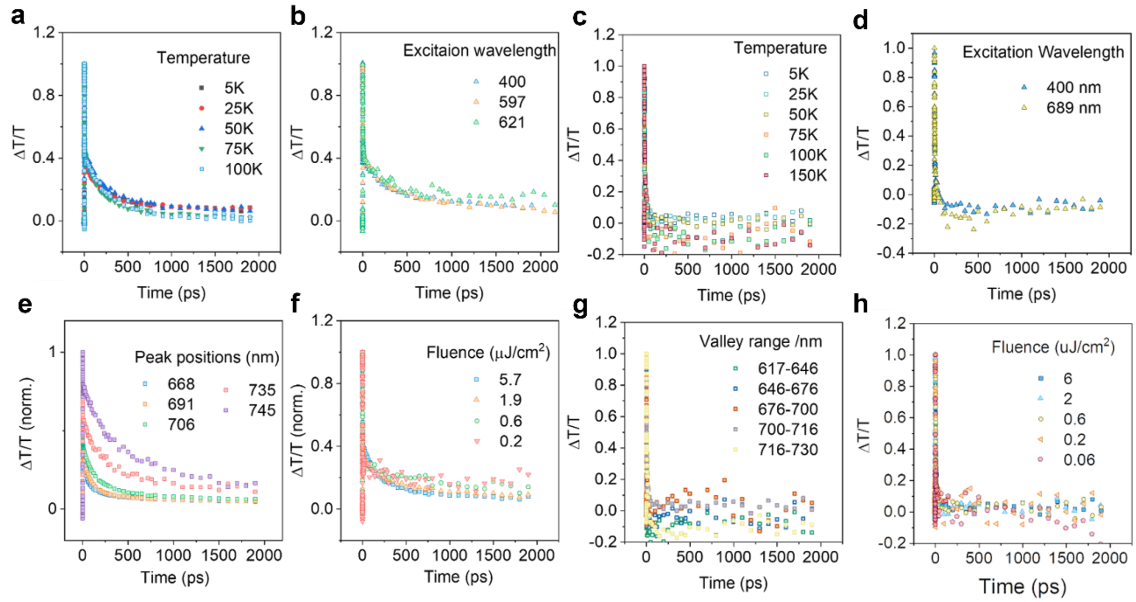

Supplementary Fig.7 | Full TA decays at the timescale of 2ns for the evaporated samples **(a-d)** and solution-processed samples **(e-h)**. Both samples show an ultrafast majority (1/e) decay, as clearly resolved in Fig. 1g with the timescales of 5 ps and 50 ps. Here, with the longer timescale of 2 ps, the evaporated samples exhibit a long-lasting signal pointing to 1 ns and marginally extending to 2 ns. In contrast, the solution-processed samples show no such tail at all, suggesting that the long signals are not intrinsic property of the material, and can be extrinsically controlled.

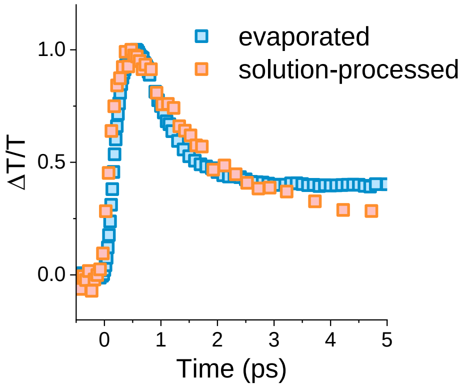

Supplementary Fig.8 | The TA transient comparison between the solution-processed and evaporated $FAPbI_3$ samples over the timescale of 5 ps.

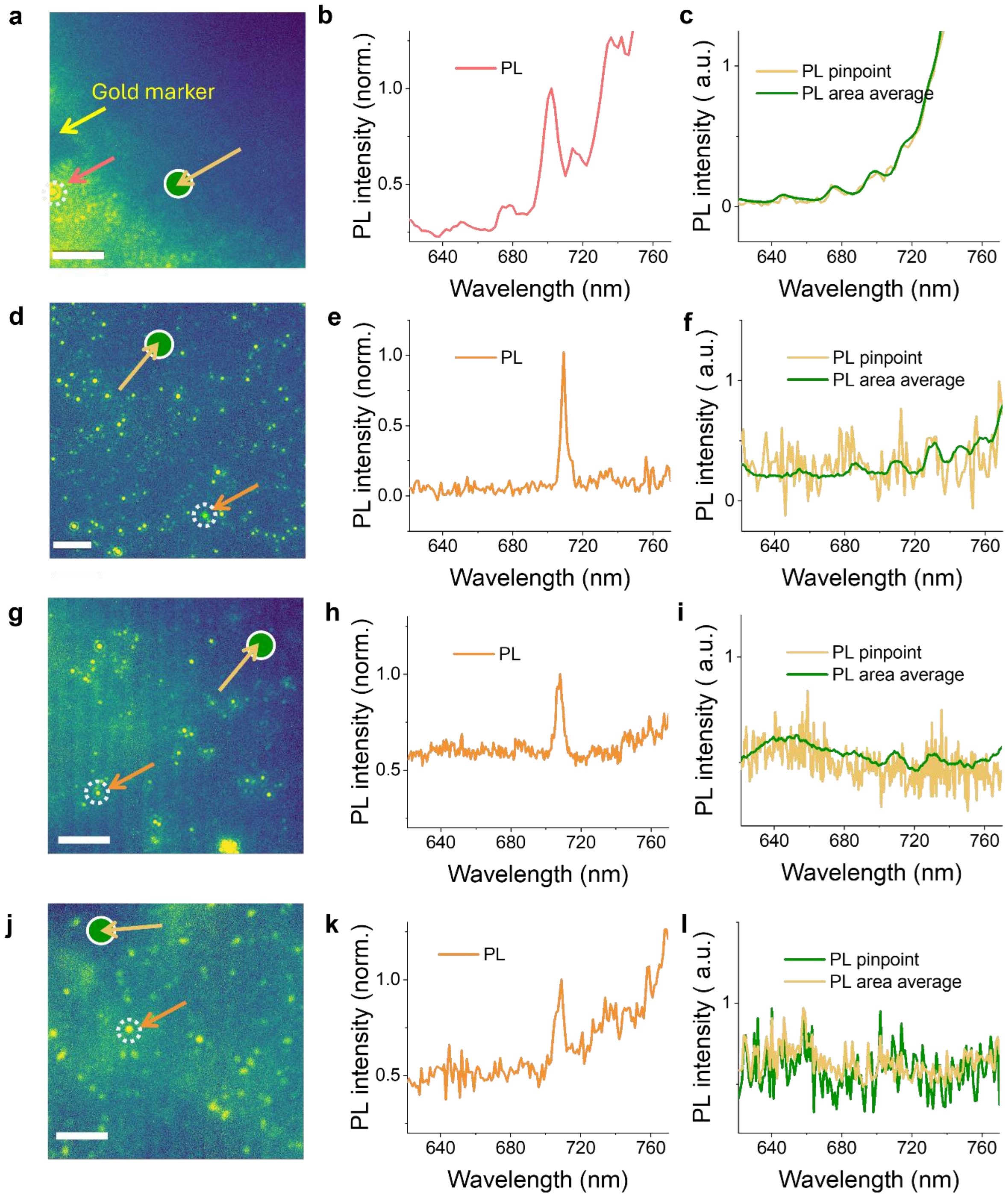

Supplementary Fig.9 | PL maps and local emission spectra of evaporated and solution-processed $FAPbI_3$ samples under different post-treatment conditions. Scale bar of (a,d,g,j): 10 μm. PL maps show spatial dependence of PL intensity at 706nm and are derived from hyperspectral cubes. The full PL spectra from local bright spots on the maps are shown. For reference, the background spectra are obtained by both pinpointing a single pixel and averaging over a circular area. **(a-c)**: Evaporated $FAPbI_3$ thin film. The pointed dark triangle is the gold marker, used as a locator for spatial correlation (**a**). The marked bright emitter shows an

outstanding high intensity PL peak at 706 nm along with other above bandgap peaks (**b**). The background PL peaks are also observed at dim areas in PL maps where no clear bright spots are observed (**c**). (**d-f**): solution-processed film without any additional treatment but aged in glovebox for one year. The background multi-peaks are in line with the PL recorded from evaporated samples and in accordance with the conclusion of ubiquitous spread of nanotwinning domains from the structural analysis in section 3 of the main text. (**g-i**) fresh solution-processed film without any additional treatment. In this case, clear but less background peaks are observed. (**j-l**): solution-processed film with an additional annealing treatment (150°C in air for 30 minutes) and aged in glovebox for one year. This sample, after post-treatment (**l**), does not show clear background multi-peaks, meaning much less overlapping or pile-up of different single emission peaks. The suppressed appearance of background peaks suggests that the overall spread distribution of single peaks can be modulated by post-treatment. Such suppression lasts after aging implies that the annealing post-treatment does not only improve PL of the whole film as we previously reported[14] but also has a long-term structural impact. Despite the suppression of background peaks, the single emission peak could still be found (**k**), suggesting an intrinsic formation of the local nanoscale structural source within the $FAPbI_3$ samples.

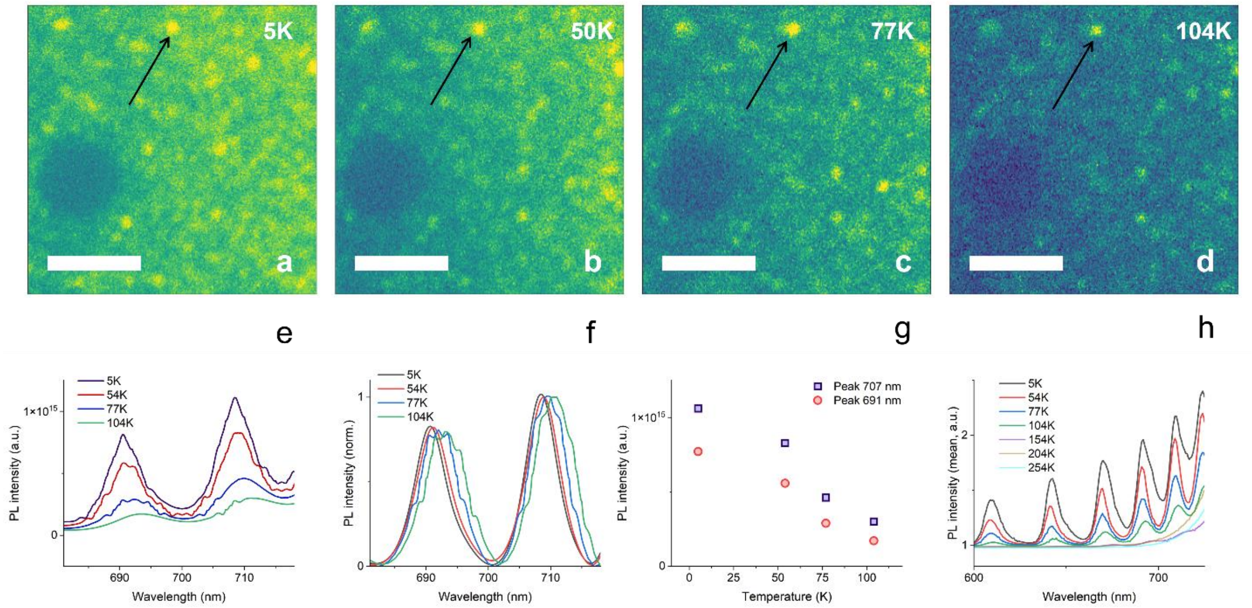

Supplementary Fig. 10 | PL maps above the bandgap energy taken at different temperatures of a $FAPbI_3$ film deposited on a SiNx TEM grid. The data are drift corrected to overlay with the maps taken at 5 K, and emission at 689 nm is shown as an example (a-d). Scale bar: 10 µm. The arrow-pointed emitter indicates that, although the emitters decrease in intensity with increasing temperature, their position remains fixed. The original (e) and spline-based subtracted, normalized PL spectra (f) are shown at different temperatures. While the PL intensity decreases linearly with increasing temperature (g), the emission wavelength shows a slight gradual shift with temperature, consistent with the shift observed in absorption results (Supplementary Fig. 3). Negligible changes to PL spread or FWHM are observed, suggesting that the structural study conducted at 80 K is not affected by temperature discrepancies. (h) A broader range of peaks and temperatures than (e) within the same region. The spectra are spatial average of the region. The quantum transients and spectra are maintained well below and above the nominal bulk phase transition at 140 K: at 154 K, two dim peaks (698 nm and 706 nm) can still be observed, consistent with TA data. This indicates the quantum peaks are not influenced by the bulk phase transition.

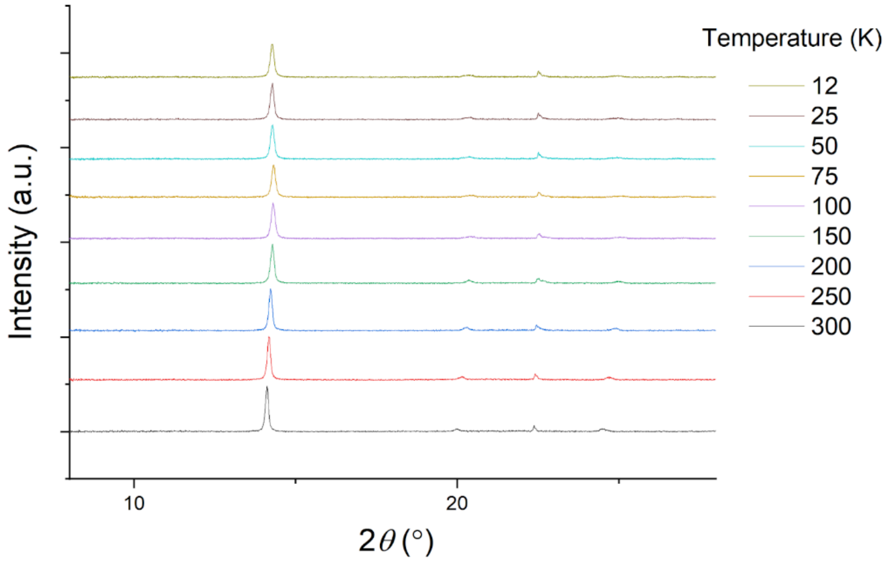

Supplementary Fig. 11 | Temperature dependent X-ray diffraction of the solution-processed $FAPbI_3$ thin film. The PbI_2 which peaks at 12.8 is not observed through all temperatures.

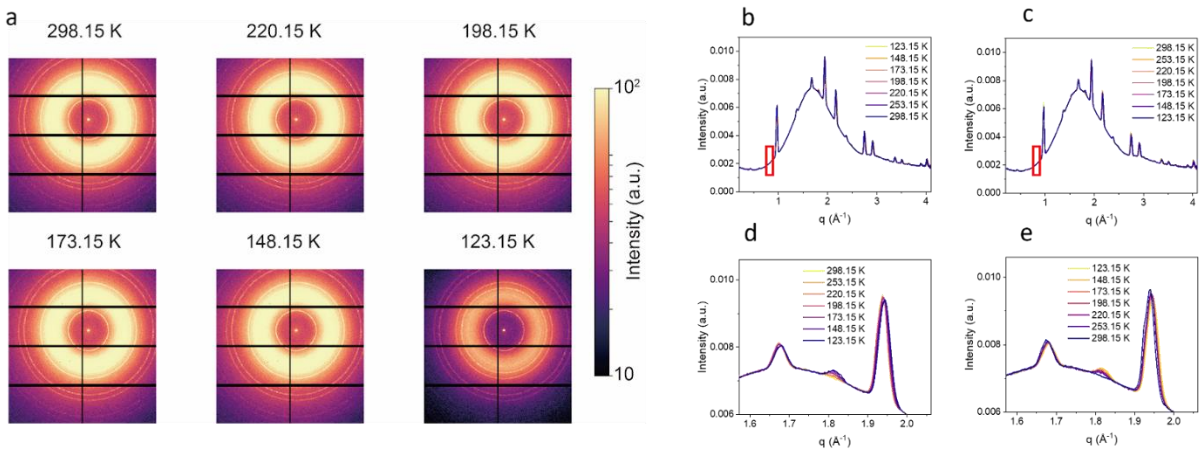

Supplementary Fig. 12 | Temperature-dependent wide-angle X-ray scattering (cryo-WAXS) of a solution-processed $FAPbI_3$ thin films. The PbI_2 peak is located at 0.89 Å^{-1}, and there is no such peak as shown in **b** and **c**. This is further confirmation of non-existent PbI_2 in our fabricated materials at any measured temperature. Therefore, the excess PbI_2 in the evaporated $FAPbI_3$ is not related to the quantum signals.

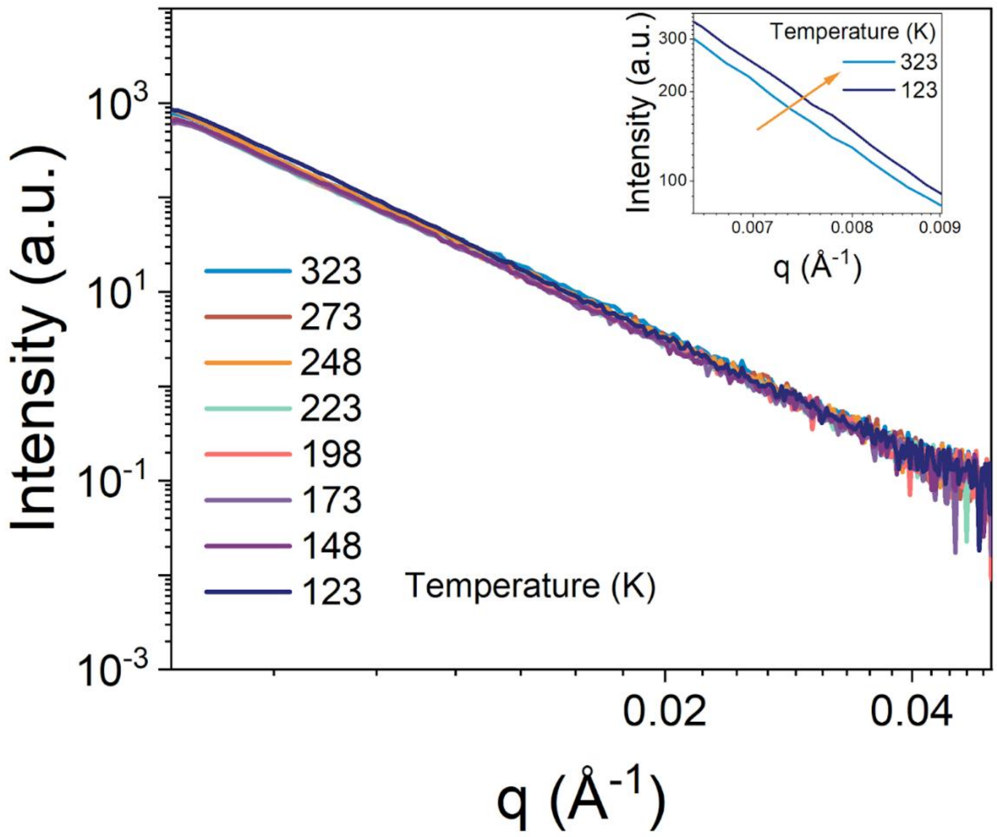

Supplementary Fig. 13 | Temperature dependent small-angle X-ray scattering (SAXS) results reveal structure information on the evolution of the structure from 1 to 50 nanometers. The results show that during the cooling process of the solution-processed $FAPbI_3$ sample, the entire sample exhibits a dense bulk-like structure (low q scattering following the power law $I = q^{-4}$). Slight roughness interface appears from 323K due to the shrinkage of the film (Supplementary Fig.11 inset).

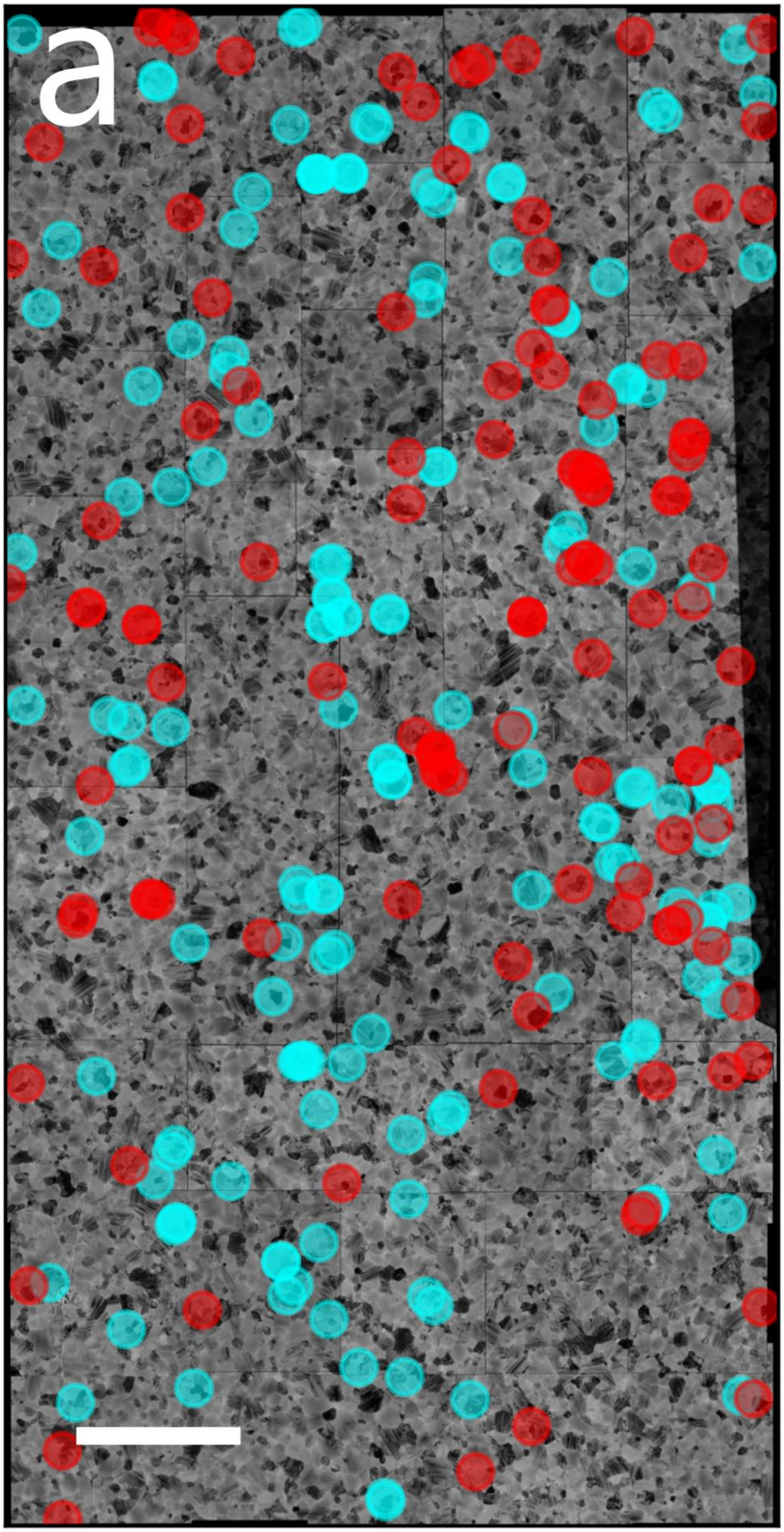
a

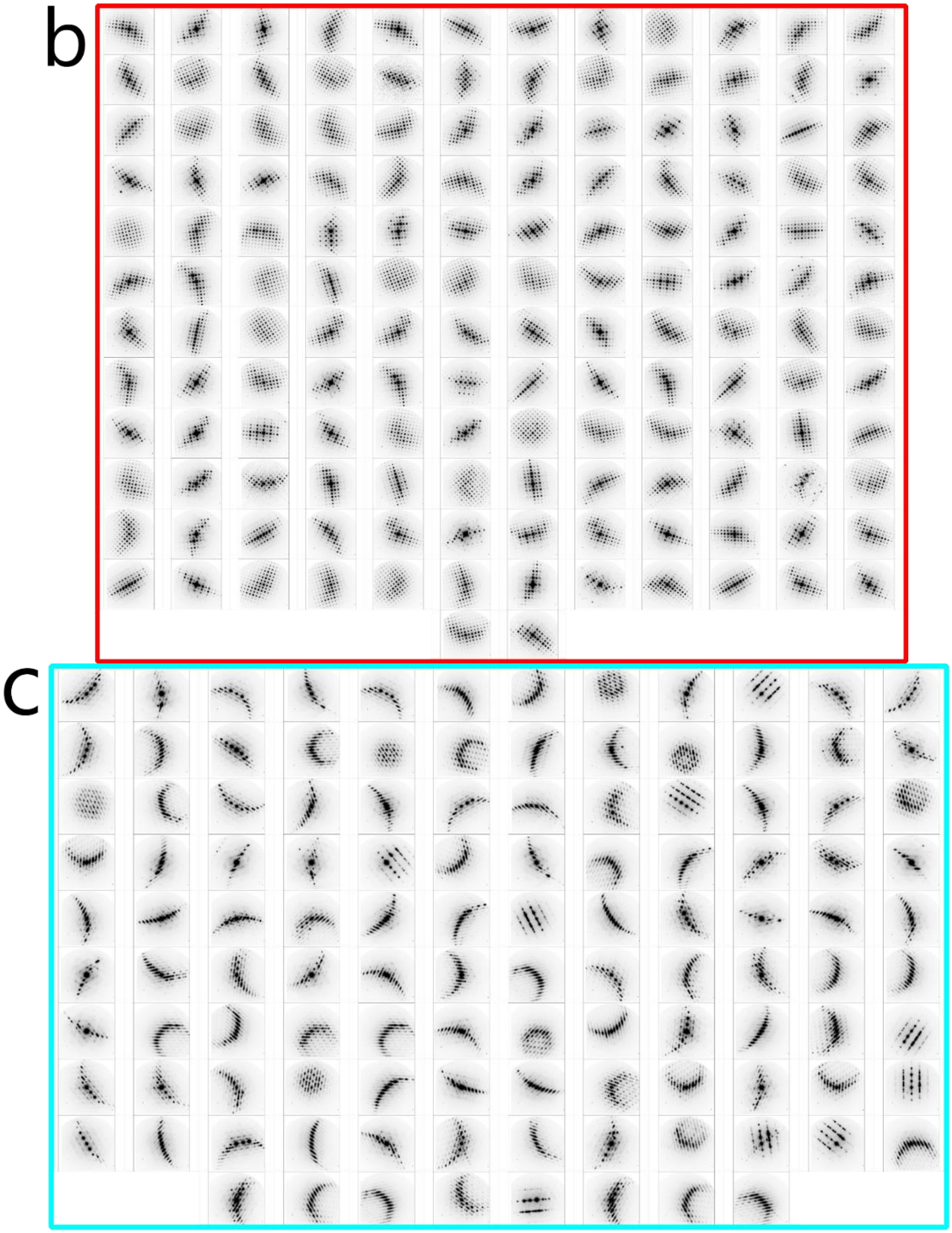

Supplementary Fig. 14 | Stitched SED data recorded at ambient temperature. (a) The stitched SED data of thermally evaporated $FAPbI_3$ on a SiN_x membrane at ambient temperature. **(b)** Patterns which are oriented close to a <001>$_c$ zone axis are marked in red. **(c)** Patterns which show characteristic $\{111\}_c$ twinning are marked in cyan. Scale bar: 3µm.

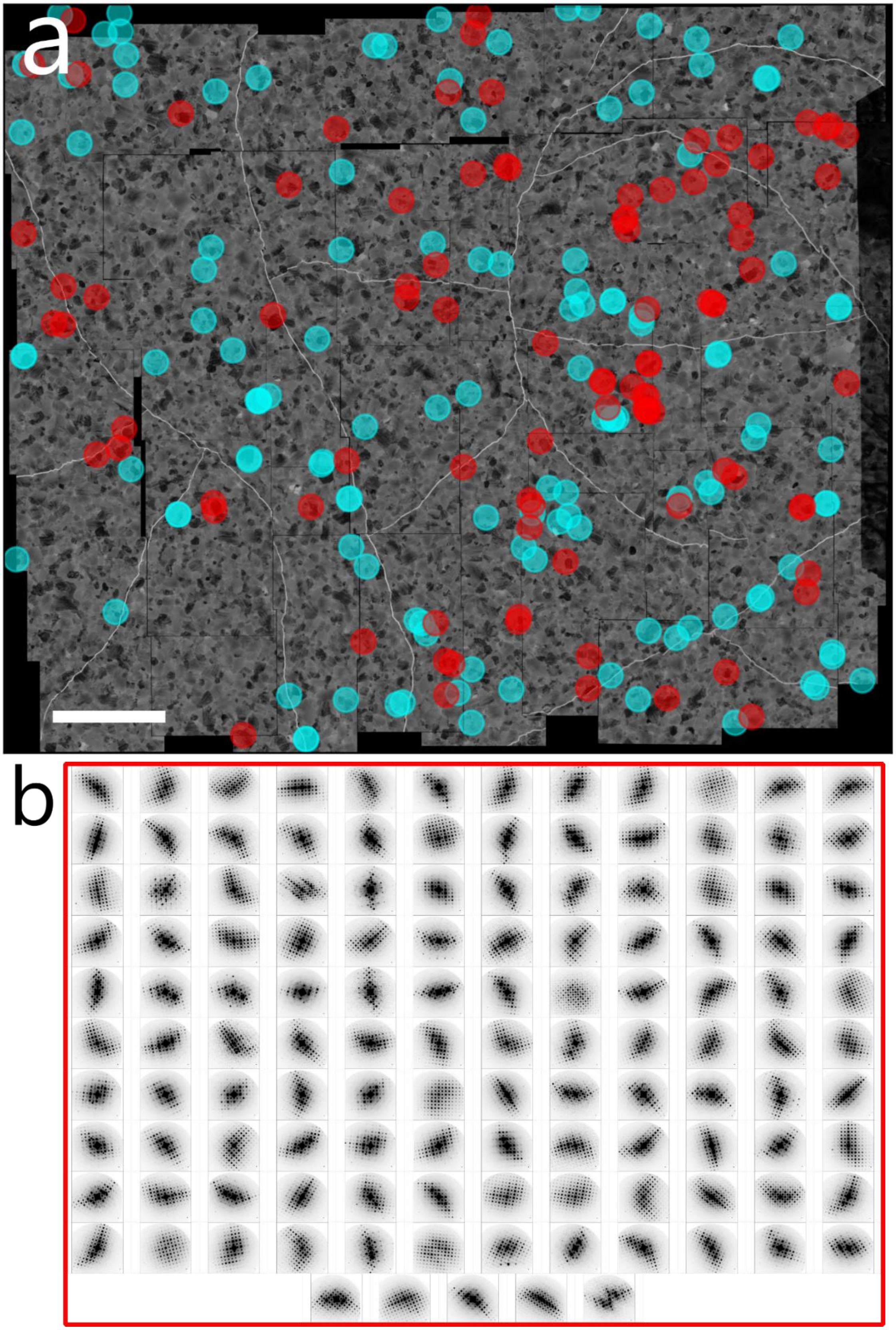
a
b

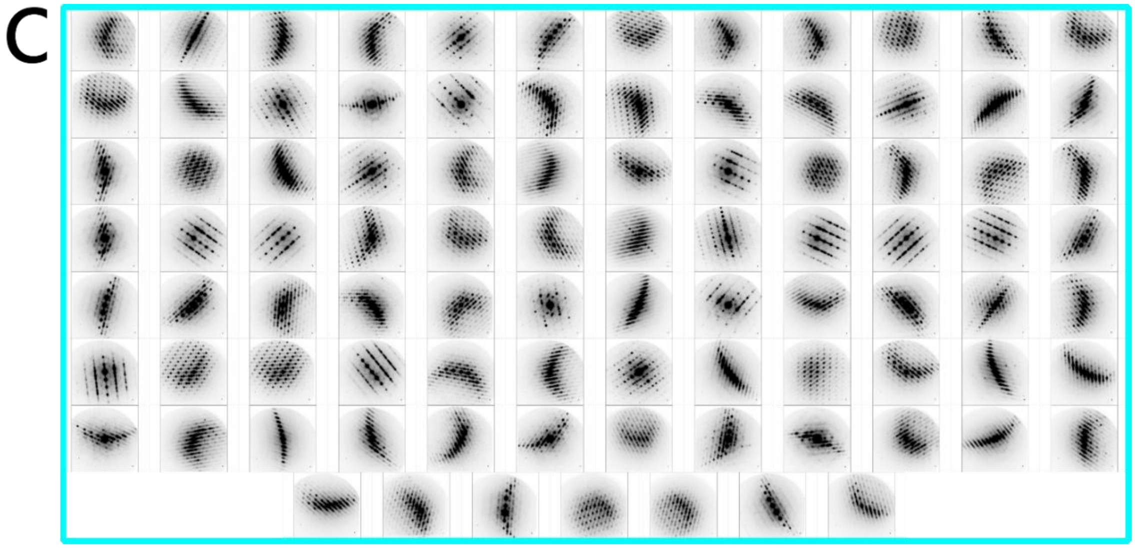

Supplementary Fig. 15 | Stitched SED data recorded at ~90K.(a) The stitched SED data of thermally evaporated $FAPbI_3$ on a SiN_x membrane at ~90K. Note the stitching is not as robust as the ambient equivalent due to the slight drift experienced at cryogenic temperatures **(b)** Patterns which are oriented close to a <001>$_c$ zone axis are marked in red. **(c)** Patterns which show characteristic $\{111\}_c$ twinning are marked in cyan. Scale bar: 3μm.

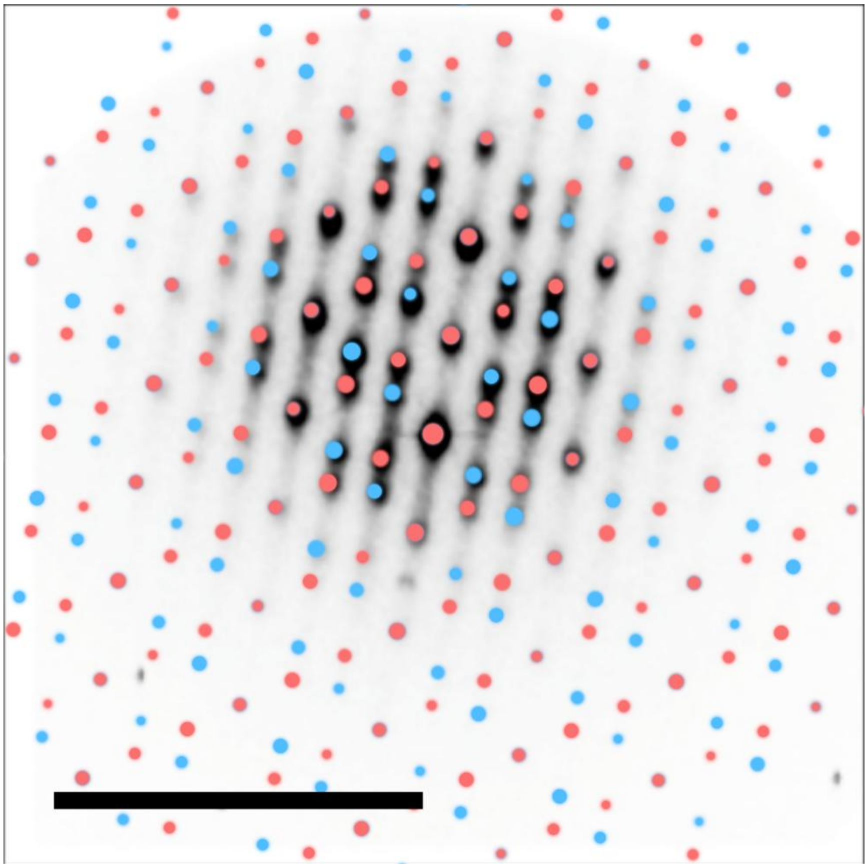

Supplementary Fig. 16 | A kinematical simulation of two $[110]_c$ zone axis patterns rotated such that the $(\bar{1}11)_c$ and $(1\bar{1}1)_c$ reflections are shared and overlaid onto an experimental pattern taken at ambient temperature *.cif* files for the simulation were based on crystallographic data.[3] Scale bar is $1Å^{-1}$.

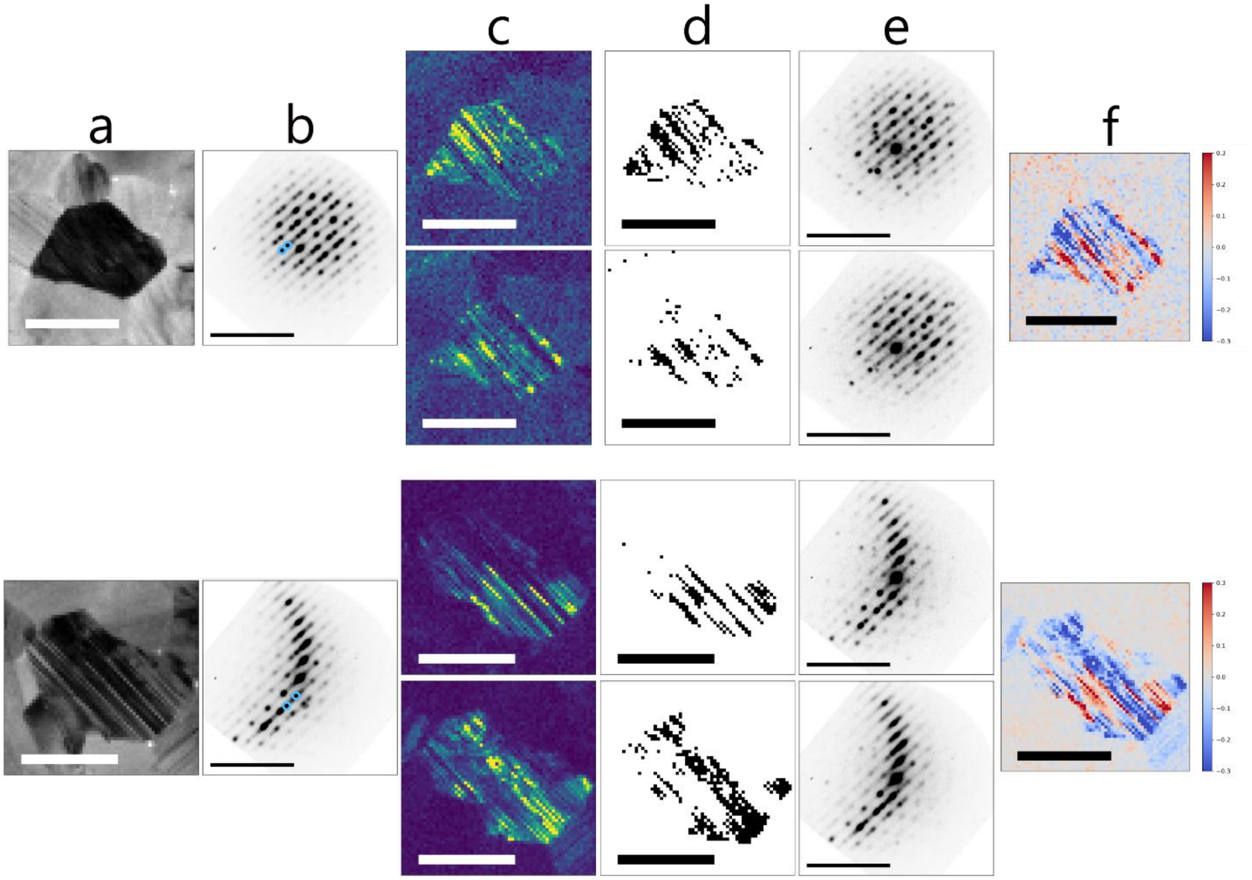

Supplementary Fig. 17 | SED data of thermally evaporated $FAPbI_3$ on SiNx grids recorded at ambient temperature. **(a)** Virtual bright field images of grains oriented near the $\{110\}_c$ zone axis recorded at ambient temperature. **(b)** Average diffraction patterns extracted from the grain marked in (a). **(c)** VDF images formed by placing an aperture over Bragg spots of complementary twins. **(d)** Binary masks formed from the virtual darkfield images in (c). **(e)** Diffraction patterns extracted when the binary masks are applied to the SED data in (a). Importantly it is observed that each of these patterns is still indicative of nanotwinning and that there is not a great change in the position of the Ewald sphere or Laue circle between the images. **(f)** Image formed when the two complementary VDF images are subtracted from each other showing the contrast approximately anti-correlates. Scale bars a,c,d&f: 200 nm; b&e: 1Å^{-1}.

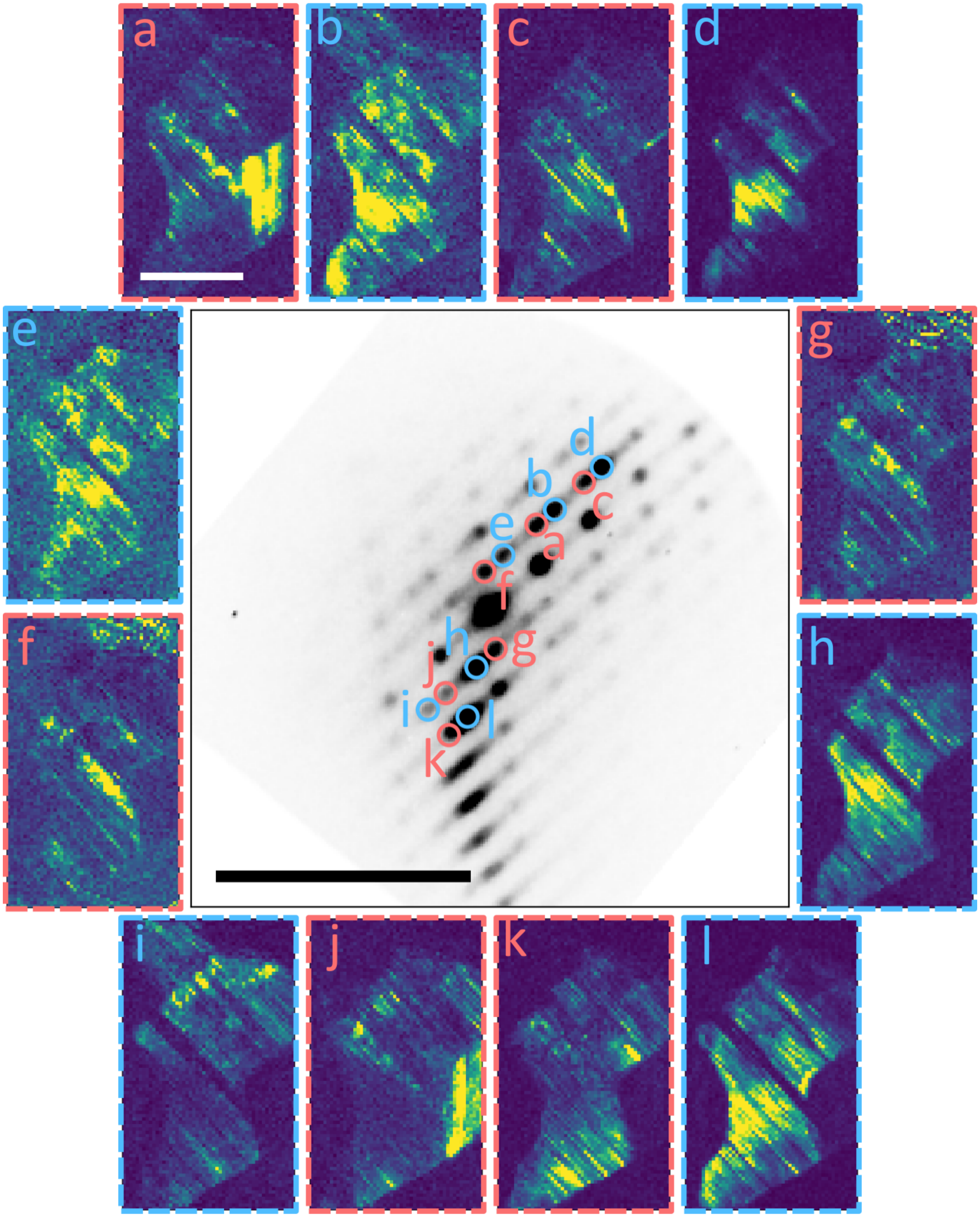

Supplementary Fig. 18 | Demonstration that similar anticorrelating contrast can be formed from many sets of complementary spots. We note that differences are observed from slight bending, twisting and microstrain being present within the grain and that the most prominent anticorrelating contrast is observed from spots close to each other in the diffraction pattern are chosen to form a VDF because of this. Scale bars: 300 nm and 1Å^{-1}.

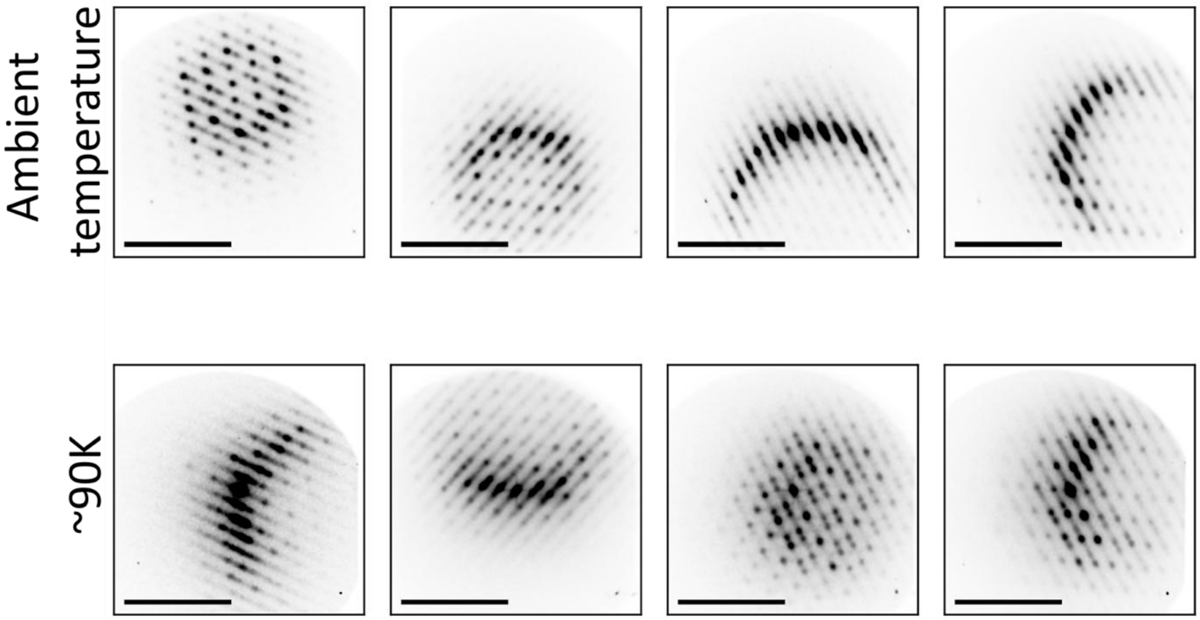

Supplementary Fig. 19 | Comparison of twinned <110>$_c$ zone axis patterns found at ambient and cryogenic temperature. Averaged diffraction patterns from SED of thermally evaporated $FAPbI_3$ on a SiN_x membrane showing $\{111\}_c$ type nanotwinning between ambient and cryogenic temperatures with patterns from the same grain shown in each column. No great change is observed in the nanotwinning between ambient and cryogenic temperatures but a small change in orientation is often observed upon cooling. Scale bars 1Å^{-1}.

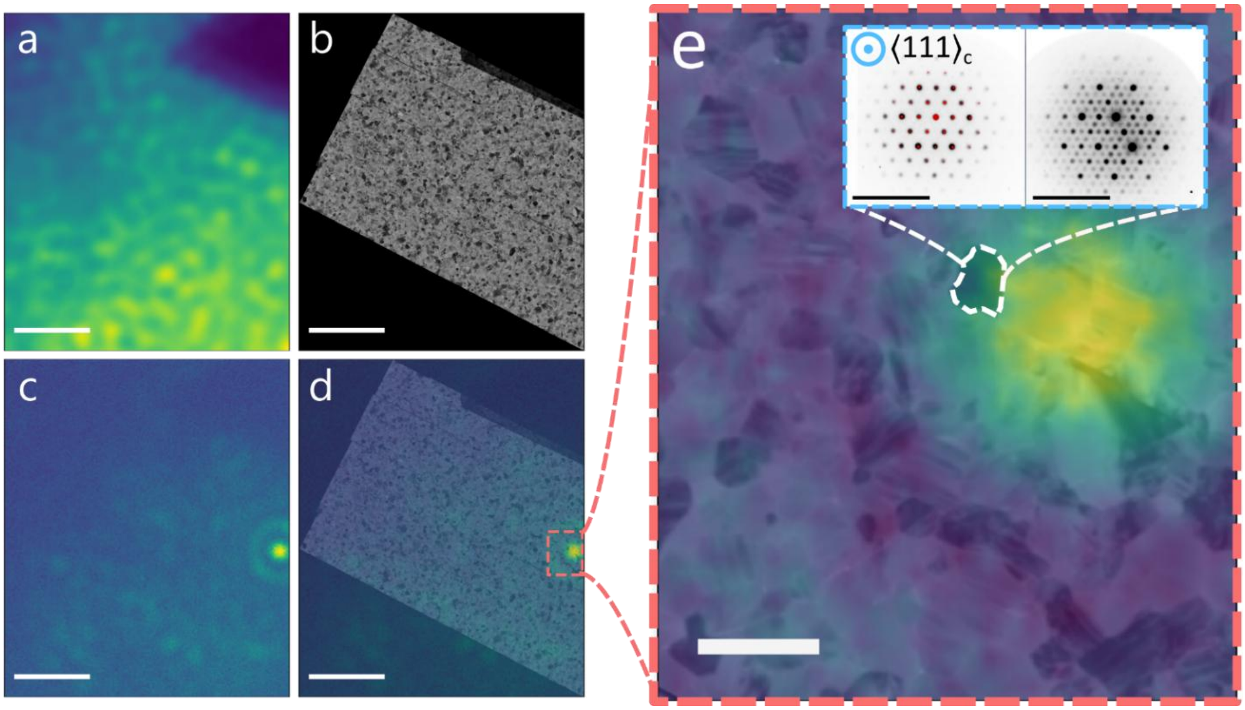

Supplementary Fig. 20 | Additional spatial correlations between local photophysics (PL) and structure (SED) reveal bright isolated emission arising from a grain oriented close to the <111>$_c$/<001>$_h$ zone axis where nanotwins are oriented in the plane of the film. (**a**) Hyperspectral data summed over all wavelengths taken at 80K. (**b**) Stitched SED (ambient temperature) once correlated to the hyperspectral PL (80K) via the use of a Au fiducial marker. (**c**) The frame at 706 nm in the hyperspectral data. (**d**) Overlaid SED and hyperspectral PL data. (**e**) Zoom-in of the panel marked in (d), inset showing the diffraction pattern from the grain marked at both ambient and cryogenic temperatures (~90K) respectively (overlaid red pattern shows kinematical simulation of diffraction from cubic $FAPbI_3$ oriented along the <111>$_c$ zone axis.[4]

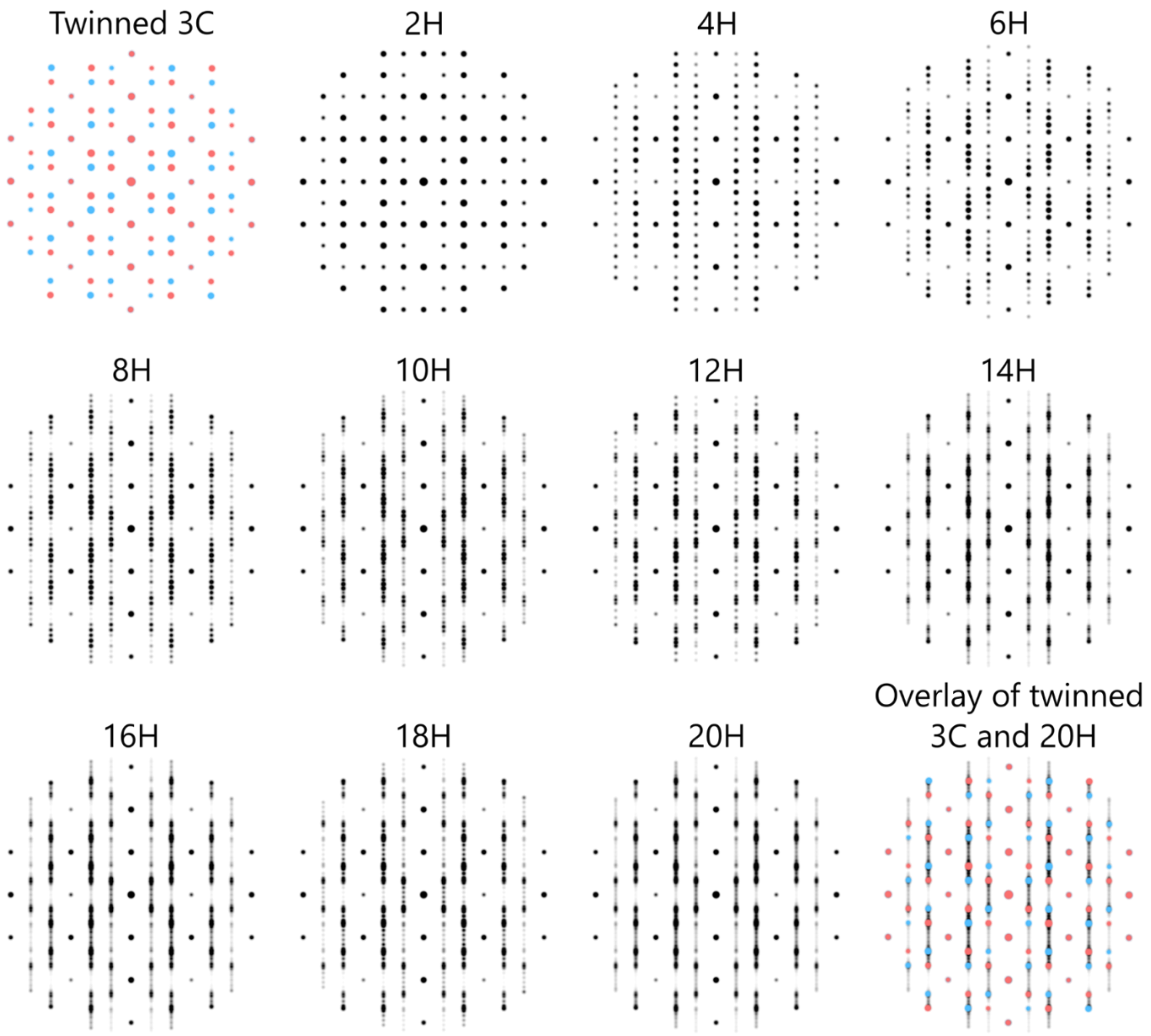

Supplementary Fig. 21 | Kinematical simulations of diffraction patterns with increasing polytype order. Kinematical simulations showing that when the order of the hexagonal polytype increases the $[100]_h$ zone axis pattern resembles the twinned 3C phase with streaking along the $<111>_c$ direction. .*cif* files were created similarly to.[5,6]

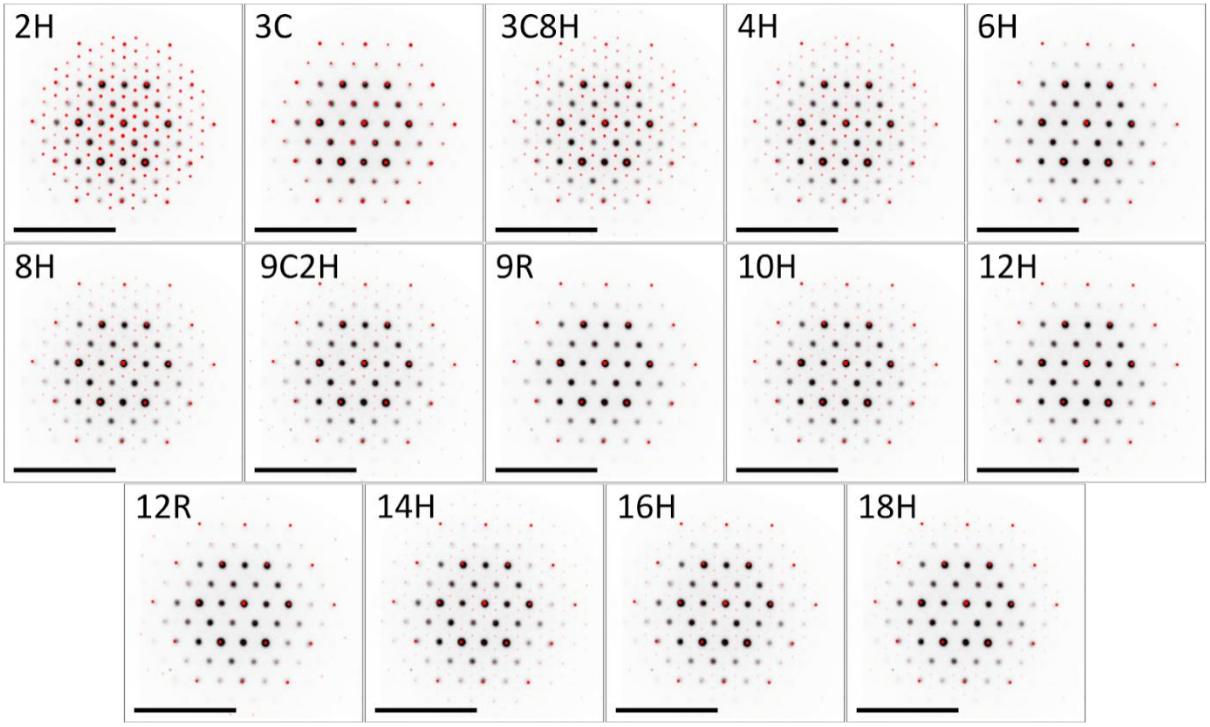

Supplementary Fig. 22 | Kinematically simulated patterns of the hexagonal polytypes of $FAPbI_3$ with increasing corner sharing layers viewed along $[001]_h$ direction (red) overlaid with the experimental $<111>_c$ /$<001>_h$ zone axis pattern (black) recorded at ambient temperature. Notice that extra reflections are absent for the 3C, 6H, 9R, 12H and 12R polytype. All scale bars are $1Å^{-1}$.

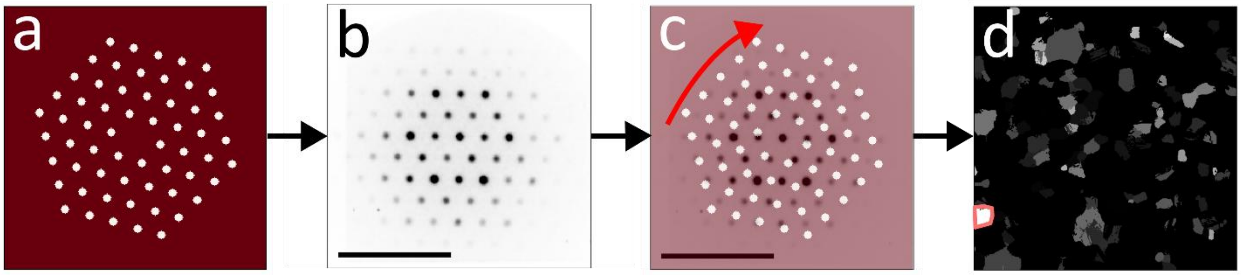

Supplementary Fig. 23 | An example workflow to quickly identify grains oriented near the <111>$_c$/<001>$_h$ zone axis shown for thermally evaporated $FAPbI_3$ on SiN_x grids. **(a)** A mask created from a simulation of a <111>$_c$ zone axis pattern. **(b)** Experimental pattern found via the clustering outlined in Supplementary Note 2. **(c)** The mask is overlaid on the experimental patterns and rotated 360°; a VDF image is then formed where the intensity is maximal throughout this rotation. **(d)** Image found where intensity is given by the maximum from step **(c)** for each cluster. Notice that the bright grain marked (pink) is oriented along the <111>$_c$ direction as this matches well with the mask. Scale bars 1Å^{-1}.

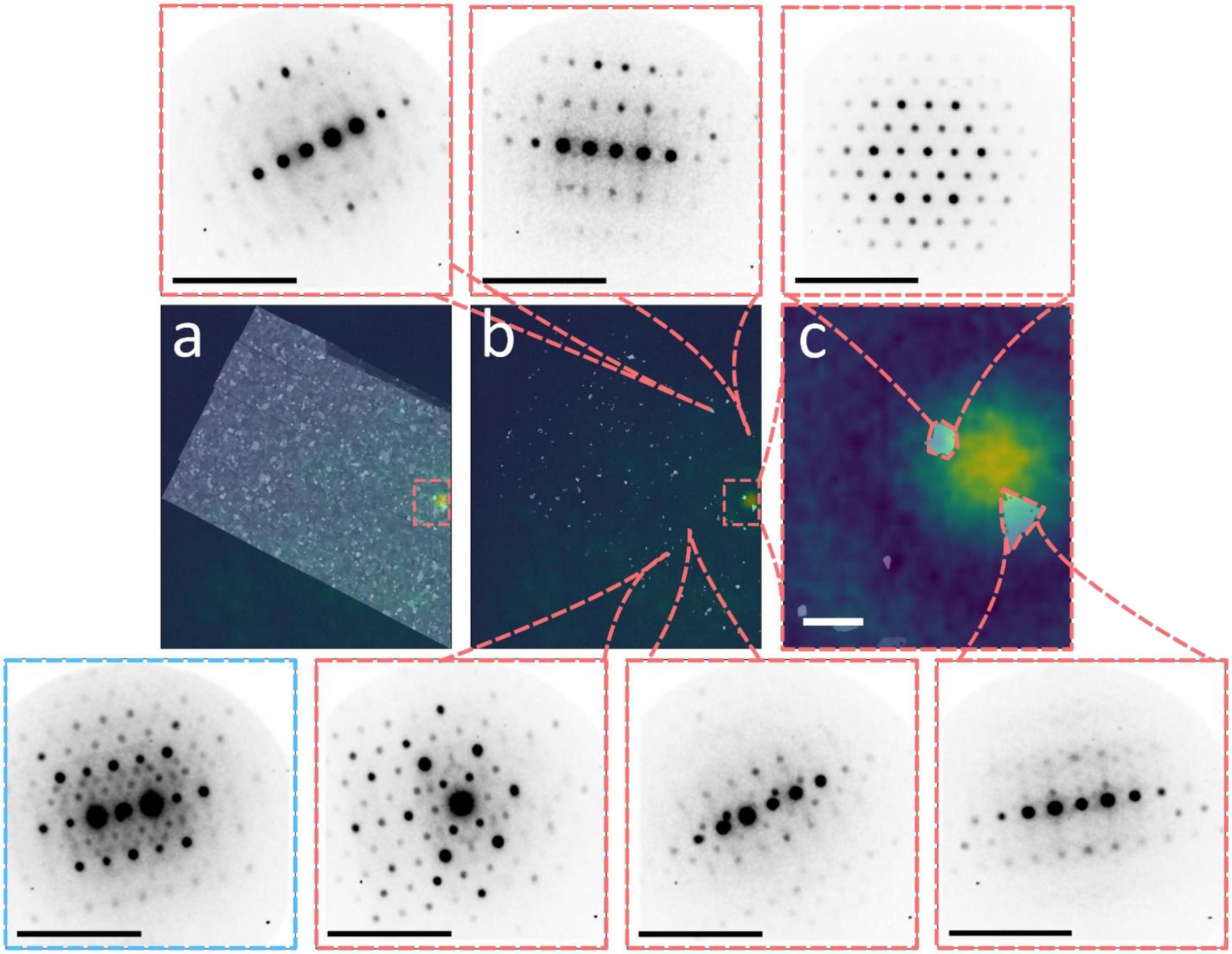

Supplementary Fig. 24 | Overlay of ‘<111>$_c$ map’ and hyperspectral PL data (80K) (a) The ‘<111>$_c$ zone axis map’ for thermally evaporated $FAPbI_3$ deposited on SiN_x grids, constructed using the method described in Supplementary Fig. 21 overlaid with the hyperspectral PL data showing the isolated emitter (80K). **(b)** The overlay shown in (a) with a threshold so only the most intense features in the ‘<111>$_c$ zone axis map’ are shown. **(c)** Expansion from the area marked in (b). Surrounding diffraction patterns which show the <111>$_c$ zone axis pattern discussed is a distinct structural feature, especially in the vicinity of the isolated emitter, with very few grains being observed oriented along this zone axis and many of the other bright grains being false positives. An instance of another grain oriented close to the <111>$_c$ zone axis is observed in this sample with superstructure reflections appearing at low temperature (shown in blue and discussed in Supplementary note 3). Scale bars: central row: 500 nm and top/bottom row: 1Å^{-1}.

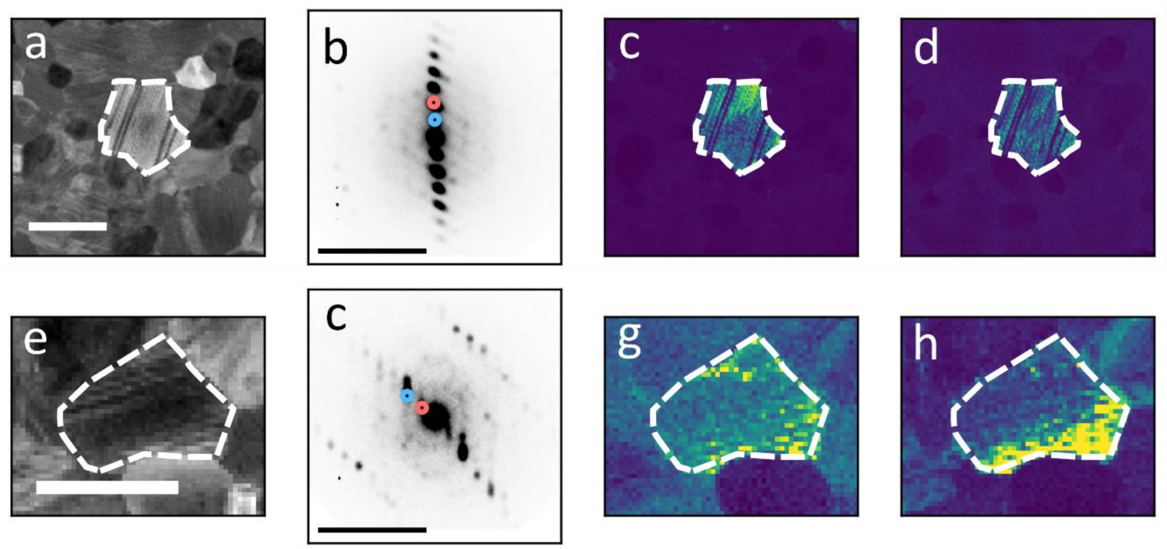

Supplementary Fig. 25 | Analysis of grains of $FAPbI_3$ oriented close to a <110>$_c$ zone axis, which are also in the vicinity of an above bandgap emitter show uniformity in the intensity of VDF images, shown for thermally evaporated $FAPbI_3$ on SiN_x grids. (a&e) VDF images formed from taking diffracted intensity between 0.1 and 1Å^{-1}. (b&c) The mean diffraction pattern from the regions marked in (a) and (e). (c,d,g,h) Virtual darkfield images when apertures are placed over the spots indicated in (b) and (c). Scale bars are (a): 300 nm; (e): 200 nm; (b&c): 1Å^{-1}.

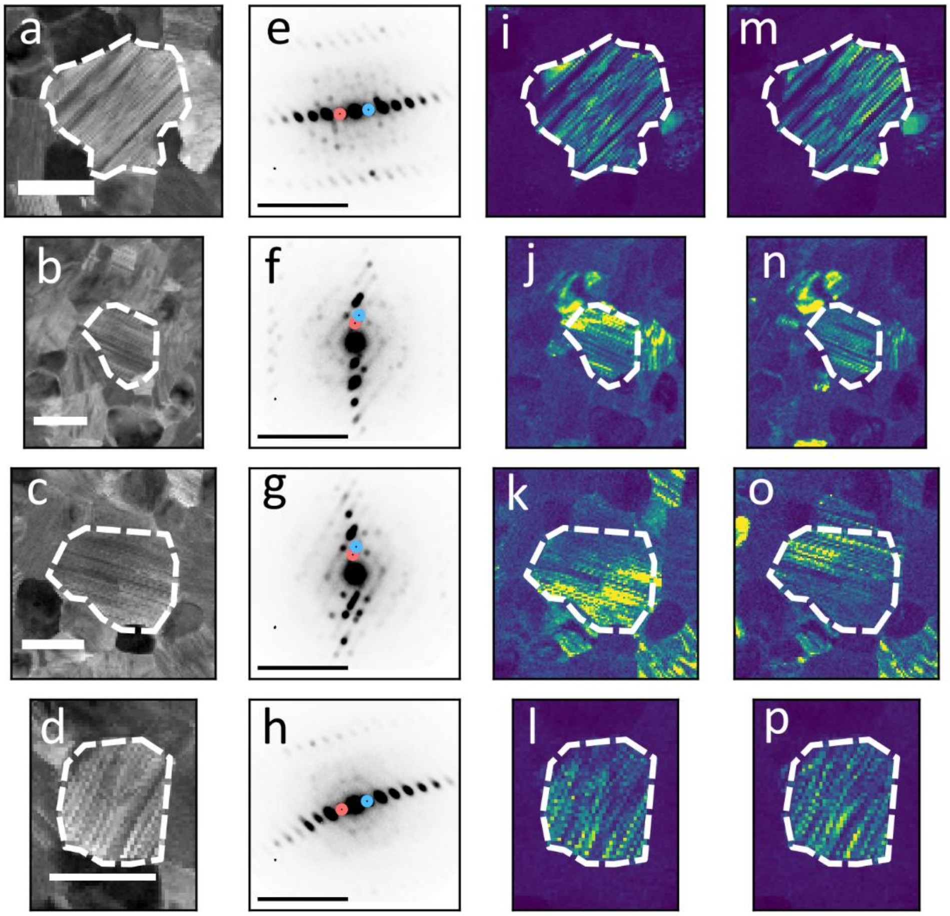

Supplementary Fig. 26 | Grains of $FAPbI_3$ oriented close to a <110>$_c$ zone axis, which are not in the vicinity of an above bandgap emitter show a greater presence of striations when compared to the grains shown in Supplementary Fig. 25, shown for thermally evaporated $FAPbI_3$ on SiN_x grids. (a-d) VDF images formed from taking diffracted intensity between 0.1 and 1Å^{-1}. (e-h) The mean diffraction pattern from the regions marked in (a-d). (i-p) Virtual darkfield images when apertures are placed over the spots indicated in (e-h). Scale bars are (a-d): 200 nm; (e-h): 1 Å^{-1}.

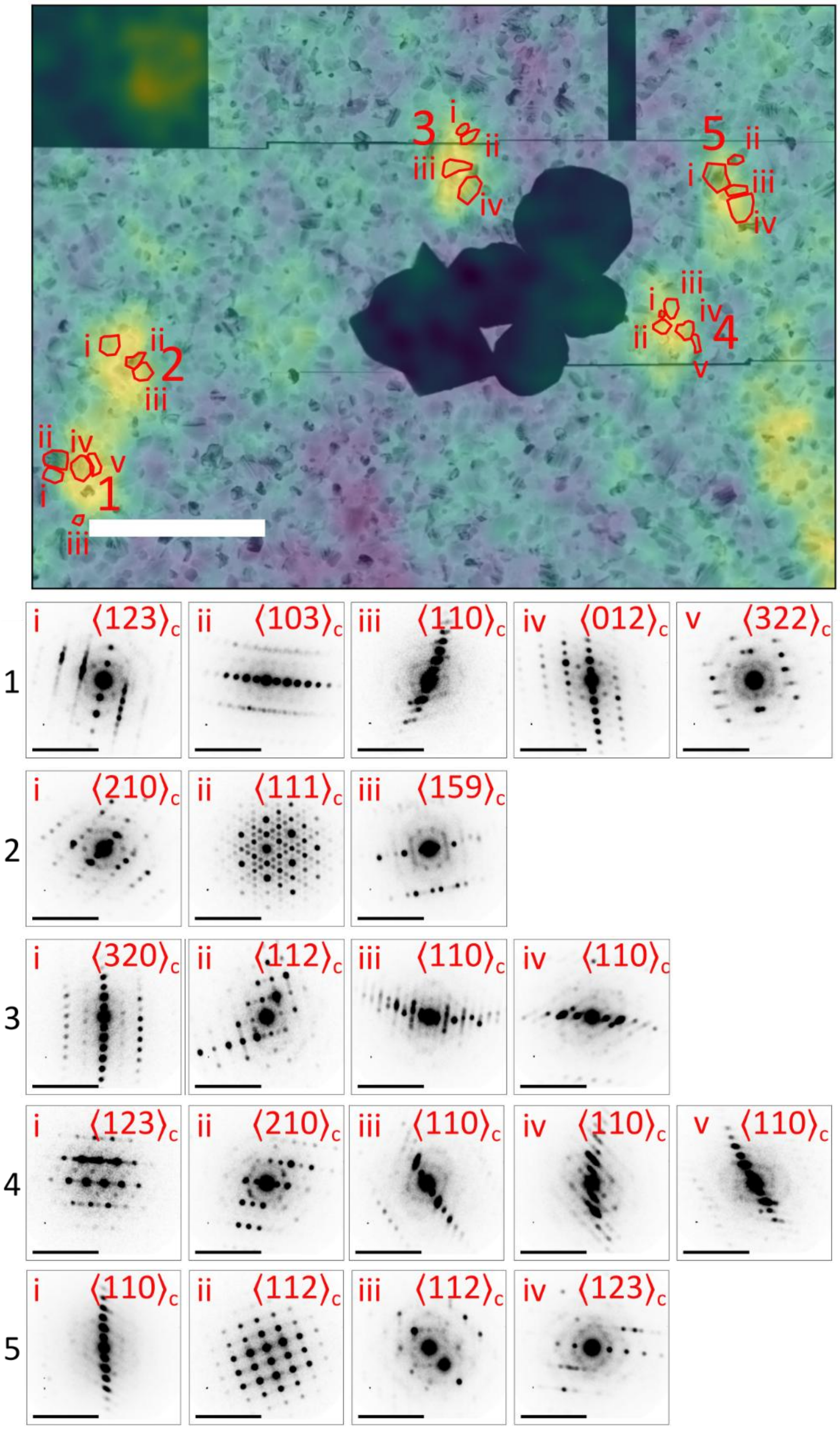

Supplementary Fig. 27 | The overlaid SED and hyperspectral PL data taken at 706 nm emission for the region shown in main text Fig. 4, shown for thermally evaporated $FAPbI_3$ on SiN_x grids. The hyperspectral PL data has undergone a gaussian blur to remove high frequency noise from the image. Each cluster of grains marked is accompanied with the diffraction patterns shown below, indexed to a pseudocubic unit cell. Scale bars are 2 µm and 1Å^{-1}.

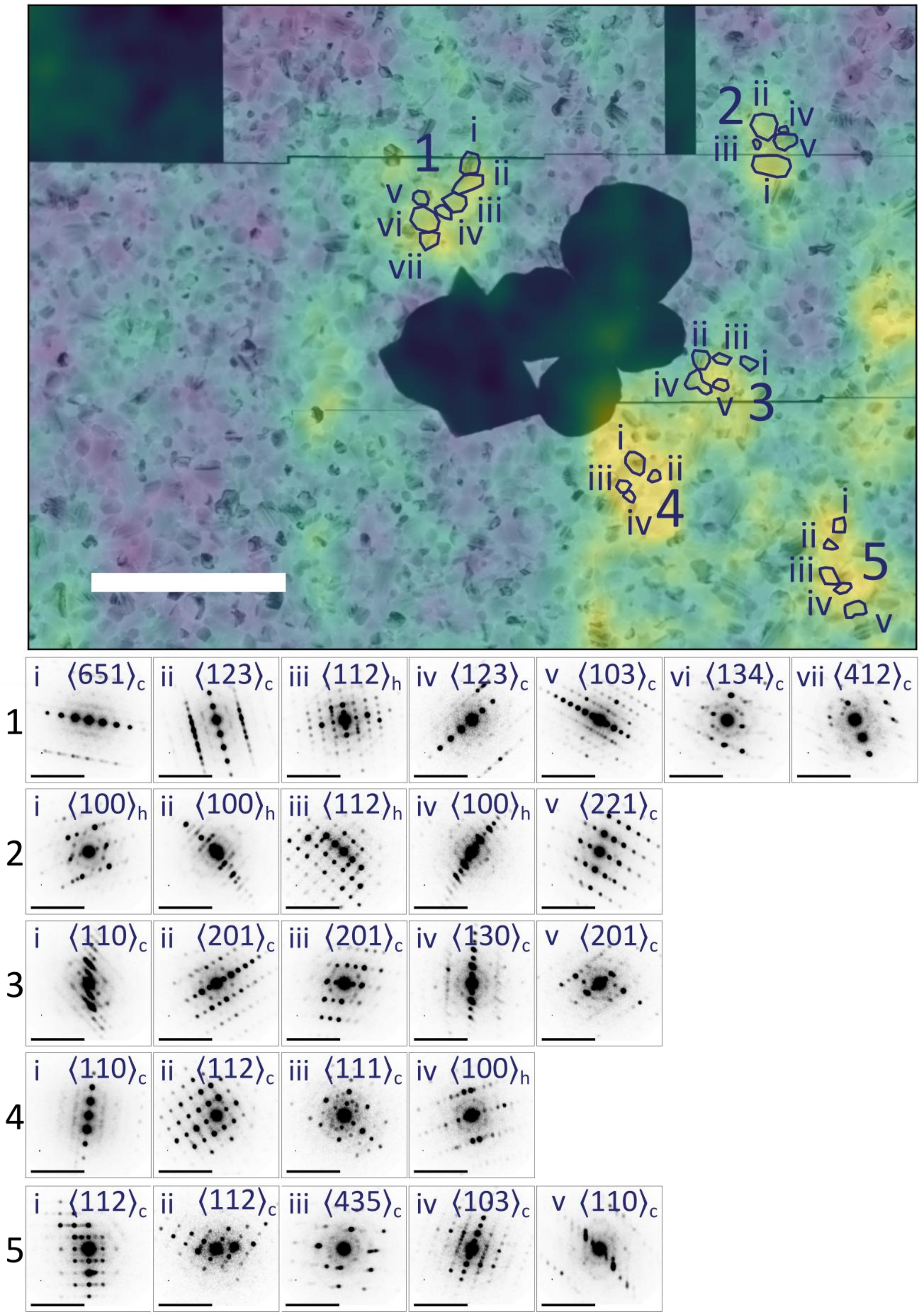

Supplementary Fig. 28 | The overlaid SED and hyperspectral PL data taken at 734 nm emission for the region shown in main text Fig. 4, shown for thermally evaporated $FAPbI_3$ on SiN_x grids. The hyperspectral PL data has undergone a gaussian blur to remove high frequency noise from the image. Each cluster of grains marked is accompanied with the diffraction patterns shown below, indexed to a pseudocubic unit cell. Scale bars are 2 µm and 1 $Å^{-1}$.

Supplementary Text 1: Acquisition and preprocessing of the SED data

We note that these isolated characteristics cannot be resolved by bulk structural tools, including cryogenic X-ray diffraction (cryo-XRD), cryogenic wide-angle X-ray scattering (cryo-WAXS), and cryogenic small-angle X-ray scattering (cryo-SAXS), as shown in Supplementary Fig. 11-13. This suggests that the confinement requires a local and nanoscale structural resolution beyond these bulk structure characteristics, especially considering that quantum tunnelling typically occurs when the barrier thickness is below ~1-3 nanometres.[7,8]

Therefore, the contiguous datasets were acquired as outlined in the Materials and Methods section however the small note on electron beam damage should be expanded on from the main text. Although we are confident that damage was mitigated sufficiently and no observable changes in the SED data was observed between scans, damage mitigation is complicated by the relative cross sections between radiolysis and knock-on damage being largely unknown for this class of material. This means that measurements performed at cryogenic temperatures may either improve or worsen the effect of beam induced damage.[6,9,10]
To ascertain how $FAPbI_3$ is affected by electron beam exposure at cryogenic temperatures (98K) we perform a damage series by imaging the same area concurrently for 25 scans with a dose rate of 12.72 eÅ^{-2} per frame. From this, upon electron irradiation at high cumulative fluence we observe a direct amorphization of the structure, consistent with the reports by Rothmann *et al.*[23]. We note that nanoscale twins are present at the outset of the damage series and are not induced by the electron beam (Supplementary Fig. 29)

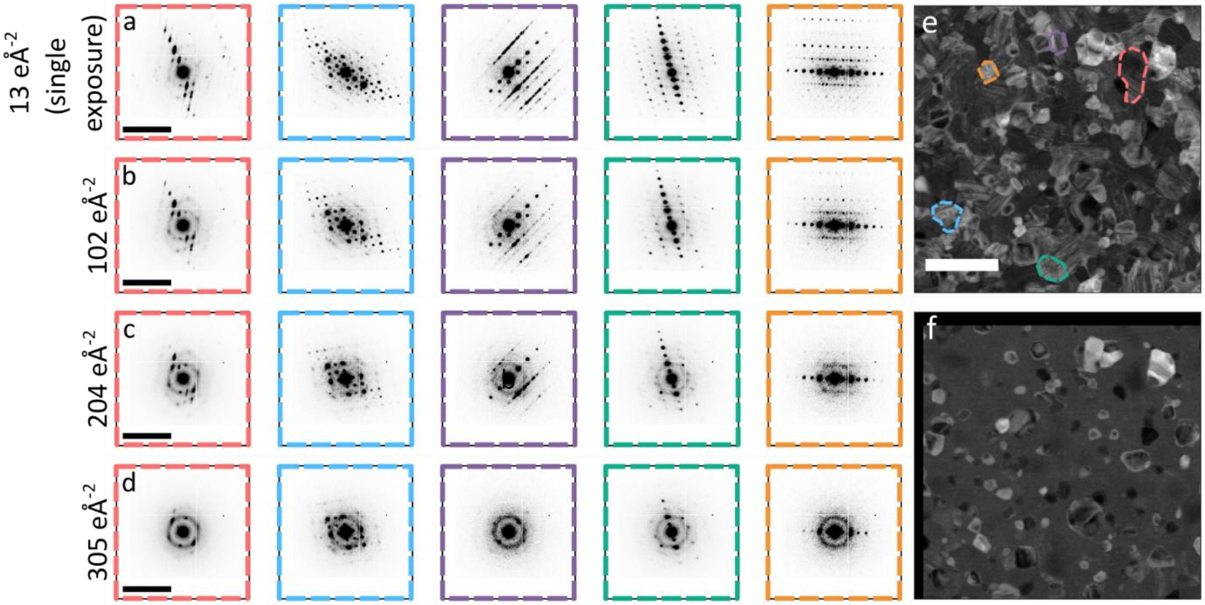

.

Supplementary Fig. 29 | The electron beam damage of evaporated $FAPbI_3$ deposited on a SiN_x TEM grid. (a-d) Averaged diffraction patterns with increasing electron dose, shown for the grains highlighted in (e). **(e & f)** Virtual darkfield images of near pristine (after a single exposure) and irradiated (cumulative dose of 305 eÅ^{-2}) $FAPbI_3$ drift corrected on top of each other. An amortization of the perovskite structure is observed whilst grains indexed to PbI2 remain largely undamaged. Scale bars 1Å^{-1} and 500 nm.

Analysis of the SED dataset was performed with the use of python packages pyxem 0.16 and py4DSTEM 0.14.[11,12] Firstly, the direct beam was aligned to the central pixel of the detector by cropping the data to the area of pixels to central spot moves over during a large area scan. The position of the direct beam was then found via pyxem and the interpolation method. The ellipticity of the polycrystalline disk was confirmed to be minimal by summing all diffraction patterns across an entire SED scan. Virtual bright field images were formed by taking intensity from a virtual aperture 0.04 $Å^{-1}$ in radius placed around the central spot. Virtual dark field images were similarly formed however a virtual aperture was placed around a diffracted reflection with a radius of 0.04 $Å^{-1}$. To kinematically simulate patterns, the software Single Crystal 5 was used. Effects on the isotropic and anisotropic displacement parameters were not considered when simulating diffraction patterns at varying temperature. As SED data are taken at low dose, diffraction patterns are often averaged over a single grain as informed by the clustering described in Supplementary Text 2. This improves the signal-to-noise ratio (Supplementary Fig 30).

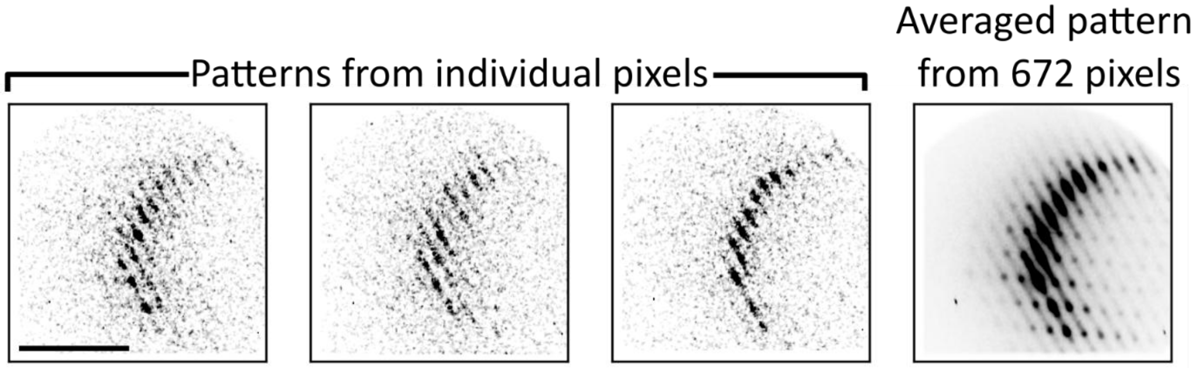

Supplementary Fig. 30 | To improve signal to noise ratio averaged diffraction patterns over single grains are presented instead of those from individual grains unless stated otherwise. Scale bar is 1$Å^{-1}$.

Supplementary Text 2: Simple linear iterative clustering (SLIC)

The clustering methodology is adapted from simple linear iterative clustering (SLIC) used widely in the field of remote sensing and is applied to high dimensional microscopy datasets herein, to our knowledge for the first time.[13,14] SLIC can be thought of as a variant to k-means clustering and provides a general, intuitive, robust, and computationally inexpensive methodology to cluster SED data (Supplementary Fig. 31). During this procedure detector pixels that have a low dynamic range or variance over the SED scan are discarded. Typically, 98% of the pixels are removed meaning only the important data points for clustering are retained. Next 700 centroid seeds (N=700 in pseudocode below) are initialized randomly or as a regular grid into the SED scan in real space. Data in the vicinity of the centroid (defined by S) is then considered and assigned to a cluster depending on the similarity to each centroid; defined via the Euclidean distance. The spatial and channel distances are then combined into a single measure via a weighting factor m, this encodes intuition that ‘pixels’ close together likely belong to the same cluster. Once this step is complete for all the data the centroids are updated and the process repeats iteratively. After the iterative process a final step which combines clusters that correlate highly with each other is performed. The mean diffraction patterns from each cluster are then computed from the original data. As much of the data prior to clustering is discarded and each

centroid only considers data in its vicinity this process is surprisingly efficient with it taking 99s on a standard desktop machine (11th Gen Intel(R) Core (TM) i5-11400 CPU and 32Gb of RAM), this could likely be further improved with by performing the calculations on a GPU.

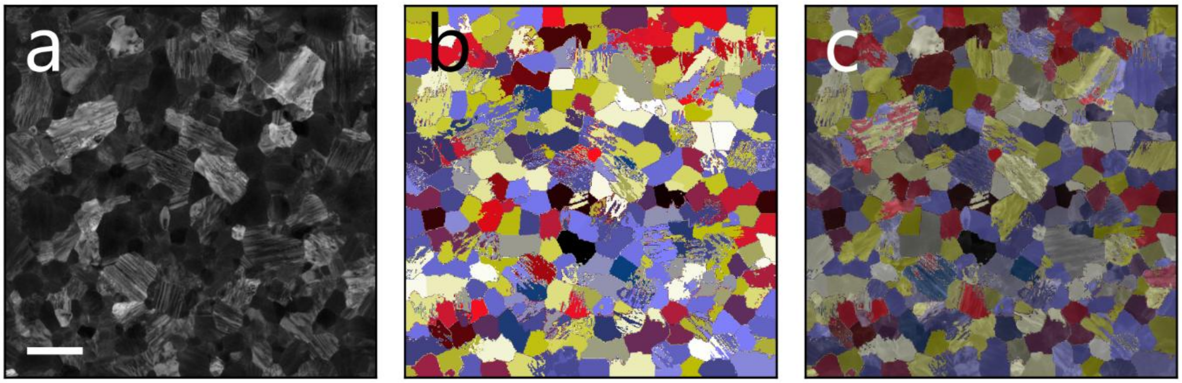

Supplementary Fig. 31 | The SLIC methodology applied to an SED scan. (a) Shows a dark field image formed from the SED scan. **(b)** Shows the resulting clusters after the SLIC clustering. **(c)** An overlay of (a) & (b). Scale bar in (a): 500 nm.

When compared to other clustering methods, such as principal component analysis (PCA) and traditional k-means clustering, the SLIC approach is confirmed to be an improvement in our case. If PCA is performed on the same SED scan as above, (after pixels which have variance below the 98th percentile are removed so that a direct comparison to SLIC can be made) the compute time is improved with the entire process taking only 33s. However, this comes at the detriment of the clustering quality. During this approach PCA is applied to the data and the components which retain up to 1% of the explained variance ratio are used as a cut-off similarly to Duran *et al.*; this leaves 28 principle components to be included for subsequent k-means clustering (Supplementary Fig. 32).[15] Once clustered we observe a drop in accuracy due to this approach not encoding the intuition that pixels in close proximity are likely to belong to the same cluster, as SLIC does. Ultimately this leads to diffraction patterns which are not from the same grain being clustered together, giving the appearance of overlapping patterns in the resulting clusters.

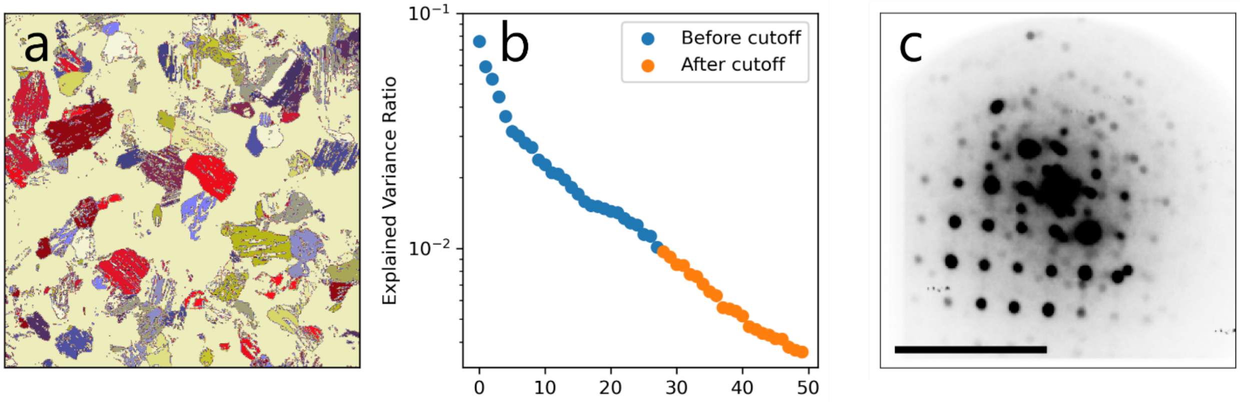

Supplementary Fig. 32 | PCA clustering results. (a) The results when PCA clustering is

performed. **(b)** A scree plot showing the cutoff when explained variance ratio is below 1%. **(c)** A mean diffraction pattern taken from a cluster in (a) where multiple crystallites are contributing to the signal. Scale bar in (c): 1$Å^{-1}$.

It is important to note, performing PCA as a dimensionality reduction technique and related unsupervised methods are undoubtedly valuable tools that are likely to outperform SLIC on different problems due to SLIC in part relying on the crystallinity and polycrystalline nature of the perovskite film.

Code used to perform SLIC can be found on Github. In pseudo code the adapted SLIC algorithm is as follow:

1. Unravel each diffraction pattern into a 1D array.
2. **Require:** dynamic range or variance of channels > 95th percentile
3. Normalise the channels between [0,1]
4. Initialize N centroids
5. **while** converged = False **do**
6. **for all** centroids ∈ {1, . . . ,N} **do**
7. **for all** 'pixels' in the vicinity (S) of each centroid **do**
8. Calculate the distance measure (D_{new}) between each 'pixel' and centroid given weighting factor (m)
9. **if** D_{new} < $D_{current}$ **then**
10. Reassign the 'pixel' to the new centroid
11. Combine clusters which are highly correlated
12. Calculate mean diffraction patterns from the original data

Supplementary Text 3: Determination of the Low Temperature Phase

The appearance of superstructure reflections upon cooling in electron diffraction patterns can be used to inform the lower symmetry space group of perovskites[16]. The methodology used in this work follows similarly to that used by Woodward *et al.*[17] and we assume O_h tilting is the only structural distortion present. Throughout the discussion we will use the Glazer notation as well as noting the space group; Glazer notation considers tilting independently about the pseudocubic $<001>_c$ directions with a superscript 0, + and - denoting no tilt, inphase and antiphase tilting respectively[18,19].

Firstly, considering the literature there already exists reports of the phase behavior of $FAPbI_3$ at low temperature, but a consistent understanding of what the space group the γ phase occupies is lacking[20–22]. This debate is undoubtedly due to the many possible lower symmetry space groups perovskites can occupy due to structural distortions of the cubic aristotype. Indeed, common causes of superstructure reflections come from octahedral tilting or cation/vacancy ordering; or indeed a combination of both. Furthermore, as prior work reported experiments either on single crystals, which are not representative of the thin films; or use powder-based techniques, which use spatial averaging and azimuthally integrated diffraction patterns, an accurate identification of the phase behavior is especially challenging. From this it is evident that scanning electron diffraction is one of only a few techniques which can achieve a genuine understanding of the phase behavior of $FAPbI_3$ thin films at low temperature.

It is well established that upon cooling at 285K the cubic aristotype (α phase, $Pm\bar{3}m$, $a^0a^0a^0$) continually converts to a lower symmetry space group with in–phase tilting occurring about a single pseudocubic axis via a second order phase transition. This gives the β phase ($P4/mbm$, $a^0a^0c^+$). Upon further cooling it is known there is a first order phase transition at ~140K to the γ phase but the identity of this phase is not entirely understood. Fabini *et al.* have found the γ phase still possesses a $P4/mbm$ symmetry using high resolution powder X-ray diffraction (P-XRD).[21] In that work the $Immm$ ($a^+b^+c^+$); $I4/mmm$ ($a^0b^+b^+$); and $Im\bar{3}$ ($a^+a^+a^+$) space groups are considered but rejected from the fact that no low angle Bragg peaks are detected as would be expected.

In other reports it was commented that the "synthesis conditions, sample environment, and cycling temperature all play a role in the observed phases of the material"[20] and showed that below 140K the motion of the FA cation is restricted, forming a glassy state. However, it is also noted that additional Bragg reflections are observed but the origin of these and therefore the space group of the γ phase was not determined. The presumption of complexity is further reinforced by work considering the cubic (α phase) to hexagonal (δ phase) upon cooling which shows kinetic trapping of the pseudocubic phase occurs depending on cooling rates[22].

Now considering the electron diffraction patterns obtained from our data. Firstly, by collating $<001>_c$ zone axis patterns we confirm no superstructure reflections are present at room temperature as expected for the cubic $Pm\bar{3}m$, $a^0a^0a^0$ structure (as shown in Supplementary Fig. 13b). Upon cooling to 90K superstructure peaks appear consistently at $\frac{1}{2}\{ooe\}_c$ positions with no absences. Where *o* and *e* denote odd and even Miller indices as described by Woodward *et al.* [17] It is important to note that if a $P4/mbm$, $a^0a^0c^+$ space group was retained at 90K it is expected to only see superstructure reflections in ⅓ of the patterns sampled and systematic absences are also

expected. Next, we consider the <111>$_c$/<001>$_h$ zone axis patterns, we again see the appearance of superstructure reflections upon cooling, at ½{ooe}$_c$ positions. This is consistent with in-phase tilting about multiple pseudocubic axes and importantly inconsistent with $P4/mbm$, $a^0a^0c^+$ even if dynamical diffraction is considered (Supplementary Fig. 33)[17]. We therefore assign the low-temperature phase of $FAPbI_3$ to be an in-phase tilt system about more than one pseudocubic axis; if we consider a perfectly corner-sharing Pb-I sublattice this would result in a space group of either $Immm$ ($a^+b^+c^+$); $I4/mmm$ ($a^0b^+b^+$); or $Im\bar{3}$ ($a^+a^+a^+$). As the appearance of superstructure reflections in these space groups is dictated by the body-centring operation, extra reflections appear at identical positions. Linking this observation to the PL and absorption spectra between ambient temperature and 4 K, where a red-shift of the band edge is observed, we can now provide a nuanced structural understanding of how this PL shift is related to octahedral tilting up until the ~80 K limit recorded with SED (Supplementary Text 3). This result is consistent with variable temperature powder XRD results where a shift in peak position is observed upon cooling attributed to structural contraction (Supplementary Fig. 11). By contrast, the quantum emission effects are linked to nanoscale twin structural features, as opposed to other structural distortions such as octahedral tilts, given that the nanoscale twins remain alike across this phase transition and that the above-bandgap quantum emission and transients are also maintained (Supplementary Figures 6a & 10h).[16,23]

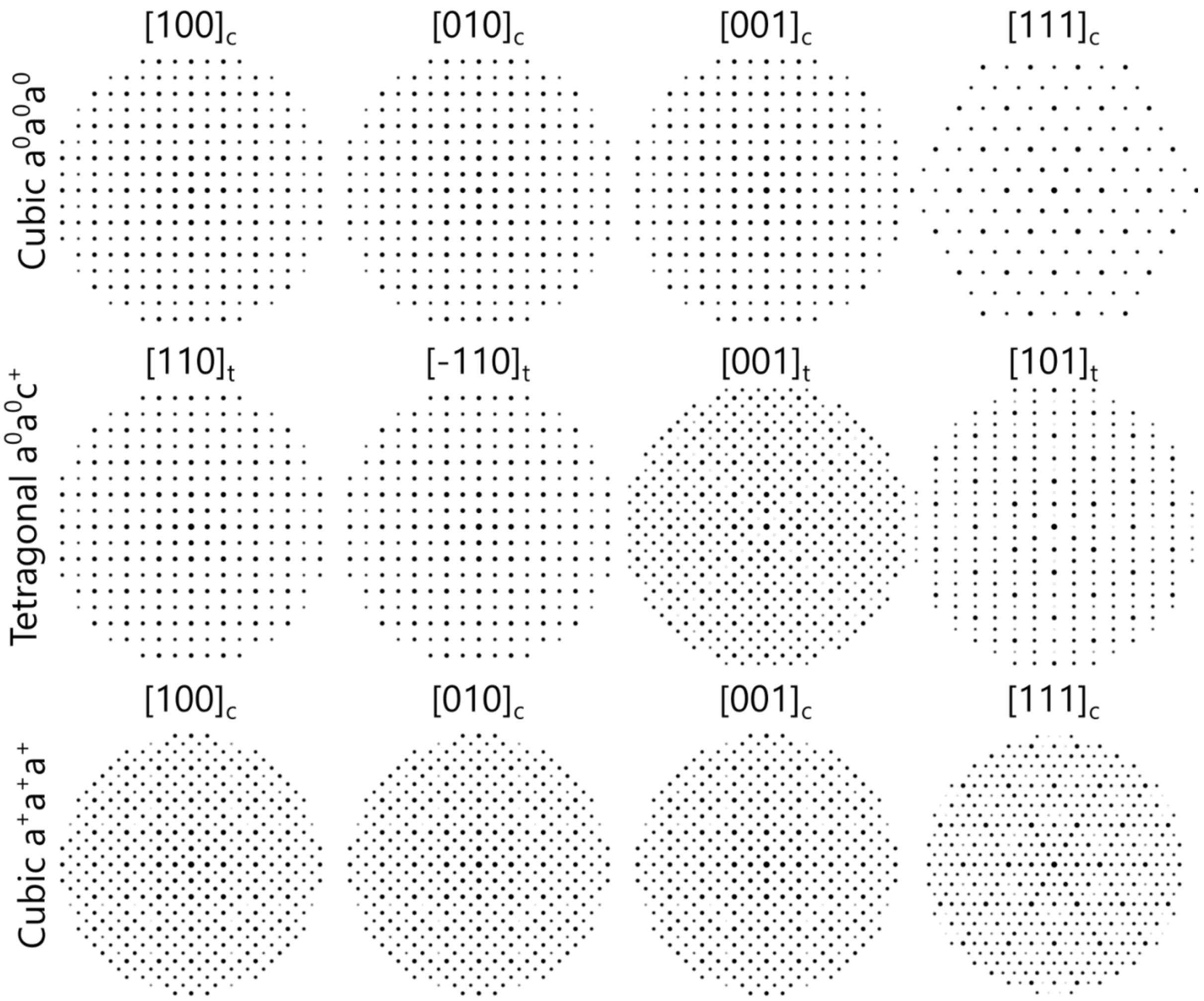

Supplementary Fig. 33 | Kinematically simulated zone axis patterns along $[100]_c$, $[010]_c$ and $[001]_c$ and $[111]_c$ directions in the pseudo cubic unit cell. The cubic $Im\bar{3}$ ($a^+a^+a^+$) space group is used to illustrate the appearance of superstructure peaks, this is equivalent in the *Immm* ($a^+b^+c^+$) and *I*4/*mmm* ($a^0b^+b^+$) tilt systems.

We attempt to see superstructure reflections consistent with our proposed structure along a $<110>_c$ ZA however do not observe any additional spots upon cooling. This is attributed to the fact that the superstructure reflections are weak compared to the background diffuse scattering observed from nanotwinning in $<110>_c$ ZA patterns. We can however consider patterns oriented along a $<123>_c$ ZA where the Bragg spots are further apart, allowing for the appearance of superstructure reflections to be observed. This is consistent with our assignment (Supplementary Fig. 34).

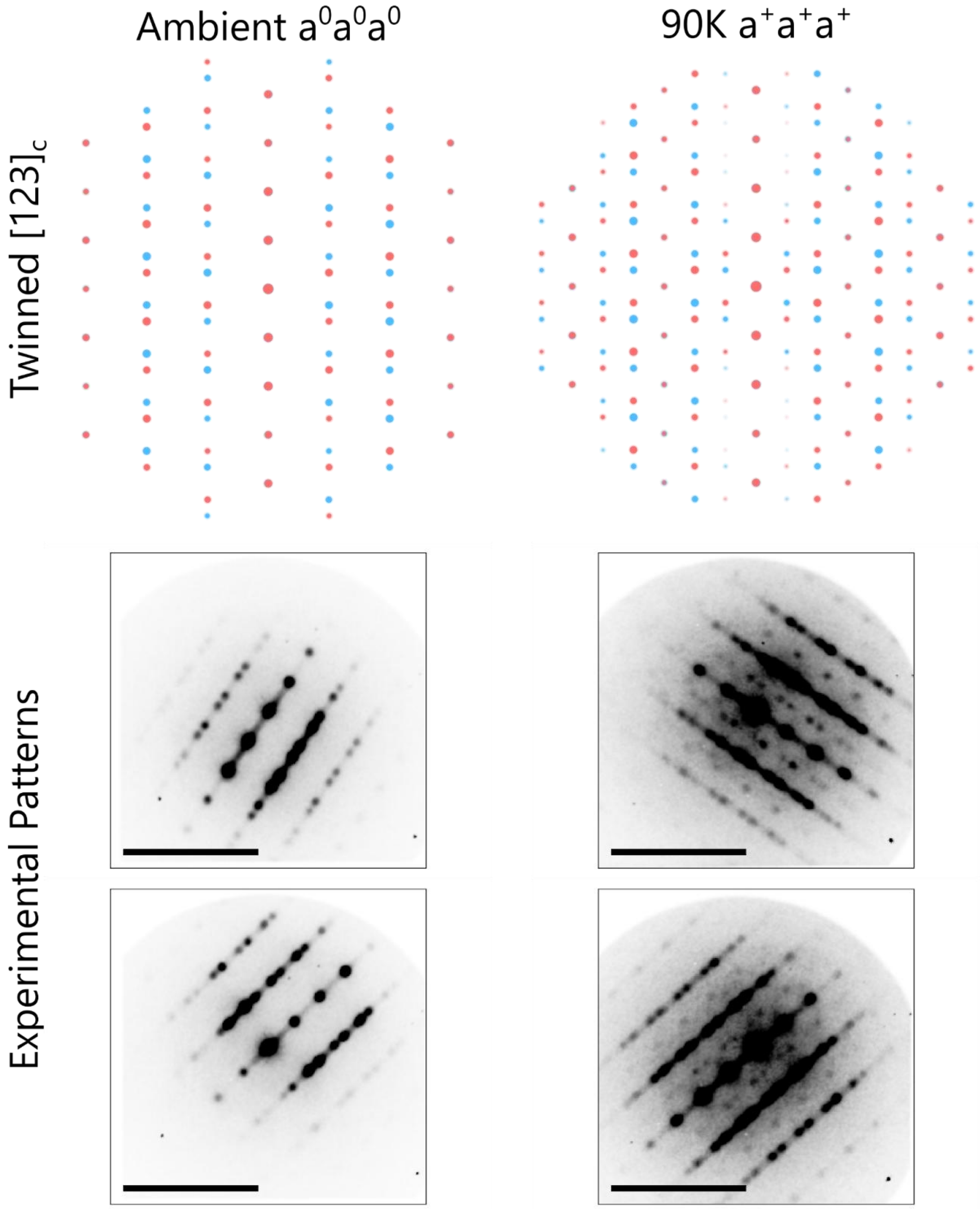

Supplementary Fig. 34 | Showing the superstructure peaks observed upon cooling if oriented along a $[123]_c$ ZA with nanotwinning. All scale bars are $1Å^{-1}$.

Considering hexagonal polytypes which can be described as a highly ordered array of nanotwins. Upon tilting of the corner sharing layers between $\{111\}_c$ twins in an $a^+a^+a^+$ fashion this results in superstructure peaks similar to those observed experimentally at ½$\{ooe\}_c$ positions (Supplementary Fig. 35). To create .cif files for the tilted hexagonal polytypes firstly the basis vectors of a parent .cif file, whether that be $a^0a^0a^0$, $a^+a^+a^+$ or $a^0a^0c^+$ were changed to possess hexagonal symmetry, with the new lattice parameter along c, denoted $[001]_h$, representing what was previously a pseudocubic $<111>_c$ direction. The order of the polytype is then defined by altering the number of corner sharing layers present and a face sharing twin boundary layer created. As we propose isolated emitters are ordered hexagonal polytypes they should not be assigned a space group of *Immm* ($a^+b^+c^+$); *I*4/*mmm* ($a^0b^+b^+$); or $Im\bar{3}$ ($a^+a^+a^+$) despite reflections consistent with in-phase tilting about multiple pseudocubic axes being present.

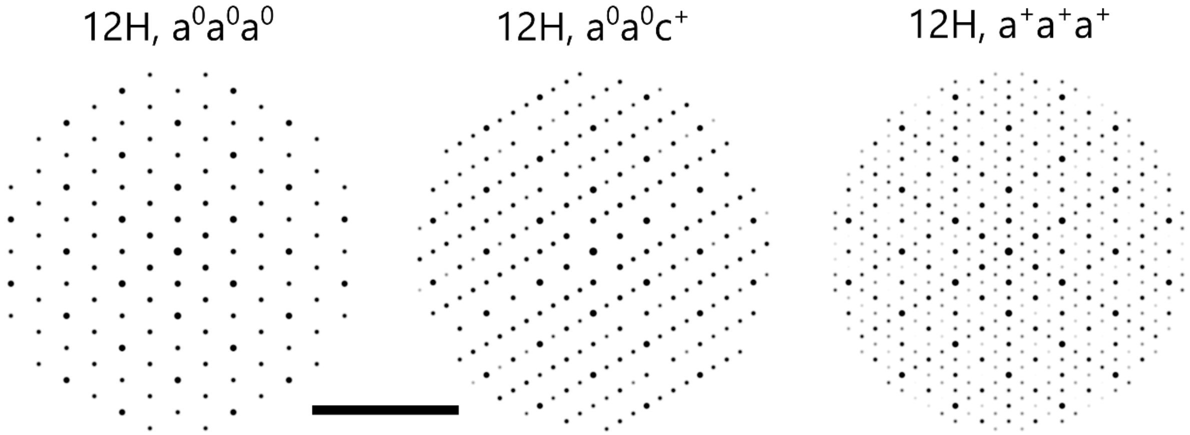

Supplementary Fig. 35 | Kinematically, simulated diffraction patterns of a 12H polytype with various tiling patterns imposed on the corner sharing layers. Scale bar: 1Å^{-1}.

If the position of $\{hh0\}_c$ reflections in the cryogenic temperature $<001>_h/<111>_c$ zone axis pattern are inspected closely it can be seen they do not noticeably distort away from the ambient equivalents. This suggests that the tilting is equal along all three pseudocubic directions within the limits of experimental error. To measure any distortion accurately a line is fit across complementary $\{hh0\}_c$ Bragg reflections and the angles between these calculated. To gain an estimate of the uncertainty of the angles we consider the case where a $\{110\}_c$, $\{220\}_c$ or $\{330\}_c$ reflection moves position by a single pixel normal to the relevant g vector. This gives shifts of 2.3°, 1.8° and 0.8° for $\{110\}_c$, $\{220\}_c$ and $\{330\}_c$ reflections respectively, as the differences in angle we observe experimentally are significantly smaller than these values we are propose 6-fold symmetry is retained within experimental error. (Supplementary Fig. 36).

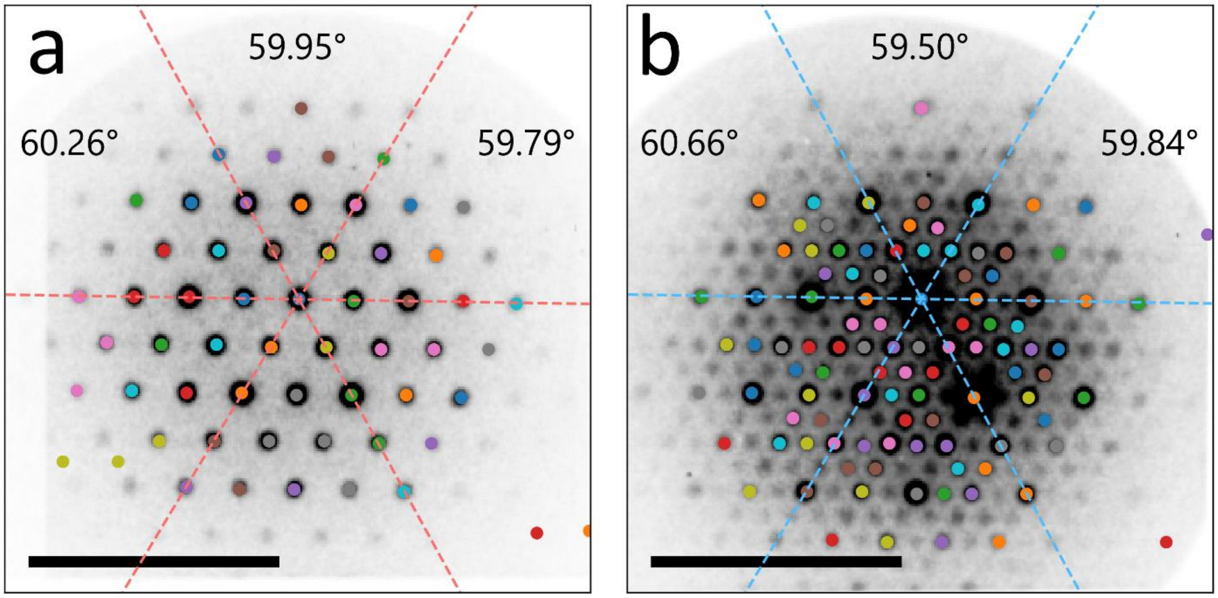

Supplementary Fig. 36 | The experimental <111>$_c$ zone axis pattern at **(a)** ambient temperature and **(b)** cryogenic temperature, with peaks found via a difference of gaussian method[11]. Overlaid over each pattern are lines fit through the peaks belonging to the {hh0}$_c$ family, the angles between these lines show minimal distortion of the 6-fold rotational symmetry upon cooling. Scale bars: 1Å^{-1}.

Symmetry breaking upon cooling is also observed in patterns which are originally indexed to a hexagonal polytype at ambient conditions. It is understandable that there are numerous hexagonal polytypes present in the film due to the ubiquitous nature of the {111}$_c$ nanotwinning present. This behaviour further reinforces the fact that in high order hexagonal polytypes the majority of octahedra are still corner sharing (except for the layers which can be considered a {111}$_c$ twin) and will similarly distort to the discussion above (Supplementary Fig. 37).

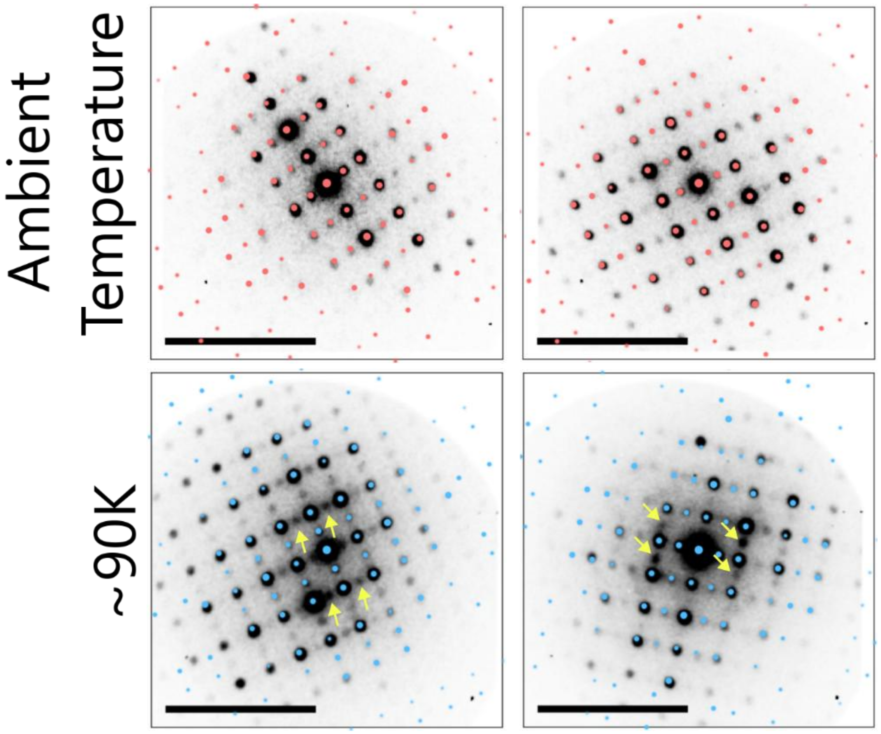

Supplementary Fig. 37 | The appearance of extra spots at low temperature (blue) compared with ambient temperature (pink) are marked by arrows and attributed to octahedral tilting of the corner sharing layers. Diffraction patterns which can be indexed to $[112]_h$ zone axis of a 6H polytype are shown.

There are several complications which mean our assignment should be considered cautiously. Firstly, dynamical diffraction will excite reflections which are nominally systematically absent, especially when close to a zone axis pattern. Secondly, due to the dynamic nature and structural heterogeneity of hybrid perovskites, defining a unifying space group may not be appropriate with phase transitions being highly dependent on the local environment[24–26], particularly with the high density of extended defects present. Despite these caveats a consistent structural model for the SED data collected herein has been proposed.

Supplementary Text 4: Stitching of the SED data and spatial correlation with hyperspectral PL

For brevity we demonstrate the stitching and correlation methodology for one of the two correlative SED datasets recorded as it is similar in both cases. In this sample we first correlated the SED data at ambient temperature before finding the corresponding structural features in the cryogenic dataset; this was done to prevent the slight drift experienced at ~90 K between scans affecting the registration.

To stitch many SED scans together we record multiple contiguous scans and form virtual bright or dark field (VBF and VDF) images which act as a proxy for the 4D dataset. A key-point detection and matching algorithm is then applied between the images; common choices for this include the scale invariant feature transform (SIFT) or binary robust invariant scalable key-points (BRISK) algorithms, implemented using OpenCV[27–29]. Although these are undoubtedly valuable tools, it has been empirically found that pretrained neural network-based approaches such as Key-Net-AdaLAM, implemented using Kornia provide improved mappings[30]. Once the keypoints are detected the random sample consensus algorithm (RANSAC), or variants thereof, is used to define an affine transform which stitches one image onto the other (Supplementary Fig. 38)[31]. As an affine transform is utilized differences in scaling, rotation, shear, and translation can be accounted for between the images. To make these tools accessible a Python based GUI has been developed which can be found on Github.

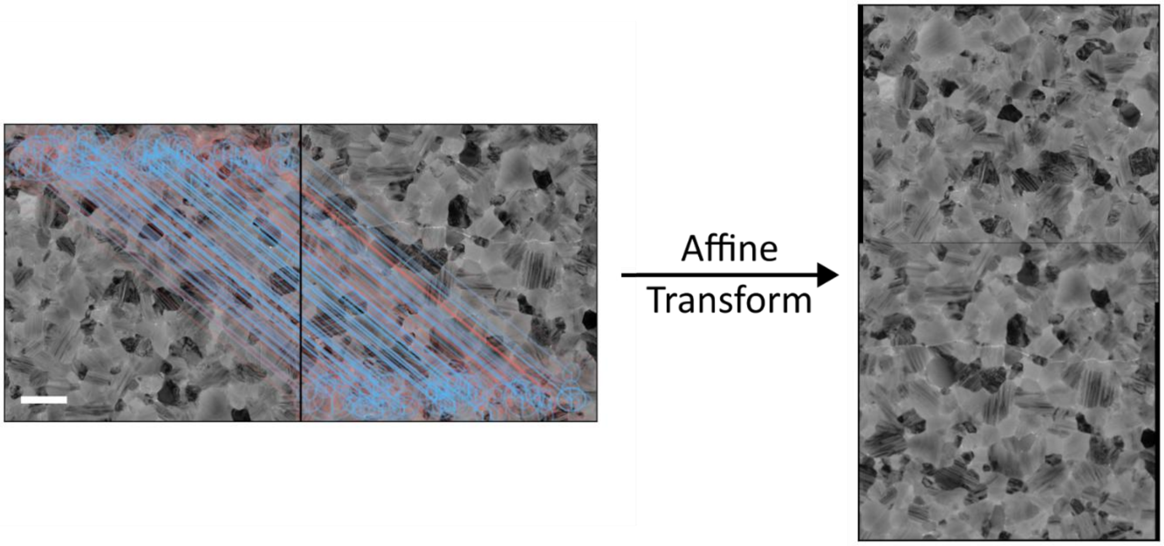

Supplementary Fig. 38 | Keypoints between the two virtual bright field images detected using AdaLAM[32], with tentative and confident matches shown in pink and blue respectively. The RANSAC algorithm is used to define an affine transform which stitches the images together. Scale bar: 500 nm.

To then perform the spatial correlation of the hyperspectral PL and SED data we use the Au fiducial marker to find a common region between the two imaging modalities (Supplementary Fig. 39).

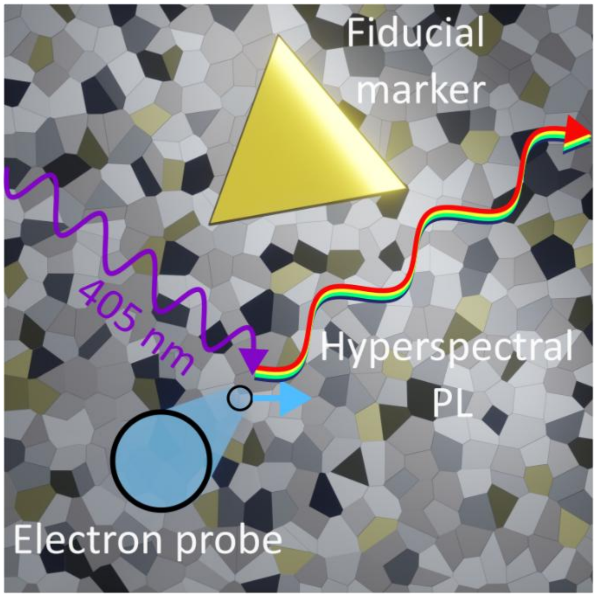

Supplementary Fig. 39 | A cartoon schematic of how to spatially correlate between hyperspectral PL microscopy and the electron microscope.

To account for differences in pixel sizes between the hyperspectral PL and SED datasets the hyperspectral PL data is upsampled using linear interpolation. Due to the different contrast mechanisms between the techniques both datasets are then cropped such that only the Au fiducial marker is used during the registration. The resultant transform to overlay the datasets can be found several ways, herein we use two independent methods and compare their output. Firstly, an image transform can be defined by finding where the normalized cross correlation (NCC) is maximal between the two images accounting for both rotation and translation (Supplementary Fig. 40 & 41). Once the transformation has been found the two datasets can then be overlaid (as shown in main text Fig. 4).

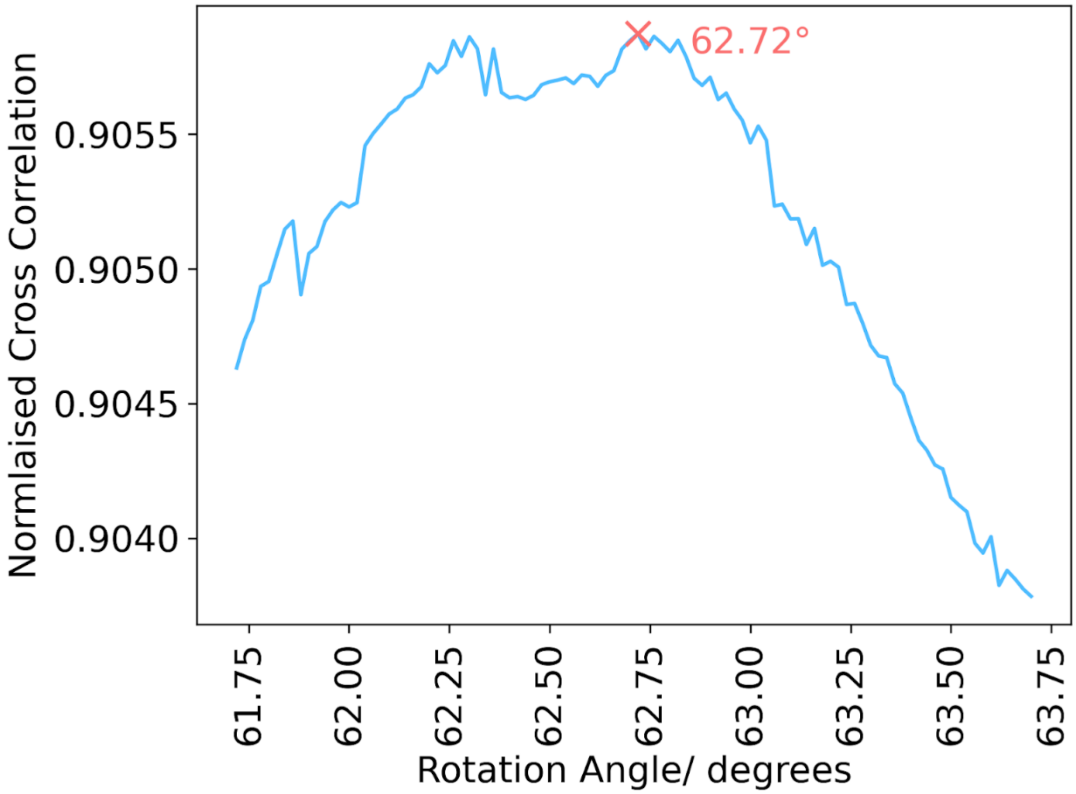

Supplementary Fig. 40 | The optimum rotation between the two datasets found to be 62.72° where the NCC is maximal

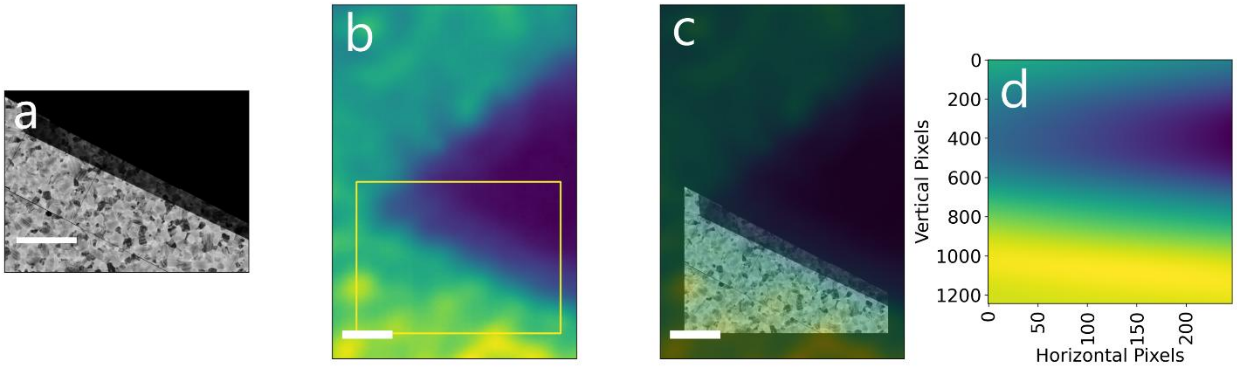

Supplementary Fig. 41 | Finding the optimal translation between the hyperspectral PL and SED. (a) The stitched and rotated (62.72°) VBF image from the SED data. **(b)** The summed hyperspectral PL data over all channels and an overlaid rectangle showing where the NCC is maximal. **(c)** An overlay of the data shown in a&b. **(d)** The values for the NCC as (a) is rastered over (b) to find the optimal overlap. All scale bars: 2µm

Secondly, the Python package AntsPy, primarily built for the registration of medical images was used to define a mapping[32,33]. AntsPy uses ‘multi-resolution gradient descent’ and metrics such as the cross correlation (CC) or mutual information (MI) to define the transformation. During this process both the SED and hyperspectral PL were cropped, as above, such that only the Au fiducial marker is used during the correlation and a ‘rigid’ transform defined so that rotation and translation were accounted for (Supplementary Fig. 42). When the two methods are compared, they are shown to be consistent, with the grain oriented close to a $<111>_c/<001>_h$ zone axis in the SED being close to the bright isolated emitter in the hyperspectral PL.

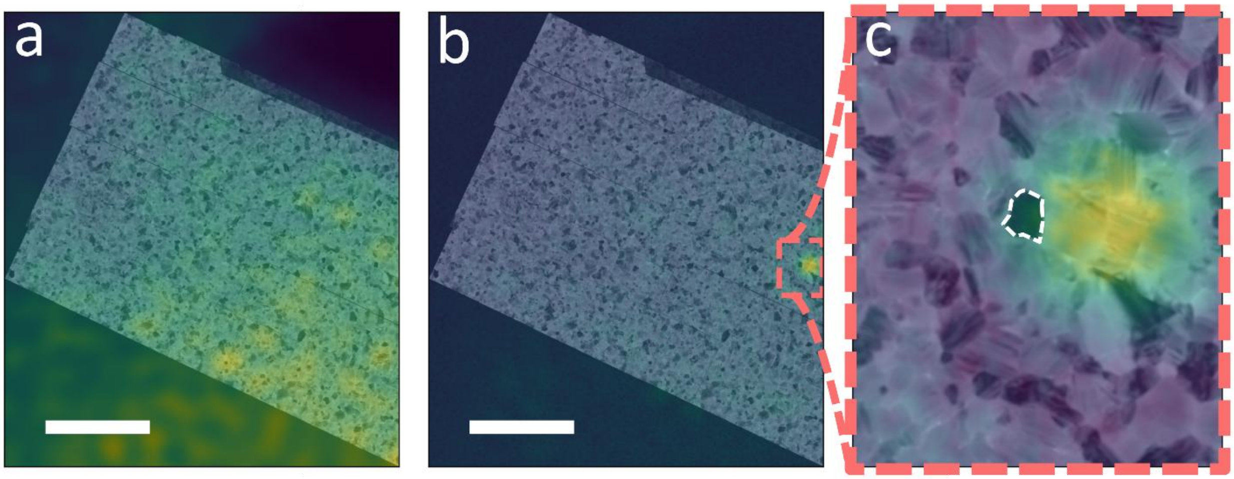

Supplementary Fig. 42 | Overlay of hyperspectral PL and SED. **(a)** Overlay of hyperspectral PL, summed over all channels, and the stitched VBF image data using the transform defined via AntsPy. **(b)** Overlay of the PL map taken at an emission wavelength of 706 nm overlaid with the stitched VBF image. **(c)** Expansion from (b) showing the grain oriented along the $<111>_c$ zone axis (marked) is in the same locality as the isolated emitter. All scale bars: 5μm.

Supplementary Text 5: Krönig-Penney superlattice

As illustrated in the main text Fig. 3j, the Krönig-Penney model describes the quantized behavior of electrons in a periodic potential. The periodical potential is constructed by δ phase as the barrier with width *b* and α phase as the well with width *a*. The E-k relation can be expressed by[34]:

$$\begin{cases} \cos k(a+b) = \dfrac{1-2\zeta}{2\sqrt{\zeta(1-\zeta)}} \sin\left(\alpha_0 a\sqrt{\zeta}\right)\sinh\left(\alpha_0 b\sqrt{1-\zeta}\right) \\ \qquad + \cos\left(\alpha_0 a\sqrt{\zeta}\right)\cosh\left(\alpha_0 b\sqrt{1-\zeta}\right) \\ \text{for } 0 < \zeta < 1 \\ \cos k(a+b) = \dfrac{1-2\zeta}{2\sqrt{\zeta(\zeta-1)}} \sin\left(\alpha_0 a\sqrt{\zeta}\right)\sin\left(\alpha_0 b\sqrt{\zeta-1}\right) \\ \qquad + \cos\left(\alpha_0 a\sqrt{\zeta}\right)\cos\left(\alpha_0 b\sqrt{\zeta-1}\right) \\ \text{for } 1 < \zeta \end{cases}$$

Eq.S1

where,

$$\alpha_0 = \sqrt{\frac{2mU_0}{\hbar^2}} \text{ and } \zeta = \frac{E}{U_0},$$

In the given equations, the energy *E* is the sole variable in the functions on the right-hand side, whereas the wavenumber *k* is the only variable on the left-hand side. Analogous to the scenario of a finite potential well, a solution for *ζ* in Eq. S1 enables us to ascertain the energy values and the wavefunctions (once they are normalized). Considering the spacing of octahedral layers along a <111>$_c$ direction, we can get the form of the function on the right-hand side of Eq. S1, referred to as *f(ζ)*, can be depicted in Supplementary Fig. 43.

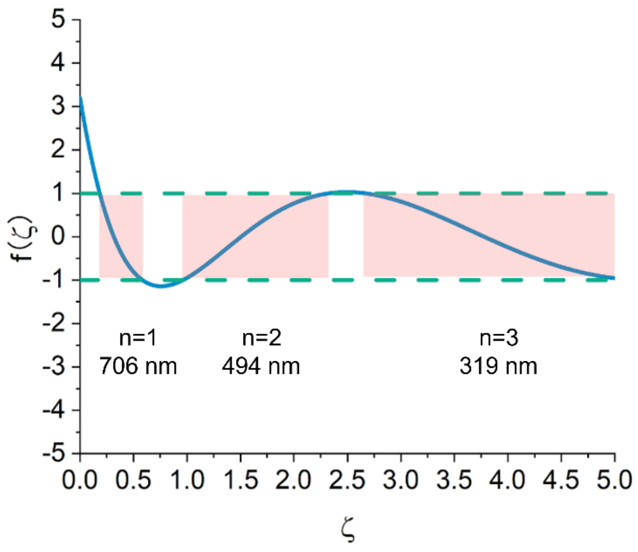

Supplementary Fig. 43 | The bands calculated from the KP model. The plot of f as a function of $\zeta = \frac{E}{U_0}$ shows the allowed energy bands (coloured area) from the quantum confinement of a superlattice described by a Krönig-Penney model. Wavelengths of each minibands are noted.

We emphasize that even in a periodic superlattice, a particle-in-a-box model should be used instead of the KP model when each barrier is substantially larger than the bound-state penetration depth or the quantum tunneling depth, which does not exceed 3 nm.[32] Since our barrier is less than 2 nm, the KP model is the most appropriate choice.

In the following calculations, we only consider energy level of n=1, i.e. the first allowed band from the KP model and obtain the quantum energy levels by layer (L) progression for the following reasons:

1. PL is in general only be observed at the first quantum state, n=1, as demonstrated in III-V systems.[33] Therefore, there must be an n=1 state at each peak to generate PL. Moreover, as shown in Supplementary Figure 36, the n=2 peak in our model, derived from the polytype parameters, lies beyond our probe range. This further confirms that the observed TAS signals also only originate from the n=1 state.
2. As visualised in Fig. 3, generally well and barrier widths are aperiodic and vary throughout the film. This means that the dimension-determined quantum energy levels from n-index progression is expected to change from grain to grain, leading to various peak positions for the same quantum number which is contrary to the fixed peak positions at a certain temperature observed experimentally in Fig.1 and 2.
3. The match in the L progression has a corresponding polytype supporting it, whereas n-index progression requires a fixed L, for which there is no experimental evidence. Furthermore, n-index progression in a single-confinement risks relying on artificial “parameter tuning,” where specific well and barrier sizes are selectively chosen to make peak alignment.
4. The L progression does not require additional parameters, such as the offset of 70 meV and the half-quantum number between n=12 and 13 needed for n-index progression.[9]
5. As shown in Fig. 1d and Supplementary Fig.6c, the individual quantum levels do not share ground states, suggesting that the quantum levels are not from progression in the same confinement.

The KP lattice constructions corresponding to each energy level, collected from the simulation, are listed in table S1 with u=0.37 nm. We recognize the well/barrier combination of 4u/2u and 7u/2u as 12H and 18H, respectively. In the calculation, the input parameters include bandgaps (2.14eV for δ-phase and 1.52eV for the black absorbing phase), effective mass, and thickness of well and barriers. Owing to the uncertainty of the exact electron affinity of δ-phase and α phase, we consider the upper and lower bound as 0.62 and 0.31 eV. To estimate reasonable values for unknown variables we iterated through the calculation whilst altering unknown parameters in line with the variation reported in the literature. From this we found that the outcome of the KP model is particularly sensitive to effective mass. For example, when we implement the same well and barrier combination, like 12H, but different values of effective mass, m* as $0.27m_0$ or $0.21m_0$, the calculated first discrete energy level varies from 723 nm to 706 nm. This encompasses the emission we observe experimentally but, due to the idealized nature of the KP model and uncertainty in the value m*, variation between experimental data and theoretically

calculated values is to be expected. Although this makes it difficult to conclusively assign a specific quantum peak to a specific polytype structure, the model demonstrates that the experimental values can be reproduced using physically meaningful values, and thus the proposal is consistent with experiment, as exampled in Supplementary Table 1.

Supplementary Table 1 Simulated well and barrier lengths corresponding to each quantum peaks.

	m^*=0.1m_0, U_0=0.62 eV		m^*=0.27m_0, U_0=0.31 eV		m^*=0.21m_0, U_0=0.31 eV	
Peaks (nm)	Well length (n_1*u) (nm)	Barrier length ((2 + n_2) *u) (nm)	Well length (n_1*u) (nm)	Barrier length ((2 + n_2) *u) (nm)	Well length (n_1*u) (nm)	Barrier length ((2 + n_2) *u) (nm)
632	4	2+1	2	2+0	2	2+1
662	5	2+0	2	2+1	3	2
689	6	2+0	3	2+0	3	2+1
706	**7**	**2+0**	3	2+1	**4**	**2+0**
723	8	2+0	**4**	**2+0**	4	2+2
736	9	2+0	4	2+2	5	2+1
748	10	2+0	5	2+1	6	2+0
756	11	2+0	6	2+0	**7**	**2+0**

To accommodate shorter wavelengths, smaller well and barrier widths are required. Therefore, we highlight that the column with U_0 = 0.62 eV represents the most reasonable case, as the other two cannot provide smaller well and barrier widths. Based on this parameter set, we plot the comparison between the layer (L) progression across various polytypes (as proposed) and the n-index progression within a fixed polytype in Supplementary Fig. 40. Although the KP model is more complicated than the infinite quantum well model (where $E_n = \frac{h^2 n^2}{8m^* L^2}$), introducing the latter as a reference is still useful for understanding the progression of quantum levels by n-index or L, as shown by the fits in Supplementary Fig. 40. As shown in Supplementary Fig. 40a, even if the barrier width is doubled – as can occur in reality – the quantum levels remain close, despite requiring a well thickness one step higher for the match. We further note that as the well width decreases the difference between having one or two twin barriers becomes more pronounced. This may provide an explanation for the unexplained splitting observed by Wright *et al.*[9]at peaks at higher energy (Supplementary Fig. 40b). Considering the alternative option of a fixed L and the progression arising from different n values, while a plausible match is achievable with fixed values of well (38 nm) and barrier (1 nm) widths, this model fails to account for any structural variations in well width.

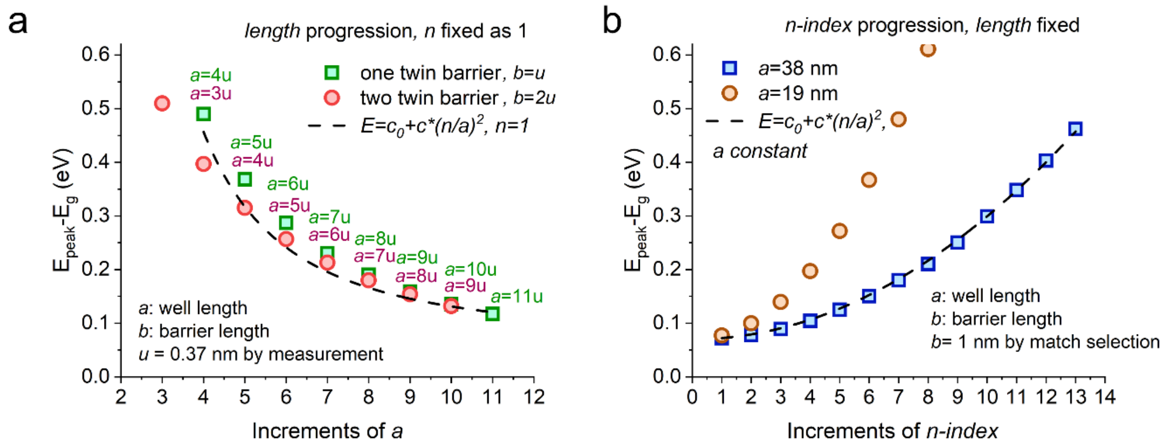

Supplementary Fig. 44 | Comparison between varying the well and barrier widths across various polytypes to an *n*-index progression within a fixed superlattice. To compare with the *n*-index progression calculation from Ref. 9, we here use a common effective mass $m^*=0.1m_0$. (a) L progression, n-index is fixed as 1 for the first quantum state. (b) n-index progression, L is fixed as the value from Ref. 9.

Supplementary Text 6: The mean lifetime of the excited carriers in the quantum levels.

The following calculation is based on a single quantum well under WKB method[35]. The mean lifetime of an electron with energy of 1.756 eV stays at its excited energy level before tunnelling out through the edge of quantum confinement can be estimated by a semi-classical computation. In the first approximation, the ratio of ψ at the outside edge of wall to escape, x_1 and at the inside edge of the wall, x_0, is

$$\Psi(x_1) = \Psi(x_0)exp\left(\frac{i}{\hbar}\int_{x_0}^{x_1} i\{2m^*[V(x)-E]\}^{1/2}\,dx\right) \equiv \Psi(x_0)e^{-\gamma/2}$$

where $\hbar$ is the reduced Planck constant, m^* is the effective mass of the electron, E is the energy of the electron, V is the height of the potential barrier, $x_1 - x_0$ means the thickness of the barrier, and *V(x)* is the potential energy at position *x*. The second part represents the probability of the particle tunnelling through the potential barrier. The first part is the decay constant, which is a measure of how quickly the wave function decays inside the barrier. It comes in a semi-classical way that the electron inside the well has a kinetic energy $T = E_{above-well} + V_0$, so its velocity is $v = [2m^*T]^{1/2}$ and it bangs against the wall at a frequency $f = {}^{v}/_{2L}$, where L is the total superlattice length. Upon collision, there is an escape probability by quantum tunnelling $e^{-\gamma}$. As a result, the probability of escape rate is

$$R = \frac{[2m^*T]^{1/2}}{2mL}e^{-\gamma}$$

Substituting the given values from our experimental measures, L=200 nm, $x_1 = 2 \times 0.37\ nm$, along with $m^* = 0.21m_0$, we find a match between the recorded lifetime and calculated results:

for peak at 706 nm, $T = 1.756\ eV$, the mean lifetime is $\tau = {}^{1}/_{R} = 2.0\ ps$;

for peak at 756 nm, $T = 1.64\ eV$, the mean lifetime is $\tau = {}^{1}/_{R} = 2.8\ ps$.

This is in accordance with the slight increase in the ultrafast component of the decays measured at a peak with a longer wavelength, as shown in Fig. 1f and Supplementary Fig. 5d.

We can also evaluate the critical width of tunnelling ($1/e^2$) by the WKB method using the formula

$d = {}^{1}/_{\kappa}$, where κ is the decay constant $\kappa = \frac{\sqrt{2m^*[V(x)-E]}}{\hbar}$

Inputting the parameters above, we can get the approximate bound state penetration depth for peak at 706 nm as 0.7 – 1 nm, close to that of III-V system.[36]

References

1. Chiang, Y.-H., Anaya, M. & Stranks, S. D. Multisource Vacuum Deposition of Methylammonium-Free Perovskite Solar Cells. *ACS Energy Lett.* **5**, 2498–2504 (2020).

2. Dai, L. *et al.* Slow carrier relaxation in tin-based perovskite nanocrystals. *Nat. Photonics* **15**, 696–702 (2021).

3. Weller, M. T., Weber, O. J., Frost, J. M. & Walsh, A. Cubic Perovskite Structure of Black Formamidinium Lead Iodide, α-$[HC(NH_2)_2]PbI_3$, at 298 K. *J. Phys. Chem. Lett.* **6**, 3209–3212 (2015).

4. Li, Z., Park, J.-S. & Walsh, A. Evolutionary exploration of polytypism in lead halide perovskites. *Chem. Sci.* **12**, 12165–12173 (2021).

5. Li, Z., Park, J.-S., Ganose, A. M. & Walsh, A. From Cubic to Hexagonal: Electronic Trends across Metal Halide Perovskite Polytypes. *J. Phys. Chem. C* **127**, 12695–12701 (2023).

6. Ferrer Orri, J. *et al.* Unveiling the Interaction Mechanisms of Electron and X-ray Radiation with Halide Perovskite Semiconductors using Scanning Nanoprobe Diffraction. *Adv. Mater.* **34**, 2200383 (2022).
7. Wright, A. D. *et al.* Intrinsic quantum confinement in formamidinium lead triiodide perovskite. *Nat. Mater.* **19**, 1201–1206 (2020).
8. Akkerman, Q. A. *et al.* Controlling the nucleation and growth kinetics of lead halide perovskite quantum dots. *Science* **377**, 1406–1412 (2022).
9. Rothmann, M. U. *et al.* Structural and Chemical Changes to $CH_3NH_3PbI_3$ Induced by Electron and Gallium Ion Beams. *Adv. Mater.* **30**, 1800629 (2018).
10. Li, Y. *et al.* Unravelling Degradation Mechanisms and Atomic Structure of Organic-Inorganic Halide Perovskites by Cryo-EM. *Joule* **3**, 2854–2866 (2019).
11. Francis, C. & Voyles, P. M. pyxem: A Scalable Mature Python Package for Analyzing 4-D STEM Data. *Microsc. Microanal.* **29**, 685–686 (2023).
12. Cautaerts, N. *et al.* Free, flexible and fast: Orientation mapping using the multi-core and GPU-accelerated template matching capabilities in the Python-based open source 4D-STEM analysis toolbox Pyxem. *Ultramicroscopy* **237**, 113517 (2022).
13. Shi, Y., Wang, W., Gong, Q. & Li, D. Superpixel segmentation and machine learning classification algorithm for cloud detection in remote-sensing images. *J. Eng.* **2019**, 6675–6679 (2019).
14. Achanta, R. *et al.* SLIC Superpixels Compared to State-of-the-Art Superpixel Methods. *IEEE Trans. Pattern Anal. Mach. Intell.* **34**, 2274–2282 (2012).
15. Duran, E. C. *et al.* Correlated electron diffraction and energy-dispersive X-ray for automated microstructure analysis. *Comput. Mater. Sci.* **228**, 112336 (2023).

16. Doherty, T. A. S. *et al.* Stabilized tilted-octahedra halide perovskites inhibit local formation of performance-limiting phases. *Science* **374**, 1598–1605 (2021).

17. Woodward, D. I. & Reaney, I. M. Electron diffraction of tilted perovskites. *Acta Crystallogr. B* **61**, 387–399 (2005).

18. Howard, C. J. & Stokes, H. T. Group-Theoretical Analysis of Octahedral Tilting in Perovskites. *Acta Crystallogr. B* **54**, 782–789 (1998).

19. Glazer, A. M. The classification of tilted octahedra in perovskites. *Acta Crystallogr. B* **28**, 3384–3392 (1972).

20. Weber, O. J. *et al.* Phase Behavior and Polymorphism of Formamidinium Lead Iodide. *Chem. Mater.* **30**, 3768–3778 (2018).

21. Fabini, D. H. *et al.* Reentrant Structural and Optical Properties and Large Positive Thermal Expansion in Perovskite Formamidinium Lead Iodide. *Angew. Chem. Int. Ed.* **55**, 15392–15396 (2016).

22. Chen, T. *et al.* Entropy-driven structural transition and kinetic trapping in formamidinium lead iodide perovskite. *Sci. Adv.* **2**, e1601650 (2016).

23. Yang, T. C.-J. *et al.* Incorporating thermal co-evaporation in current-matched all-perovskite triple-junction solar cells. *EES Sol.* **1**, 41–55 (2025).

24. Frohna, K. *et al.* Nanoscale Chemical Heterogeneity Dominates the Optoelectronic Response over Local Electronic Disorder and Strain in Alloyed Perovskite Solar Cells. *ArXiv210604942 Cond-Mat Physicsphysics* (2021).

25. Weadock, N. J. *et al.* The nature of dynamic local order in CH3NH3PbI3 and CH3NH3PbBr3. *Joule* **7**, 1051–1066 (2023).

26. Dubajic, M. *et al.* Dynamic Nanodomains Dictate Macroscopic Properties in Lead Halide Perovskites. (2024) doi:10.48550/ARXIV.2404.14598.

27. Leutenegger, S., Chli, M. & Siegwart, R. Y. BRISK: Binary Robust invariant scalable keypoints. in *2011 International Conference on Computer Vision* 2548–2555 (IEEE, Barcelona, Spain, 2011). doi:10.1109/ICCV.2011.6126542.

28. Shabunin, M. CiteOpenCV. (2017).

29. Lowe, D. G. Distinctive Image Features from Scale-Invariant Keypoints. *Int. J. Comput. Vis.* **60**, 91–110 (2004).

30. Riba, E., Mishkin, D., Ponsa, D., Rublee, E. & Bradski, G. Kornia: an Open Source Differentiable Computer Vision Library for PyTorch. Preprint at https://doi.org/10.48550/ARXIV.1910.02190 (2019).

31. Liu, J. & Bu, F. Improved RANSAC features image-matching method based on SURF. *J. Eng.* **2019**, 9118–9122 (2019).

32. Tustison, N. J. *et al.* The ANTsX ecosystem for quantitative biological and medical imaging. *Sci. Rep.* **11**, 9068 (2021).

33. Frohna, K. *et al.* Multimodal operando microscopy reveals that interfacial chemistry and nanoscale performance disorder dictate perovskite solar cell stability. (2024) doi:10.48550/ARXIV.2403.16988.

34. Razeghi, M. *Fundamentals of Solid State Engineering*. (Springer International Publishing, Cham, 2019). doi:10.1007/978-3-319-75708-7.

35. Shankar, R. *Principles of Quantum Mechanics*. (Plenum Press, 1994).

36. Dingle, R., Wiegmann, W. & Henry, C. H. Quantum States of Confined Carriers in Very Thin Al x Ga 1 − x As -GaAs- Al x Ga 1 − x As Heterostructures. *Phys. Rev. Lett.* **33**, 827–830 (1974).
